# Regulation of Inducible Nitric Oxide Synthase (NOS2) Expression in Healthy and Inflamed Bowel: A Narrative Review

**DOI:** 10.3390/ijms27188359

**Published:** 2026-09-19

**Authors:** Małgorzata Krzystek-Korpacka, Andrzej Korpacki, Adam Wąsowicz, Katarzyna Neubauer

**Affiliations:** 1 Department of Biochemistry and Immunochemistry, Wroclaw Medical University, 50-368 Wroclaw, Poland; andrzej.korpacki@student.umw.edu.pl (A.K.); adam.wasowicz@student.umw.edu.pl (A.W.); 2 Department of Gastroenterology, Hepatology, and Internal Medicine, Wroclaw Medical University, 50-556 Wroclaw, Poland; katarzyna.neubauer@umw.edu.pl

**Keywords:** Crohn’s disease, ulcerative colitis, oxidative and nitrosative stress, reactive nitrogen species, intestinal barrier, colitis-associated cancer

## Abstract

Crohn’s disease and ulcerative colitis, the principal forms of inflammatory bowel disease (IBD), are chronic inflammatory disorders characterized by recurrent intestinal injury, impaired mucosal healing, and substantial disease burden. Despite significant therapeutic advances, many patients fail to achieve sustained remission, highlighting the need for a better understanding of the molecular mechanisms driving intestinal inflammation. Nitric oxide (NO) is a key regulator of intestinal homeostasis, influencing epithelial barrier integrity, vascular function, host defense, and immune responses. In IBD, dysregulated production of NO, largely attributable to inducible nitric oxide synthase (NOS2), has been associated with both protective and pathogenic effects. Accumulating evidence indicates that the biological consequences of NOS2 activation depend on the cellular source of NO, local microenvironmental signals, and disease context. This review summarizes current knowledge on the transcriptional, epigenetic, post-transcriptional, translational, and post-translational mechanisms regulating NOS2 expression and activity in the intestine. Particular emphasis is placed on cell-specific regulation in epithelial, stromal, endothelial, neural, and immune-cell populations, as well as emerging insights from single-cell and spatial transcriptomic studies. Collectively, available evidence supports a context-dependent role for NOS2 in IBD and highlights the importance of cell-specific approaches for future biomarker development and therapeutic targeting.

## 1. Introduction

### 1.1. Nitric Oxide Synthases (NOS)

NOS2, also known as inducible NOS (iNOS; EC 1.14.13.39), is one of three NOS isoenzymes present in humans. The other two are NOS1, also referred to as neuronal NOS (nNOS), and NOS3, known as endothelial NOS (eNOS). More recently, a fourth NOS form associated with the inner mitochondrial membrane has been described and termed mitochondrial NOS (mtNOS) [1].

In the presence of NADPH, NOS enzymes catalyze the synthesis of nitric oxide (NO) from L-arginine and molecular oxygen, generating L-citrulline as a coproduct. This reaction proceeds in two steps: first, L-arginine is hydroxylated to N-hydroxy-L-arginine, which is subsequently oxidized to yield NO and L-citrulline [2,3,4]. Active NOS functions as a homodimer, with each monomer comprising a reductase domain and an oxygenase domain. NOS enzymes contain covalently bound heme as well as the flavin cofactors FAD and FMN. In addition, their activity requires tetrahydrobiopterin (BH4), which is positioned between the two domains of a monomer but interacts with amino acids of the opposing monomer, thereby contributing to dimer stabilization [5]. Dimer formation is further stabilized by a Zn^2+^ ion coordinated by four cysteine residues, two contributed by each monomer [6]. NO synthesis requires cooperation between both monomers. Electron transfer from NADPH proceeds through FAD and FMN in the reductase domain of one monomer to the heme iron located in the oxygenase domain of the other monomer. Despite extensive investigation, several mechanistic aspects of this reaction remain incompletely understood [7].

NOS1 and NOS3 require relatively high intracellular Ca^2+^ concentrations (~400 nM). Consequently, under physiological cytoplasmic Ca^2+^ levels (~100 nM), their rates of NO production are relatively low, earning them the designation of calcium-dependent, low-output isoenzymes. In contrast, NOS2 contains tightly bound calmodulin and requires approximately tenfold lower Ca^2+^ concentrations; therefore, calcium availability is generally not a limiting factor for its activity [2,8]. As a result, NOS2 can produce large amounts of NO continuously for extended periods, often lasting several hours, with NO synthesis ceasing only upon degradation of the enzyme protein [8]. Accordingly, NOS2 is classified as a calcium-independent, high-output isoenzyme.

NOS1 and NOS3 are constitutively expressed, whereas NOS2 expression is inducible and typically triggered by inflammatory and stress-related stimuli. All three NOS isoenzymes are expressed in intestinal epithelial cells. In the intestine, however, expression patterns do not fully conform to their classical classification. Constitutive isoenzymes may be upregulated by factors such as catecholamines, bile acids, and cytokines, whereas NOS2 is constitutively expressed due to the continuous exposure of the intestinal mucosa to microorganisms and other luminal stressors [3,4,9,10,11].

### 1.2. Protective vs. Deleterious Roles of NOS2/NO: Context Dependency

NO is a pleiotropic signaling and effector molecule whose biological effects depend on its concentration, enzymatic and cellular source, and the local microenvironment [12]. Accordingly, it can exert antioxidant or pro-oxidant, anti-inflammatory or pro-inflammatory, cytoprotective or cytotoxic, anti-apoptotic or pro-apoptotic, as well as antifibrotic or profibrotic effects. Through its impact on vascular tone, epithelial barrier function, mucosal defense, neuromuscular activity, immune regulation, and host–microbiota interactions, NO contributes substantially to intestinal homeostasis. Consequently, its biological actions have traditionally been interpreted according to the NOS isoenzyme responsible for its production. The relatively low amounts of NO generated by constitutively expressed NOS1 and NOS3 have long been associated with physiological functions. In contrast, NOS2-derived NO is produced at much higher levels during inflammation and has therefore traditionally been regarded as pro-inflammatory. However, growing evidence indicates that the biological consequences of NOS2 activation are highly context dependent and cannot be explained by a simple distinction between constitutive and inducible NOS isoenzymes (reviewed in [13,14,15,16]).

Under physiological conditions, NOS2 contributes to intestinal homeostasis and mucosal defense. Constitutive NOS2 expression has been demonstrated in intestinal epithelial cells [17], where NO participates in protection against luminal microorganisms and helps maintain balanced interactions between the mucosal immune system and intestinal microbiota [16]. During acute infection or injury, NOS2 induction forms an important component of innate immunity, as NO and its reactive derivatives inhibit microbial growth through nitrosylation of protein thiols and metal centers, thereby impairing microbial respiration, DNA replication, and essential metabolic pathways [18]. Consistent with this role, genetic ablation of *NOS2* results in bacterial overgrowth and enhanced ileal colonization by cecal bacterial species [19]. NOS2-derived NO has also been implicated in the regulation of leukocyte recruitment, modulation of T-cell responses, and promotion of epithelial restitution and wound healing [20,21,22].

Although NOS2 evolved primarily as a host-defense mechanism and is often beneficial during acute infection, excessive or sustained activation may become detrimental. Persistent production of high NO levels in chronically inflamed tissues increases its reaction with superoxide anion, generating peroxynitrite (ONOO−) and other reactive nitrogen species. These species promote lipid peroxidation, protein nitration, DNA damage, mitochondrial dysfunction, and epithelial barrier disruption [13,14].

The biological effects of NOS2 are further influenced by its cellular source. NO produced by immune cells exacerbated inflammation through macrophage activation, whereas enterocyte-derived NO attenuated colitis [23]. Similarly, hematopoietic cell-derived NOS2 contributes to leukocyte recruitment and inflammatory responses, while epithelial NOS2 appears to regulate host–microbiota interactions and mucosal homeostasis [19,21]. However, this distinction may not be absolute. Hashimoto-Hill et al. [24] showed that selective deletion of *NOS2* in intestinal epithelial cells, but not in myeloid cells, attenuated dextran sulfate sodium (DSS)-induced colitis, suggesting a pathogenic role for epithelial NOS2 in certain inflammatory contexts.

Given the broad biological activity and potential cytotoxicity of NO, especially when generated by the high-output NOS2 isoenzyme, its production must be tightly regulated [25]. Unlike NOS1 and NOS3, NOS2 is controlled predominantly at the transcriptional and post-transcriptional levels rather than through modulation of enzymatic activity [26]. In human cells, NOS2 induction typically requires the combined action of microbial signals, such as lipopolysaccharide (LPS), together with pro-inflammatory cytokines, particularly interferon-γ (IFN-γ), tumor necrosis factor-α (TNF-α), and interleukin-1β (IL-1β). Ongoing research continues to reveal additional layers of complexity in the regulation of NOS2 expression.

### 1.3. Inflammatory Bowel Disease

Inflammatory bowel disease (IBD) is an umbrella term encompassing chronic, complex, currently incurable, and costly disorders that primarily affect the gastrointestinal tract and are characterized by an unpredictable and often progressive clinical course. The two major forms of IBD are ulcerative colitis (UC) and Crohn’s disease (CD). UC is characterized by continuous inflammatory lesions confined to the colon and limited to the mucosal layer, whereas CD may affect any part of the gastrointestinal tract and typically presents with discontinuous inflammatory lesions involving the full thickness of the intestinal wall [27].

Over recent decades, IBD has evolved into a global health challenge, with increasing incidence and prevalence observed not only in Western countries but also in newly industrialized regions undergoing rapid socioeconomic transition. This trend is evident also among pediatric populations [28,29,30]. Globally, the number of individuals living with IBD increased by 84% between 1990 and 2017, exceeding 6.8 million cases, while the annual number of IBD-related deaths rose by 67% to approximately 38,000 [31]. The highest prevalence rates continue to be reported in Europe and North America [32].

The clinical manifestations of IBD are highly heterogeneous and depend on disease subtype, location, extent, activity, and severity of inflammation. In addition to intestinal symptoms, nearly half of all patients develop extraintestinal manifestations, many of which remain underrecognized or underdiagnosed. These complications include severe and potentially life-threatening conditions such as primary sclerosing cholangitis and colitis-associated cancer. Moreover, patients with IBD are at significantly increased risk of other hepatobiliary and gastrointestinal malignancies [33,34]. Consequently, IBD has a profound impact on patients’ quality of life and imposes a substantial and steadily increasing economic burden on healthcare systems worldwide, partly owing to the growing use of advanced biological therapies [35].

The etiology and pathogenesis of IBD are complex, multifactorial, and remain incompletely understood. Disease development is thought to result from interactions among genetic susceptibility, intestinal microbiota, environmental exposures, and dysregulated immune and inflammatory responses. To date, more than 240 genetic susceptibility loci have been associated with IBD [36]. According to the currently prevailing paradigm, environmental triggers acting in genetically predisposed individuals initiate an inappropriate and persistent immune response against luminal antigens, ultimately leading to chronic intestinal inflammation [37].

### 1.4. Clinical Relevance and Therapeutic Implications of NOS2 in IBD

Accumulating evidence identifies NOS2 as a key contributor to IBD pathogenesis [4,13,38]. Its expression is markedly increased in inflamed intestinal mucosa [39,40,41] and in leukocytes from patients during disease flares [42]. Elevated epithelial NOS2 expression is accompanied by increased levels of nitrosative stress markers, particularly 3-nitrotyrosine, reflecting enhanced NO production and peroxynitrite-mediated protein nitration within the lesions [43,44,45]. These findings have stimulated interest in NOS2 as both a biomarker and therapeutic target in IBD. Although evidence remains limited, mucosal NOS2 activity correlates with clinical, endoscopic, and histological measures of disease severity in UC [46]. In turn, persistent NOS2 activity in endoscopically normal mucosa has been proposed as a potential prognostic indicator [47]. However, neither NOS2 expression nor nitrotyrosine levels have been validated for routine prediction of disease course, relapse, or therapeutic response.

The prominent role of NOS2 in intestinal inflammation initially suggested that its inhibition could represent a rational therapeutic strategy. However, translation into clinical practice has proven difficult (reviewed in [14,16]). Pharmacological inhibition or genetic ablation of NOS2 ameliorates inflammation in some experimental colitis models [19,22], whereas other studies have demonstrated impaired mucosal healing, compromised antimicrobial defense, or exacerbated disease following NOS2 suppression [21,48]. These divergent findings reflect the context-dependent functions of NOS2 and NO in host defense, immune regulation, tissue repair, and inflammatory injury [13,16]. As a result, selective NOS2 inhibition has not become an established therapy in IBD, and therapeutic development has largely shifted toward targeting upstream inflammatory pathways, including TNF-α, IL-12/23, ‘Janus kinases/signal transducer and activator of transcription’ (JAK/STAT), and integrins (reviewed in [49]). However, several approved IBD therapies indirectly modulate NOS2 activity by suppressing pathways involved in its induction. Patients treated with 5-aminosalicylates (5-ASA) exhibit lower mucosal *NOS2* transcript levels than untreated individuals [50], consistent with in vitro studies showing reduced NOS2 mRNA and protein expression in human colonic cells [51]. Anti-TNF therapy likewise decreases a major stimulus for *NOS2* induction and has been associated with reduced mucosal *NOS2* expression [50]. Azathioprine has also been shown to lower NOS2 transcript and protein levels in rodent macrophages [52]. These observations suggest that modulation of NOS2 may contribute to the efficacy of current therapies, although NOS2 itself is not considered a primary therapeutic target. Glucocorticoids may suppress *NOS2* transcription through inhibition of NF-κB- and AP-1-dependent pathways [53,54], but their effects in humans are inconsistent. While corticosteroids reduced *NOS2* expression in cultured explants of inflamed human mucosa [55], persistent NOS2 expression has been reported in biopsies from treated patients, leading to the suggestion that NOS2 may identify corticosteroid-refractory disease [56].

Interest in NOS2 as a therapeutic target extends beyond IBD to associated hepatobiliary and gastrointestinal cancers. Chronic NOS2 activation has been implicated in genomic instability and DNA damage, including single- and double-strand breaks, nitrosative deamination of DNA bases, and the formation of adducts with derivatives of endogenous N-nitrosamines [43,57], thereby contributing to the increased risk of neoplastic transformation in patients with IBD [58]. Consistent with these observations, NOS2 is overexpressed in colitis-associated colorectal cancer, sporadic colorectal cancer, and cholangiocarcinoma [59,60,61]. Moreover, a prognostic signature comprising *NOS2* and six additional IBD-related genes has been shown to enable risk stratification in patients with colorectal cancer [60].

Consequently, increased NOS2 expression has often been considered a potential therapeutic target. However, its role in carcinogenesis is also highly context dependent. Although many experimental studies suggest that NOS2-derived NO promotes tumor growth, angiogenesis, and progression, others indicate anti-neoplastic effects depending on NO concentration, duration of exposure, and cellular source [62,63]. Furthermore, reduced NOS2 expression has been associated with enhanced epithelial–mesenchymal transition and increased metastatic potential in some cancer models [64]. Accordingly, despite extensive investigation of NOS inhibitors, NO donors, and other NO-modulating compounds, no NOS2-targeted therapy has entered standard treatment protocols for colitis-associated or colorectal cancer [63].

Collectively, these findings highlight the challenges of translating mechanistic insights on NOS2 into effective therapies. Rather than simple inhibition of enzyme expression or activity, successful therapeutic modulation of NOS2 will likely require selective, context-specific approaches. A deeper understanding of the molecular mechanisms regulating NOS2 expression may facilitate the development of more precise diagnostic tools and therapeutic strategies targeting NO-dependent pathways in IBD and inflammation-associated colorectal carcinogenesis.

## 2. *NOS2* Gene Organization

**Figure 1:**
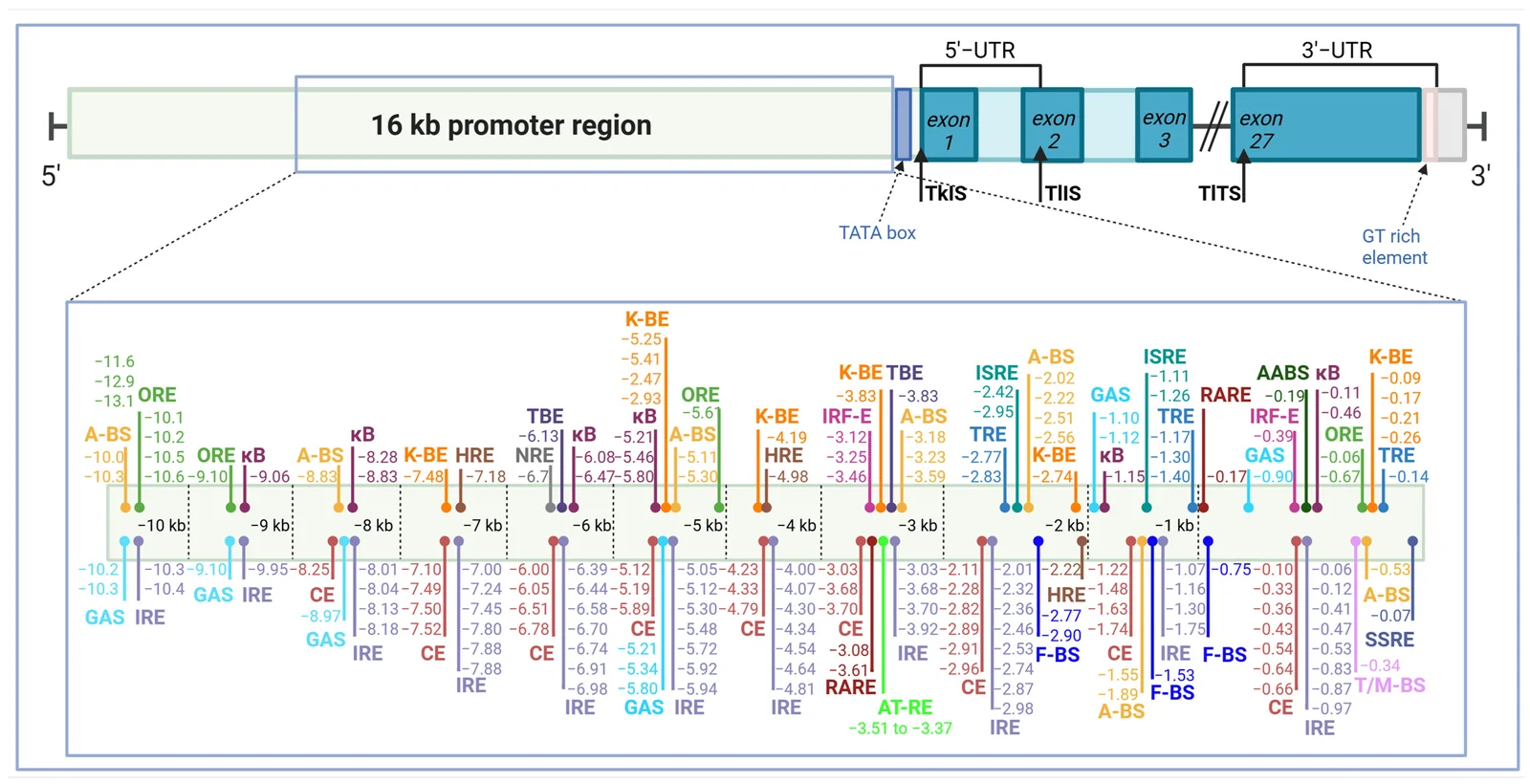
A simplified schema of NOS2 gene and promoter region organization. Created in BioRender. Krzystek-Korpacka, M. (2026) https://core.local.biorender.dev/api/shortlink/evu9u57. AABS, A activator-binding site; A-BS, activator protein (AP)-1-binding site; AT-RE, AT-rich elements; CE, the CCAAT/enhancer-binding protein (C/EBP; NF-IL6) element; F-BS, FOXO3A-binding sequence; GAS, gamma-activating factor (GAF) activation site; HRE, hypoxia response element; IRE, binding sites for interferon (IFN) γ-regulatory elements; IRF-E, IFN regulatory factor element; ISRE, IFNγ-stimulated response element; K-BE, Krüppel-like (KLF)-binding element; NRE, negative regulatory element for NRF; NRF, nuclear factor (NF) κB-repressing factor; ORE, Oct-1-response element; RARE, retinoic acid (RA) responsive element; SSRE, shear stress-response element; TBE, T-cell factor (TCF) 4-binding element; TkIS, transcriptional initiation site; TlIS, translational initiation site; TlTS, translational termination site; T/M-BS, TCF11/MafG-binding site; TRE, tumor necrosis factor (TNF) α-response element; UTR, untranslated region.

The human *NOS2* gene is located on chromosome 17q11.2 and contains 27 exons and 26 introns, although earlier studies reported 26 exons and 25 introns [65,66]. Translation initiates in exon 2 and terminates in exon 27, while binding sites for key enzymatic cofactors are distributed across exons encoding the calmodulin- (exons 12 and 13), FMN- (exon 16), FAD- (exon 19), and NAD-binding domains (exons 23, 25, and 26) [65,66]. Initial characterization of approximately 1.8 kb of the upstream promoter identified several consensus response elements, including IFNγ- and TNF-response elements as well as binding sites for NF-κB and C/EBPβ [65,67,68]. However, unlike the murine promoter, this proximal region exhibited limited responsiveness to proinflammatory stimuli such as IFNγ, TNFα, IL-1β, IL-6, and microbial signals [67]. Instead, it appeared to support constitutive *NOS2* expression in cells exposed continuously to microorganisms, including intestinal epithelial cells [69]. Subsequent studies demonstrated that the human *NOS2* regulatory region extends to at least −16 kb and contains multiple distal enhancer elements [69,70]. Notably, the transcriptional regions controlling *NOS2* induction differ substantially among species and cell types [70,71]. Whereas promoter activity between −6.2 and −5.0 kb contributes to *NOS2* induction in the liver and lung epithelium [41,72], a more distal enhancer region between −10.7 and −8.7 kb is required for maximal inducible expression in intestinal epithelial cells [70]. Bioinformatic and functional studies have identified numerous additional regulatory motifs within the extended promoter, including binding sites for hypoxia-inducible factors (HIFs), ‘nuclear factor erythroid 2-related factors’ (NRFs), activator protein 1 (AP-1), octamer transcription factor (Oct), the Krüppel-like factors (KLFs), and transcription factor 4 (TCF4), underscoring the complexity and context-dependent regulation of *NOS2* transcription [69,70,71,72,73,74,75,76,77,78,79,80,81,82,83,84]. Figure 1 presents a simplified schema of *NOS2* gene organization and the binding sites of key transcription factors within its promoter region, compiled from published reports [65,66,67,68,69,70,71,72,73,74,75,76,77,78,79,80,81,82,83,84].

## 3. Transcriptional Regulation of *NOS2* Expression

As noted above, NOS2 is regulated predominantly at the transcriptional level. Its expression is controlled by a complex network of transcription factors that integrate inflammatory, microbial, metabolic, developmental, and stress-related signals. Although numerous regulatory pathways influence *NOS2* transcription, NF-κB and IFNγ/JAK/STAT1 signaling constitute the principal drivers of inducible expression and cooperate with IRF proteins to establish a robust inflammatory response [80,85]. Most other transcription factors act by modulating the magnitude, duration, or cellular specificity of *NOS2* induction.

Positive regulators include AP-1, HIFs, KLFs, ETS family members, Oct factors, TCF4, C/EBPβ, and XBP1, whereas FOXO3, CNC-bZIP family members (TCF11/NRF1 and NRF2), and TGFβ-SMAD signaling generally limit *NOS2* expression. Importantly, the activity of many of these factors is highly context-dependent and varies according to cell type, microenvironment, and disease activity.

**Figure 2:**
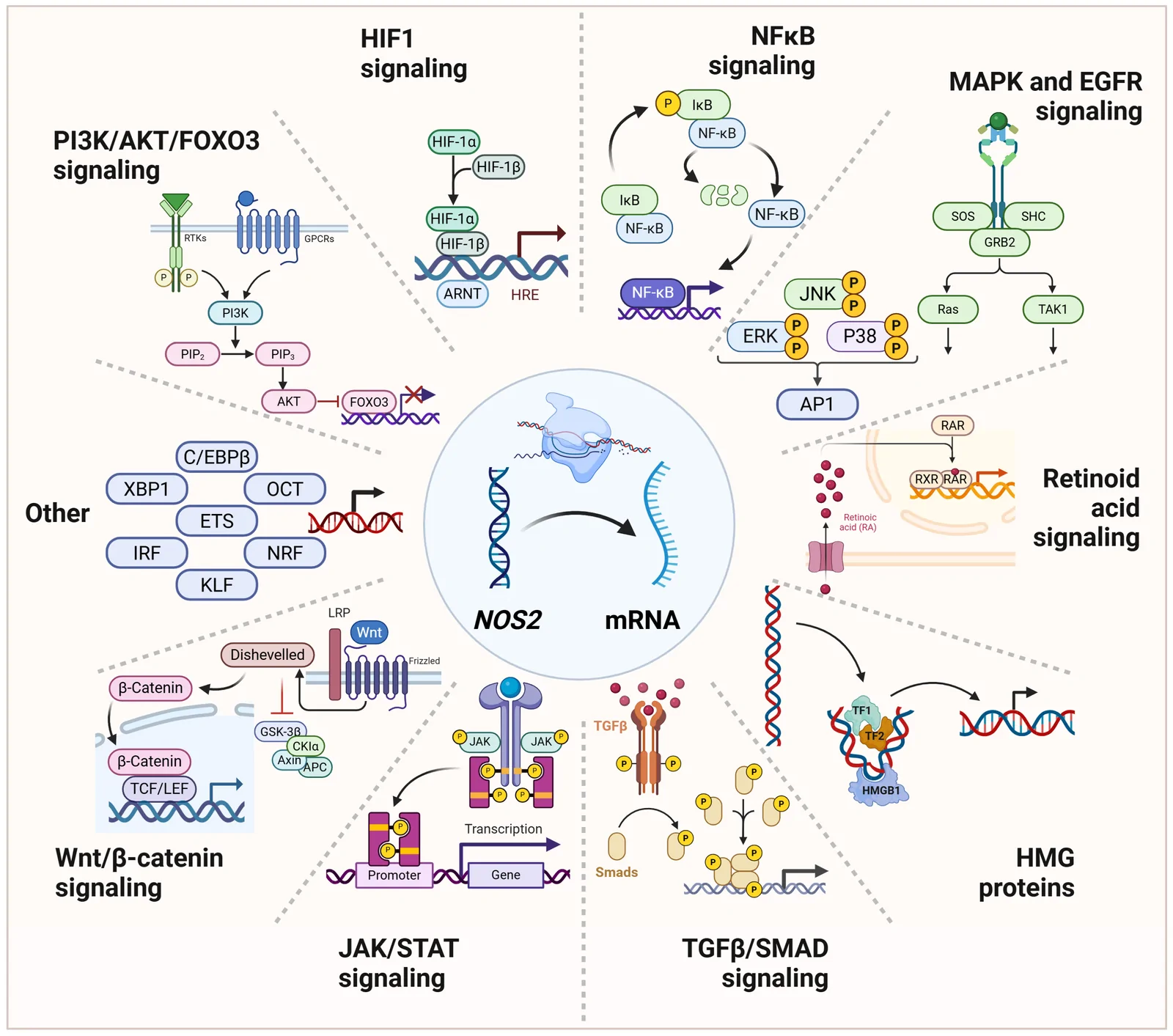
An overview of principal signaling pathways involved in NOS2 transcription. Created in BioRender. Krzystek-Korpacka, M. (2026) https://core.local.biorender.dev/api/shortlink/evu9u57. AP-1, activator protein 1; ARNT, ‘aryl hydrocarbon receptor nuclear translocator’; C/EBPβ, ‘CCAAT/enhancer-binding protein beta’; EGFR, epidermal growth factor receptor; ERK, extracellular signal-regulated kinase; ETS, ‘E26 transformation-specific’; FOXO3, ‘Forkhead box O3’; GRB2, ‘growth factor receptor-bound protein’ 2; HIF, hypoxia-inducible factor; HMG, high-mobility group; IκB, inhibitory κB; HRE, hypoxia response element; IRF, interferon regulatory factor; JAK, Janus kinase; JNK, c-Jun N-terminal kinase; KLF, Krüppel-like factor; MAPK, mitogen-activated kinase; αNOS2, inducible nitric oxide synthase; NRF, ‘nuclear factor erythroid 2-related factor’; Oct, octamer-binding transcription factor; PI3K/Akt, ‘phosphatidylinositol 3-kinase/protein kinase B’; PIP_2_, phosphatidylinositol 4,5-bisphosphate; PIP_3_, phosphatidylinositol 3,4,5-trisphosphate; RAR, retinoic acid receptor; RXR, retinoid X receptor; SHC, ‘Src homology 2 domain-containing’; SMAD, ‘suppressor of mothers against decapentaplegic homolog’; SOS, ‘Son of Sevenless’; STAT, signal transducer and activator of transcription’; TAK1, TGFβ-activated kinase 1; TCF/LEF, T-cell factor/lymphoid enhancer-binding factor; TGFβ, transforming growth factor-β; Wnt, ‘wingless-related integration site’; XBP1, ‘X-box binding protein 1’.

Evidence from human IBD and experimental models indicates that dysregulated interactions among these pathways contribute to sustained *NOS2* expression, chronic intestinal inflammation, and inflammation-associated carcinogenesis. An overview of the signaling network governing *NOS2* transcription is presented in Figure 2, and the principal transcriptional regulators, their cognate regulatory elements, activating stimuli, and relevance to IBD are summarized in Table 1.

### 3.1. NF-κB: Master Regulator of Inflammatory NOS2 Expression

NF-κB is a central transcription factor in IBD, integrating inflammatory and microbial signals and a major regulator of *NOS2* transcription [80,85,86]. It is activated by several distinct upstream pathways, rather than by a single trigger. The strongest evidence from human IBD and experimental colitis points to activation through microbial pattern-recognition receptors (Toll-like receptors (TLRs) and ‘nucleotide-binding oligomerization domain’ (NOD2)), pro-inflammatory cytokine receptors (especially for TNFα, IL-1, and IL-17), and antigen receptor signaling in immune cells [86,87,88,89]. The upstream stimuli differ markedly between cells, with innate sensors dominating in intestinal epithelial and myeloid cells and cytokine receptors and antigen receptors dominating in adaptive immune cells.

#### 3.1.1. An Overview of NF-κB Signaling

NF-κB is a family of five proteins: NF-κB1 (p105, which is processed to p50), NF-κB2 (p100, processed to p52), RelA (p65), RelB, and c-Rel. These proteins form dimeric transcriptional complexes that are typically retained in the cytoplasm. Upon activation—either via the canonical or non-canonical pathway—these dimers translocate to the nucleus, where they bind κB sequences in the promoters of target genes, including *NOS2* [90,91].

In the canonical pathway, NF-κB is sequestered in the cytoplasm by inhibitory κB (IκB) proteins. Phosphorylation of IκB, which marks it for proteasomal degradation, releases the NF-κB dimer, enabling rapid transcriptional activation without requiring new protein synthesis. This phosphorylation is mediated by the IκB kinase (IKK) complex, composed of two catalytic subunits, IKKα and IKKβ, and a regulatory subunit, IKKγ (also known as ‘NF-κB essential modulator’, NEMO) [90,91]. Upstream, IKK is phosphorylated by the MAPKK kinase, ‘transforming growth factor (TGF)-β-activated kinase 1’ (TAK1). TAK1 is induced in response to IL-1/IL-1R signaling, alarmins (‘damage-associated molecular patterns’; DAMPs), microbial signals, or TNFα/TNFR1 engagement [90,91,92]. In adaptive immune cells, NF-κB can also be activated by antigen-mediated B-cell receptor (BCR) or T-cell receptor (TCR) signaling, involving protein kinase C (PKC) [90,91].

In the non-canonical pathway, IKKα is activated by ‘NF-κB-inducing kinase’ (NIK). Activated IKKα phosphorylates p100, promoting its processing to p52, which then forms transcriptionally active dimers with RelB and translocates to the nucleus. NIK is activated in response to ligand binding to select TNFR family members, such as RANKL (‘receptor activator of NF-κB ligand’), CD40, LTβR (‘lymphotoxin β receptor’), or BAFF-R (‘B-cell activating factor receptor’). In resting cells, NIK is kept inactive in complexes with TRAFs (‘TNFR-associated factors’) and cIAPs (‘cellular inhibitors of apoptosis proteins’). Unlike the canonical pathway, non-canonical NF-κB activation is slow and persistent [90,91].

Most *NOS2* expression in IBD is likely driven by canonical NF-κB (RelA/p50) downstream of pattern-recognition receptors (TLRs and NOD2) and cytokine receptors (for IL-1 and TNF), with IFNγ/STAT1 acting as an essential cooperating pathway. In both homeostatic and inflamed intestine, the non-canonical NF-κB pathway (RelB/p52) is important for mucosal immune organization and chronic inflammatory maintenance. Although RelB-containing complexes may contribute to *NOS2* expression [80], current evidence does not support the non-canonical pathway as a direct regulator of *NOS2* transcription in the intestine. Rather, its potential contribution is mediated by sustained upregulation of *NOS2*-inducing TNFα, IL-1β, and IFNγ, characteristic of chronic inflammation [93].

#### 3.1.2. Binding Sites for NF-κB and Its Repressor

The proximal promoter (<3.8 kb) contains several putative binding sites, of which the contribution of the −0.11 kb κB site to baseline *NOS2* expression has been confirmed in endothelial and intestinal epithelial cells [68,71,94,95]. Although the core promoter is generally considered non-inducible by cytokines, this proximal κB site is involved in IL-1β-stimulated *NOS2* expression in both cell types [94,95].

However, for human intestinal *NOS2* expression in IBD, the most relevant NF-κB-responsive regulatory element currently supported by experimental evidence is the distal enhancer at −5 to −6.2 kb upstream of the *NOS2* transcription start site. It has been functionally validated by deletion and mutagenesis studies and remains the best-supported NF-κB regulatory module in the human *NOS2* gene. Within a cluster of κB elements located there, binding of p65/p50 to the −5.8 kb site is essential for promoter activation, contributing to both constitutive expression and induction by IL-1β and TNF-α co-stimulation [72]. The κB sites at −5.2 and −5.8 kb overlap with GAS motifs, and, at least in lung epithelial cells, the −5.2 kb site is activated exclusively by STAT1 (‘signal transducer and activator of transcription’), whereas the −5.8 kb site exhibits bifunctional regulation [80].

At −6.74 kb, the distal *NOS2* promoter harbors a specific negative regulatory element for NF-κB (NRE), which interacts with the NF-κB-repressing factor (NRF) to limit constitutive *NOS2* expression. Consistently, deletion of this element increases basal NOS2 levels in unstimulated cells without affecting cytokine-induced expression [82].

#### 3.1.3. Upstream Pathways Activating NF-κB

##### Microbial Signals and Pattern Recognition Receptors

Among the pathways implicated in intestinal *NOS2* induction, NF-κB signaling downstream of microbial pattern-recognition receptors, particularly TLRs and NOD2, appears to play a major role. TLRs on intestinal epithelial cells, macrophages, and dendritic cells are induced by microbial products such as lipopolysaccharide, flagellin, and peptidoglycan. Except for TLR3, signaling proceeds through MyD88, IRAKs, and TRAF6, activating the IKK complex, which degrades IκB and allows NF-κB (p50/p65) to translocate to the nucleus. Under physiological conditions, intestinal TLR signaling is tightly regulated to allow microbial sensing without excessive inflammation. TLR expression is cell-specific and generally lower in intestinal epithelial cells than in professional immune cells. Still, intestinal epithelial cells constitutively express TLR2, TLR3, TLR4, TLR5, and TLR9 (reviewed in [96,97]). During active IBD, several TLRs become dysregulated, leading to enhanced NF-κB activation and increased *NOS2* expression. TLR4, a dominant *NOS2*-inducing TLR in intestinal epithelial cells, macrophages, dendritic cells, and Paneth cells, shows the most consistent upregulation in active IBD [89,98].

NODs belong to the ‘nucleotide-binding domain and leucine-rich repeat-containing proteins’ (NLRs) family of pattern-recognition receptors. NOD1 and NOD2 are cytosolic sensors of peptidoglycan derivatives, respectively, γ-D-glutamyl-meso-diaminopimelic acid and muramyl dipeptide. Upon activation, NODs recruit RIP2 kinase, leading to ubiquitination events that activate the IKK complex and NF-κB. NOD2 is a primary *NOS2* regulator in the intestine, expressed by macrophages, dendritic cells, Paneth cells, and intestinal epithelial cells. Under homeostatic conditions, NOD2 contributes to controlled microbial sensing, antimicrobial peptide production by Paneth cells, and maintenance of host–microbiota equilibrium. In this setting, NOD2-dependent *NOS2* expression is likely limited and tightly regulated. Although NOD2 signaling alone can induce inflammatory gene expression, its major physiological role appears to be amplification and orchestration of responses to microbial stimuli (reviewed in [99,100]). In macrophages, NOD2 activation cooperates strongly with TLR signaling, resulting in enhanced NF-κB, AP-1, and STAT1 activation, all of which are critical transcriptional regulators of *NOS2* [101,102]. In murine macrophages, intracellular flagellin stimulates *NOS2* through inflammasomes NLRC4/NAIP5 and caspase-1 signaling, independently of IL-1β and IL-18 [103]. Caspase-1 facilitates NF-κB access to the *NOS2* promoter by cleavage of PARP1 (‘poly [ADP-ribose] polymerase 1’) and chromatin decondensation [104].

##### Cytokines and Their Receptors

TNFα is a central mediator of intestinal immunity that supports mucosal defense under physiological conditions. In IBD, excessive TNFα production perpetuates inflammatory signaling, disrupts epithelial barrier integrity, and drives tissue injury, providing the biological basis for the development of anti-TNF therapies (reviewed in [105]). Although putative TNF-responsive elements have been identified within the human *NOS2* regulatory region, available functional studies suggest that TNFα promotes *NOS2* expression predominantly through NF-κB activation. However, TNFα alone is generally insufficient to induce robust *NOS2* expression, particularly in humans, whose *NOS2* promoter is considerably less responsive to cytokine stimulation than its rodent counterpart. Human cells typically require the coordinated action of multiple cytokines, reflecting tighter transcriptional and epigenetic regulation of the *NOS2* gene. Consequently, *NOS2* expression in human intestinal tissues is usually dependent on synergistic stimulation by IL-1β and TNFα, which activate the canonical NF-κB pathway, and is further enhanced by IFNγ through activation of the JAK/STAT1 pathway [106]. In human intestinal cells, IL-1β generally provides the stronger initiating NF-κB signal, whereas TNF-α most often exerts its greatest effect through synergy with IL-1β and IFN-γ [69,80]. The IL-23/Th17 axis also contributes to intestinal *NOS2* upregulation, albeit mostly indirectly. IL-23 produced by activated dendritic cells and macrophages promotes the expansion and maintenance of Th17 cells, resulting in increased secretion of IL-17A, TNF-α, and other pro-inflammatory mediators [107]. IL-17 activates NF-κB- and MAPK-dependent signaling in intestinal epithelial and stromal cells and acts synergistically with IL-1β, TNF-α, and IFN-γ to enhance *NOS2* expression. Thus, whereas IL-1β directly activates NF-κB-dependent *NOS2* transcription, IL-17 and IL-23 primarily function as amplifiers of the inflammatory network that sustains *NOS2* induction during chronic intestinal inflammation [108,109]. However, IL-17 has been shown to induce *NOS2* expression in peripheral blood mononuclear cells obtained from patients with active CD and UC and cultured ex vivo [110].

##### Antigen-Recognition Receptors

Antigen-dependent activation of T lymphocytes through the T-cell receptor (TCR) contributes to intestinal *NOS2* induction, albeit indirectly. TCR signaling promotes differentiation of Th1 and Th17 cells, leading to production of IFN-γ and IL-17. These pathways amplify mucosal inflammation and enhance the abundance of *NOS2*-inducing cytokines, including IL-1β, TNF-α, IFN-γ, and IL-23, thereby facilitating NF-κB-dependent *NOS2* expression in intestinal epithelial, stromal, and myeloid cells [111]. In contrast, the contribution of B-cell receptor (BCR)-mediated activation is more complex, as B-cell responses may either enhance inflammation through antigen presentation and T-cell activation or suppress it through regulatory mechanisms [112].

NF-κB-activating pathways in IBD and their relevance for *NOS2* induction are summarized in Table 2.

#### 3.1.4. Feedback Inhibition of NF-κB by NOS2-Derived NO

NO produced by cytokine-induced NOS2 can participate in a negative feedback loop that limits inflammatory responses. One proposed mechanism involves S-nitrosylation of components of the NF-κB signaling pathway, including the p50 and p65 (RelA) subunits, resulting in reduced NF-κB DNA-binding activity and transcriptional activation. Through this mechanism, NO may contribute to autoregulatory control of NF-κB-dependent genes, including *NOS2* itself, thereby helping to restrain the magnitude and duration of inflammation [113,114].

#### 3.1.5. Translational Evidence from Human IBD

Activation of NF-κB is a hallmark of active IBD, with increased nuclear localization of p65 detected in both epithelial and lamina propria cells in UC and CD [86,115]. Inflamed mucosa also exhibits enhanced expression of TLR4 and its co-receptor MD-2, with more variable evidence for TLR2 upregulation [89,116,117,118]. In addition, several IBD susceptibility genes converge on NF-κB signaling pathways. The strongest evidence comes from NOD2, whose CD-associated variants disrupt microbial sensing and alter RIPK2-dependent NF-κB activation, thereby promoting chronic inflammation and potentially enhancing *NOS2* expression [87,119]. Variants in *TLR4* and, less consistently, *NFKB1* have also been linked to IBD susceptibility and may further influence NF-κB-driven transcriptional responses, including *NOS2* induction [120,121].

Although no approved IBD therapy directly targets NF-κB, several treatments attenuate this pathway indirectly and may consequently reduce *NOS2* expression. Corticosteroids inhibit NF-κB activation by stabilizing IκBα and preventing p65 nuclear translocation [86]. Anti-TNF agents suppress a major upstream activator of canonical NF-κB signaling and reduce expression of NF-κB-dependent inflammatory genes [122]. Likewise, therapies targeting the IL-23/Th17 axis, including ustekinumab, risankizumab, and mirikizumab, diminish production of pro-inflammatory mediators that amplify NF-κB activity [123]. Vedolizumab, although acting primarily by blocking α4β7-mediated lymphocyte trafficking, also suppresses TNF-dependent and Th17-associated inflammatory pathways in the intestinal mucosa [124]. Collectively, these observations suggest that attenuation of NF-κB-driven inflammatory programs may represent an important downstream mechanism through which multiple therapeutics limit intestinal inflammation and potentially reduce *NOS2* expression in IBD.

#### 3.1.6. Experimental Evidence

Experimental studies have firmly established NF-κB as a key regulator of intestinal inflammation. Pharmacological inhibition of NF-κB signaling, either through blockade of IKK activity or direct NF-κB decoy strategies, reduces disease severity and suppresses expression of NF-κB-dependent inflammatory mediators, including *NOS2*, in colitis models [125,126]. Likewise, genetic or pharmacological disruption of IKK-dependent NF-κB signaling attenuates inflammatory cytokine production and suppresses NF-κB-regulated transcriptional programs in experimental intestinal inflammation [127,128]. Collectively, these studies demonstrate that activation of NF-κB is not merely associated with intestinal inflammation but contributes directly to the induction and maintenance of *NOS2* expression in the inflamed gut.

#### 3.1.7. Concluding Remarks

NF-κB represents one of the central transcriptional regulators of *NOS2* expression in the intestine. Through integration of microbial, cytokine, and danger-associated signals, NF-κB coordinates both the initiation and maintenance of *NOS2* transcription. While physiological activation contributes to host defense and mucosal homeostasis, persistent NF-κB activation in IBD promotes chronic *NOS2* expression, excessive generation of reactive nitrogen species, and tissue injury. The importance of this pathway is further underscored by genetic susceptibility loci, human mucosal expression studies, and the indirect suppression of NF-κB signaling by multiple effective IBD therapies.

### 3.2. IFNγ-JAK-STAT-IRF Signaling: Defining Cytokine Responsiveness of the *NOS2* Promoter

IFNγ/STAT1 signaling represents a second major axis regulating *NOS2* transcription in the intestine, functioning through cooperation with NF-κB and other transcription factors, including IRF family members, to generate sustained *NOS2* expression [69,80].

#### 3.2.1. Overview of the IFNγ/JAK/STAT1 Pathway

Upon binding of IFNγ to its receptor, associated JAK kinases phosphorylate STAT1, enabling STAT1 dimerization and nuclear translocation. Activated STAT1 dimers bind gamma-activated sequence (GAS) elements within regulatory regions of target genes and initiate a transcriptional program involved in antimicrobial defense, immune activation, and inflammation [129]. In contrast to primary IFNγ-responsive genes, however, *NOS2* functions predominantly as a secondary-response gene and requires cooperation between STAT1 and additional transcriptional regulators for efficient transcriptional activation. In intestinal epithelial cells, STAT1 primarily cooperates with IRF1 and, alternatively, with IRF8. Accordingly, regulatory regions of IFNγ-responsive genes, including *NOS2*, contain distinct binding sites for STAT1 homodimers (GAS elements), IRF1 homodimers (IRF-E elements), and IRF1/IRF8 heterodimers (ISRE elements). These motifs facilitate higher-order chromatin interactions and confer IFNγ responsiveness. However, individual GAS, IRF-E, or ISRE motifs are generally insufficient to support full transcriptional activation, which instead requires cooperative occupancy by STAT1 and IRF factors [129].

#### 3.2.2. Cooperation with IRF1, IRF8, and NF-κB

IRF1 and IRF8 are key downstream mediators of IFNγ signaling and play complementary roles in myeloid cells. IRF8 contributes to the basal transcriptional programs that establish and maintain macrophage identity and function, whereas IRF1 is rapidly induced by IFNγ and mediates the transcriptional activation of inflammatory and antimicrobial genes in response to immune stimulation [130,131]. Consequently, efficient *NOS2* induction requires both transcription factors, and deletion of either IRF1 or IRF8 markedly reduces *NOS2* expression and NO production.

IFNγ-dependent *NOS2* regulation is also tightly integrated with NF-κB signaling. IRF1 and IRF8 can cooperate with the NF-κB p65/p50 heterodimer at regulatory regions within the *NOS2* locus [132]. This interaction contributes to a two-stage model of *NOS2* induction. During the initial phase, NF-κB rapidly induces transcription of both *IRF1* and *NOS2*, generating a transient response. In the subsequent phase, cooperative activation by NF-κB and IRF1 sustains *NOS2* transcription and promotes prolonged NO production. Notably, IRF1 is constitutively present in the nuclei of resting cells and contributes to basal transcription of host-defense genes independently of STAT1. Most IRF-E and ISRE elements are dedicated to this constitutive IRF1 activity and are not competent to recruit STAT1 for inducible transcription, highlighting the mechanistic distinction between basal and IFNγ-induced gene regulation [133].

#### 3.2.3. Negative Regulation of IFNγ Signaling

As with NF-κB signaling, IFNγ-driven responses are controlled by endogenous negative-feedback mechanisms. Activated STAT1 induces expression of SOCS proteins (‘suppressor of cytokine signaling’), particularly SOCS1, which inhibit JAK kinase activity and limit further STAT1 phosphorylation [134]. Additional regulation is provided by phosphatases and PIAS (‘protein inhibitors of activated STAT’) that promote STAT1 dephosphorylation, thereby terminating signaling [135,136].

#### 3.2.4. Translational Evidence from Human IBD

Multiple lines of evidence support a pathogenic role of the IFNγ/JAK/STAT1 axis in human IBD. The *IFNG* locus has been linked to UC susceptibility [137], and sustained IFNγ production contributes not only to IBD pathogenesis [138] but also to colitis-associated colorectal cancer, partly through induction of *NOS2* and cyclooxygenase-2 (*COX2*) expression [139]. Consistent with these observations, inflamed intestinal tissues from IBD patients overexpress both IFNγ [140] and STAT1 [141], indicating persistent activation of this signaling pathway. Recent single-cell and spatial transcriptomic studies have further strengthened this concept by identifying prominent IFNγ-response and JAK/STAT1 transcriptional signatures across inflamed epithelial, myeloid, and stromal cell populations, demonstrating that activation of this pathway extends beyond infiltrating lymphocytes and represents a tissue-wide feature of IBD. In particular, epithelial cells exhibit sustained interferon-driven programs characterized by increased antigen-presentation machinery and STAT1-associated gene expression, whereas inflammatory macrophage populations display enrichment of cytokine- and JAK/STAT-related pathways, collectively supporting a central role for IFNγ signaling within the inflamed intestinal microenvironment [142,143,144].

The translational relevance of downstream IFNγ effectors is further supported by genetic studies. Genome-wide association analyses have shown that loci associated with increased susceptibility to CD and UC are significantly enriched among genes regulated by IRF1 and IRF8, including *NOS2* [145]. Furthermore, deregulation of endogenous negative regulators of the pathway has also been implicated in disease pathogenesis. Of particular interest is *PTPN2*, which encodes the STAT1 phosphatase TC45 and functions as a key terminator of IFNγ signaling. *PTPN2* is an established IBD susceptibility gene, and loss of *PTPN*2/TC45 function enhances STAT1 activation, increases responsiveness to IFNγ, and promotes intestinal inflammation, highlighting the importance of defective signal termination in sustaining IFNγ-driven inflammatory programs [146,147].

#### 3.2.5. Experimental Evidence

Experimental studies have established the IFNγ/JAK/STAT1 pathway as a critical regulator of *NOS2* expression. Genetic disruption of STAT1 abolishes key IFNγ-dependent transcriptional responses, demonstrating the essential role of this transcription factor in interferon-mediated gene regulation and macrophage activation [148]. Likewise, mice deficient in IRF1, a major downstream effector of STAT1, exhibit profoundly impaired *NOS2* induction, producing little or no NO in response to IFNγ and lipopolysaccharide stimulation and displaying increased susceptibility to intracellular infection [149]. Further mechanistic studies demonstrated that IFNγ-dependent *NOS2* induction requires direct recruitment of IRF1 to regulatory elements within the *NOS2* promoter, establishing IRF1 as a key mediator of IFNγ-driven transcriptional activation [150]. In turn, deficiency of inhibitory SOCS1 results in exaggerated IFNγ/STAT1 signaling, contributing to both severe colitis and colorectal carcinogenesis [139]. Together, these findings identify the IFNγ/STAT1-IRF1 axis as an essential driver of *NOS2* transcription and NO production, providing a mechanistic framework through which persistent IFNγ signaling may sustain *NOS2* expression during chronic intestinal inflammation.

#### 3.2.6. Concluding Remarks

Collectively, the IFNγ/JAK/STAT1 pathway constitutes a major regulatory axis controlling *NOS2* expression in the intestine. Through coordinated interactions with IRF1, IRF8, and NF-κB, STAT1 integrates cytokine-derived signals into a transcriptional program that promotes antimicrobial defense while sustaining inflammatory responses. Persistent activation of this network in IBD, together with impaired negative regulation by molecules such as *PTPN2*/TC45 and SOCS1, provides a mechanistic link between chronic cytokine exposure, heightened *NOS2* expression, and inflammation-associated tissue damage and carcinogenesis.

### 3.3. AP-1: Integrator of Microbial, Cytokine, and Stress-Induced NOS2 Expression

#### 3.3.1. Overview of AP-1 Signaling

Activator protein-1 (AP-1) is a dimeric transcription factor composed primarily of proteins belonging to the JUN and FOS families. It functions as a key downstream effector of mitogen-activated protein kinase (MAPK) signaling. In intestinal cells, AP-1 is activated by pro-inflammatory cytokines, microbial products, and cellular stress, thereby linking environmental cues to transcriptional responses involved in host defense and inflammation. Among the major pathways converging on AP-1 are the JNK, p38, and ERK cascades, which are activated downstream of cytokine and pattern-recognition receptors [151].

#### 3.3.2. AP-1-Responsive Elements in *NOS2* Regulation

The human *NOS2* promoter contains several AP-1-responsive elements that exert context-dependent effects on transcription. An AP-1 binding site located within the core promoter represses basal *NOS2* expression [81]. In contrast, consensus AP-1 elements positioned approximately 5.1 and 5.3 kb upstream of the transcription start site act as distal enhancers and are required for *NOS2* transcription induced by cytokines and lipopolysaccharide/IFNγ [78,79]. Thus, AP-1 contributes not only to inducible *NOS2* expression but also to the fine-tuning of basal promoter activity.

#### 3.3.3. Cooperation with STAT1 and Chromatin-Remodeling Factors

Efficient AP-1-dependent *NOS2* induction requires cooperation with other transcriptional regulators. The distal enhancer region contains neighboring STAT1-binding sites that facilitate formation of STAT1/c-Fos transcriptional complexes and potentiate cytokine-induced promoter activation [152]. In addition, AP-1-mediated activation is strongly enhanced by recruitment of the transcriptional coactivator p300. Binding of p300 to the enhancer region promotes long-range chromatin looping, bringing distal regulatory elements into proximity with the *NOS2* core promoter and TATA box, thereby enabling robust inducible transcription [153].

#### 3.3.4. Integration of Microbial and Stress Signaling Pathways

AP-1 also functions as a convergence point for inflammatory and stress-responsive signaling pathways implicated in intestinal inflammation. Microbial sensing through NOD receptors activates kinases RIPK2 and TAK1, leading not only to NF-κB activation but also to stimulation of JNK, p38, and ERK pathways, which subsequently activate AP-1 [154]. In parallel, genotoxic stress, inflammatory cytokines, and endotoxins induce PARP1, which promotes JNK/AP-1 signaling and further enhances *NOS2* expression [155].

#### 3.3.5. Translational Evidence from Human IBD

Although AP-1 signaling has been less extensively characterized in IBD than NF-κB or STAT1 signaling, activation of upstream AP-1-associated MAPK pathways has been documented in inflamed intestinal mucosa. Studies of colonic biopsies from patients with CD and UC demonstrated significantly increased activation of p38 MAPK and JNK, particularly within lamina propria macrophages and other inflammatory cells, consistent with enhanced AP-1-inducing signaling in the inflamed intestine [156].

More recently, transcriptomic analyses of inflamed mucosa have identified enrichment of stress-response, cytokine-response, and MAPK-associated gene-expression programs in IBD [157]. Single-cell transcriptomic studies further revealed extensive inflammatory remodeling of epithelial and immune-cell populations, with activation of signaling pathways converging on AP-1-regulated transcriptional networks [143].

Although direct assessment of AP-1 activity in human IBD remains limited, the consistent activation of JNK/p38 MAPK signaling and enrichment of MAPK-responsive transcriptional programs support a role for AP-1 in coordinating intestinal inflammatory responses. Given its integration of cytokine-, microbial-, and stress-induced signals, persistent AP-1 activation may contribute to sustained *NOS2* expression and excessive NO production during chronic intestinal inflammation.

#### 3.3.6. Experimental Evidence

Experimental studies further support a functional role for AP-1 in intestinal inflammation. PARP1 deficiency attenuates experimental colitis, reduces JNK/AP-1 activation, and decreases *NOS2* expression and NO production, highlighting the importance of this pathway in inflammation-driven *NOS2* induction [155]. Pharmacological inhibition of AP-1 activity using decoy oligodeoxynucleotides attenuated dextran sulfate sodium (DSS)-induced colitis and reduced expression of pro-inflammatory mediators, demonstrating that AP-1 contributes directly to intestinal inflammatory responses [158].

Additional evidence linking AP-1 to chronic intestinal pathology comes from studies of colitis-associated tumorigenesis, in which c-Jun and other AP-1 family members were strongly expressed in inflamed and neoplastic intestinal tissues. These findings support persistent activation of AP-1-regulated transcriptional programs during chronic inflammation and suggest a role for AP-1 at the interface between intestinal inflammation and tumorigenesis [159].

#### 3.3.7. Concluding Remarks

Collectively, AP-1 functions as an important integrator of microbial, cytokine, and stress-induced signaling pathways regulating *NOS2* expression. Through cooperation with STAT1, p300, and MAPK signaling, AP-1 controls enhancer-dependent transcriptional activation of *NOS2* and may contribute to sustained *NOS2* expression in chronic intestinal inflammation.

### 3.4. HIFs: Mediators of Hypoxia-Dependent NOS2 Regulation

#### 3.4.1. Overview of HIF Signaling

The intestinal epithelium exists along a steep oxygen gradient, transitioning from the largely anaerobic lumen to the oxygen-rich subepithelium. This unique environment has driven the development of adaptive mechanisms that allow epithelial cells to function under conditions of relative oxygen deprivation, often referred to as “physiologic hypoxia”, which is important for maintaining epithelial barrier integrity and mucosal homeostasis [160]. During inflammation, however, disruption of barrier function and recruitment of activated immune cells markedly increase local oxygen consumption, resulting in profound tissue hypoxia. Reduced oxygen availability stabilizes the α-subunits of hypoxia-inducible factors (HIF-1α, HIF-2α and HIF-3α), enabling formation of transcriptionally active HIFα/β heterodimers that regulate hypoxia-responsive gene expression [161].

#### 3.4.2. HIF-Responsive Elements in *NOS2* Regulation

The human *NOS2* promoter contains multiple hypoxia-responsive elements (HREs), including a functional HRE located approximately 4.9 kb upstream of the transcription start site that participates in cytokine-induced *NOS2* expression in intestinal epithelial cells [76]. HIF-1α directly binds these regulatory elements and promotes *NOS2* transcription in macrophages and endothelial and intestinal epithelial cells exposed to hypoxia or inflammatory stimuli such as lipopolysaccharide and pro-inflammatory cytokines [76,160,162]. While HIF-1α is primarily associated with rapid induction of *NOS2* during acute inflammatory responses, HIF-2α appears capable of sustaining *NOS2* expression in epithelial and endothelial cells, thereby contributing to prolonged NO production [163].

#### 3.4.3. Crosstalk with Inflammatory Signaling Pathways

HIF-dependent regulation of *NOS2* is closely integrated with inflammatory signaling networks. HIF-1α cooperates with both NF-κB and STAT1 to amplify *NOS2* transcriptional responses [160,162]. The interaction between hypoxia and NF-κB signaling is particularly important in inflamed tissues. Under normoxic conditions, prolyl hydroxylases promote degradation of HIFα proteins and can also suppress NF-κB activation through oxygen-dependent hydroxylation of IKKβ. During hypoxia, inhibition of prolyl hydroxylases permits stabilization of HIF factors and enhances NF-κB activation, thereby reinforcing inflammatory gene expression, including *NOS2* [164]. Consequently, hypoxia and inflammation cooperate to generate a transcriptional environment highly permissive for sustained *NOS2* induction.

#### 3.4.4. Physiological and Pathological Consequences of HIF-Dependent *NOS2* Expression

Under physiological conditions, transient activation of HIF signaling represents an adaptive response that helps maintain epithelial barrier function and host defense. In this setting, NOS2-derived NO contributes to the regulation of local oxygen gradients, mucosal antimicrobial activity, and epithelial adaptation to reduced oxygen availability [160,161]. In contrast, chronic activation of HIF signaling during intestinal inflammation may promote excessive *NOS2* expression and prolonged NO production. The resulting increase in reactive oxygen and nitrogen species can contribute to oxidative stress, epithelial injury, and amplification of inflammatory responses, thereby linking chronic hypoxia to disease progression [76,160,161].

#### 3.4.5. Translational Evidence from Human IBD

Accumulating evidence indicates that activation of hypoxia-responsive pathways is a characteristic feature of human IBD. Both HIF-1α and HIF-2α are detectable in inflamed but not normal colonic tissue, with HIF-2α showing particularly diffuse expression in CD [165]. More recent transcriptomic, single-cell, and spatial profiling studies have identified epithelial transcriptional programs consistent with activation of hypoxia- and HIF-associated pathways in inflamed intestinal epithelium, supporting the concept that epithelial hypoxia is a fundamental component of the IBD microenvironment. Increased expression of HIF-regulated genes has been observed together with interferon- and inflammatory-response signatures, highlighting extensive crosstalk between hypoxic and immune pathways during chronic intestinal inflammation [143,166].

#### 3.4.6. Experimental Evidence

Beyond the evidence for activation of hypoxia-responsive pathways in human IBD, experimental studies have shown that pharmacological stabilization of HIF signaling improves barrier function and attenuates intestinal inflammation. These findings have stimulated growing interest in HIF-targeted therapies aimed at promoting mucosal healing and restoring epithelial homeostasis in IBD [167].

#### 3.4.7. Concluding Remarks

Collectively, these findings identify HIF signaling as a critical link between tissue hypoxia and inflammatory gene regulation in the intestine. Through direct transcriptional activation of *NOS2* and extensive cooperation with NF-κB and STAT1 pathways, HIFs integrate metabolic and inflammatory signals to regulate mucosal defense. While transient HIF activation is protective and promotes epithelial adaptation, persistent activation in chronic inflammation may drive excessive *NOS2* expression, oxidative stress, and tissue injury, thereby contributing to the pathogenesis of IBD and its complications.

### 3.5. KLF4 and KLF6: Context-Dependent Integrators of Inflammatory and Stress Signals in NOS2 Regulation

#### 3.5.1. Overview of KLF Signaling

Krüppel-like factors (KLFs) are a family of zinc-finger transcription factors that bind GC-rich DNA sequences and CACCC motifs within gene promoters and enhancers. Rather than constituting a classical signaling pathway, KLFs function as transcriptional integrators downstream of diverse stimuli, including TLR/NF-κB, IFNγ/JAK/STAT, TGFβ/SMAD, oxidative stress, and hypoxia-associated signaling pathways. Although several KLF family members contribute to intestinal inflammation [168], current evidence primarily implicates KLF4 and KLF6 in the regulation of *NOS2* expression.

KLF4 is highly expressed in differentiated intestinal epithelial cells and contributes to epithelial maturation, goblet-cell differentiation, barrier maintenance, and restriction of epithelial proliferation and migration [169]. In immune cells, however, KLF4 displays context-dependent functions. It can be induced by inflammatory stimuli such as lipopolysaccharide and cooperate with pro-inflammatory signaling pathways, but it also promotes STAT6-dependent M2 macrophage polarization and suppresses M1-associated inflammatory programs [170,171].

In contrast, KLF6 functions predominantly as an inflammation-responsive transcription factor. KLF6 expression is induced by TLR signaling, TNFα, hypoxia, oxidative stress, and tissue injury, and is associated with activation of NF-κB-dependent inflammatory programs and macrophage polarization toward a pro-inflammatory phenotype [84,172].

#### 3.5.2. KLF-Responsive Elements in *NOS2* Regulation

Both KLF4 and KLF6 directly regulate *NOS2* transcription through binding to functional KLF-responsive elements within the human *NOS2* promoter. Feinberg et al. identified two functional KLF4-binding elements located approximately at −0.095 and −0.210 kb relative to the transcription start site and demonstrated that KLF4 enhances *NOS2* promoter activity in response to IFNγ and lipopolysaccharide [170]. Mutation of these elements markedly reduced promoter inducibility, establishing KLF4 as a direct regulator of *NOS2* transcription. Mechanistically, KLF4 cooperates with the NF-κB subunit p65/RelA and enhances transcriptional activation of the *NOS2* promoter. In parallel, KLF4 antagonizes TGFβ/SMAD3-mediated repression of *NOS2* by competing with SMAD3 for the co-activator p300 [170].

Similarly, Warke et al. identified two functional CACCC motifs located at approximately −0.164 and −0.261 kb within the proximal human *NOS2* promoter and demonstrated direct binding of KLF6 using electrophoretic mobility shift and chromatin immunoprecipitation assays [84]. Mutation of either binding site markedly reduced KLF6-dependent promoter activation. Moreover, KLF6 overexpression increased endogenous NOS2 mRNA and protein expression as well as NO production, demonstrating functional regulation of the endogenous *NOS2* locus [84]. Thus, both KLF4 and KLF6 participate directly in *NOS2* transcriptional activation through discrete promoter-associated response elements.

#### 3.5.3. Integration with Inflammatory, Stress, and Polarization Pathways

Although both factors directly regulate *NOS2* transcription, they occupy distinct positions within the inflammatory network. KLF4 exhibits pronounced context dependence. During acute inflammatory activation, it acts downstream of TLR4 signaling and cooperates with NF-κB to stimulate *NOS2* transcription [170]. However, KLF4 also cooperates with STAT6 and promotes alternative macrophage activation. Macrophage-specific KLF4 deficiency enhances M1-associated inflammatory gene expression, supporting a role for KLF4 as a regulator of anti-inflammatory macrophage differentiation [171]. Consequently, KLF4 may either promote or restrain *NOS2* expression depending on whether acute inflammatory signaling or macrophage polarization predominates.

In contrast, available evidence suggests a more uniformly pro-inflammatory role for KLF6. Binding of KLF6 to the *NOS2* promoter is enhanced by cellular stressors including hypoxia, heat shock, serum deprivation, and phorbol myristate acetate (PMA)/ionophore stimulation, indicating that KLF6 links cellular stress responses to inducible NO synthesis [84]. In addition, KLF6 promotes NF-κB-dependent inflammatory gene expression and suppresses STAT3-mediated anti-inflammatory signaling, thereby facilitating M1 macrophage activation and sustained inflammatory responses [172]. Collectively, KLF4 and KLF6 integrate microbial, inflammatory, and stress-associated signals into the transcriptional machinery regulating *NOS2* expression.

#### 3.5.4. Translational Evidence from Human IBD

Evidence for involvement of KLF signaling in human IBD is strongest for KLF6. Goodman et al. [172] demonstrated significantly increased KLF6 expression in inflamed intestinal mucosa and myeloid cells isolated from CD and UC patients, with highest expression detected in actively inflamed regions. Given the established role of KLF6 in both *NOS2* transcription and inflammatory macrophage activation, these observations support a contribution of the KLF6 axis to pathological NO production in human IBD. In contrast, translational evidence regarding KLF4 remains inconsistent. Recent evidence indicates decreased KLF4 expression in mucosal biopsies from IBD patients and associates KLF4 deficiency with epithelial barrier dysfunction and enhanced inflammatory activity [173]. Conversely, other investigators observed increased local KLF4 expression in UC, where KLF4 was associated with TXNIP/NLRP3 inflammasome activation and epithelial pyroptosis [174]. These discrepant findings might reflect differences in disease severity and activity, tissue compartment, and cellular composition of the analyzed samples.

#### 3.5.5. Experimental Evidence

Experimental studies largely support a pathogenic role for KLF6 but have produced conflicting conclusions regarding KLF4. For KLF4, intestine-specific gene deletion was reported to attenuate dextran sulfate sodium (DSS)-induced colitis by suppressing NF-κB activation and reducing inflammatory injury [175]. In contrast, more recent studies found that KLF4 deficiency aggravates DSS- and 2,4,6-trinitrobenzenesulfonic acid (TNBS)-induced colitis, impairs epithelial barrier integrity, and increases epithelial apoptosis [173,176]. These apparently contradictory findings suggest that KLF4 has context-dependent functions in intestinal inflammation, acting as a pro-inflammatory regulator in some settings while preserving epithelial barrier integrity, goblet-cell differentiation, and tissue homeostasis in others.

By comparison, the experimental evidence for KLF6 is more consistent. KLF6 expression is increased during experimental colitis, whereas its deficiency reduces susceptibility to DSS-induced injury, diminishes expression of *NOS2* and other pro-inflammatory genes, and promotes anti-inflammatory mediators such as IL10 and arginase 1 [172]. Myeloid-specific deletion of *KLF6* likewise suppresses pathogenic macrophage activation and attenuates intestinal inflammation [172].

#### 3.5.6. Concluding Remarks

KLF4 and KLF6 emerge as important but mechanistically distinct regulators of intestinal *NOS2* expression. Whereas KLF4 exerts context-dependent effects that integrate inflammatory signaling, epithelial differentiation, and macrophage polarization, KLF6 functions predominantly as a pro-inflammatory activator of *NOS2* and inflammatory macrophage responses. The relative activity of these two KLF-dependent pathways may therefore represent an important determinant of NO production in both intestinal homeostasis and IBD.

### 3.6. FOXO3: Connecting Inflammatory and Metabolic Pathways in *NOS2* Regulation

#### 3.6.1. Overview of FOXO3 Signaling

FOXO3 (‘forkhead box O3’; also known as FKHRL1) belongs to the FOXO family of transcription factors, which function as central regulators of cellular differentiation, metabolism, oxidative stress responses, apoptosis, autophagy, and inflammation. FOXO3 activity is primarily controlled by the phosphatidylinositol 3-kinase (PI3K)/Akt pathway. Upon activation by growth factors, cytokines, or microbial stimuli, Akt phosphorylates FOXO3, promoting its association with 14-3-3 proteins and subsequent nuclear export. As a result, FOXO3-dependent transcription is suppressed [177,178].

In the intestinal mucosa, FOXO3 is expressed in both intestinal epithelial and immune cells, where it contributes to maintenance of epithelial homeostasis and limits inflammatory responses. Accordingly, loss of FOXO3 activity has been associated with intestinal inflammation, dysplasia, and colorectal cancer development [179,180,181].

#### 3.6.2. FOXO-Responsive Elements in *NOS2* Regulation

The human *NOS2* promoter contains several FOXO3-binding elements, of which the site located approximately 1.53 kb upstream of the transcription start site has been shown to be functionally relevant [177]. Unlike most transcription factors discussed in the context of *NOS2* regulation, FOXO3 acts primarily as a transcriptional repressor. In lung epithelial cells, FOXO3 suppresses *NOS2* promoter activity under both basal and lipopolysaccharide/IFNγ-stimulated conditions [177]. Activation of the PI3K/Akt pathway results in phosphorylation and inactivation of FOXO3, thereby relieving repression of the *NOS2* promoter. Importantly, this mechanism appears largely independent of AP-1 and NF-κB signaling, indicating that FOXO3 represents a distinct inhibitory pathway controlling *NOS2* transcription [177]. These observations identify FOXO3 as one of the few transcription factors directly repressing *NOS2* promoter activity rather than promoting it.

#### 3.6.3. Integration with Inflammatory and Metabolic Signaling Pathways

FOXO3 serves as a major convergence point for inflammatory and metabolic pathways that influence intestinal *NOS2* expression. Both lipopolysaccharide and TNFα induce PI3K/Akt signaling in human and murine intestinal epithelial cells, resulting in phosphorylation and nuclear exclusion of FOXO3 [182,183]. In addition, TNFα can suppress FOXO3 independently of Akt through activation of IKKβ, which phosphorylates FOXO3 and targets it for proteasomal degradation [179,184]. Consequently, inflammatory signaling can inhibit FOXO3 through multiple parallel mechanisms.

FOXO3 also suppresses inflammation indirectly through regulation of sirtuin-6. Activation of FOXO3 increases sirtuin-6 expression, whereas sirtuin-6 antagonizes NF-κB signaling by inducing *NFKBIA* (IκBα) expression and by deacetylating Lys^9^ in histone H3 (H3K9) at NF-κB target promoters, including *NOS2* [185,186]. Thus, the FOXO3/sirtuin-6 axis provides an additional layer of transcriptional control linking metabolic regulation to inflammatory gene expression.

A further level of complexity is provided by interactions between FOXO3 and lipid metabolism. TNFα-induced suppression of FOXO3 and sirtuin-6 promotes lipid droplet accumulation in intestinal epithelial cells. These lipid droplets subsequently drive cyclooxygenase-2/prostaglandin E_2_ (COX2/PGE_2_) signaling, which further inhibits FOXO3 activity and reinforces a feed-forward inflammatory circuit [183]. Similar reciprocal regulation between lipid storage and FOXO3 signaling has been described in macrophages and neutrophils [187,188]. Collectively, these findings position FOXO3 at the intersection of inflammatory, metabolic, and epigenetic pathways regulating *NOS2* expression.

#### 3.6.4. Translational Evidence from Human IBD

Several observations support the clinical relevance of FOXO3 in human intestinal inflammation. Reduced *FOXO3A* expression has been reported in inflamed colonic mucosa from patients with IBD and in colorectal cancer lesions compared with adjacent uninvolved tissue [181]. Loss of *FOXO3* expression is accompanied by transcriptional signatures associated with enhanced inflammatory activity and increased NO synthesis [181]. Additional evidence is provided by genetic studies. A functional *FOXO3A* polymorphism (rs12212067; T → G) has been associated with a milder clinical course of Crohn’s disease [180]. Following TLR4 stimulation, carriers of the protective G allele exhibit increased *FOXO3A* expression, more rapid restoration of nuclear FOXO3 activity, enhanced TGFβ1 production, and reduced secretion of pro-inflammatory cytokines [180]. These findings support a role for FOXO3 as an endogenous brake on intestinal inflammation.

#### 3.6.5. Experimental Evidence

Experimental studies consistently identify FOXO3 as a protective factor in intestinal inflammation. *FOXO3A*-deficient mice develop spontaneous intestinal inflammation and display increased susceptibility to dextran sulfate sodium (DSS)-induced colitis [179,180]. Moreover, transcriptomic analyses of *FOXO3A*-deficient intestinal tissue reveal enrichment of inflammatory pathways, including NO biosynthesis-related programs, resembling changes observed in inflammatory and dysplastic lesions [181].

Experimental suppression of FOXO3 activity through inflammatory cytokines, PI3K/Akt signaling, or dietary interventions similarly promotes intestinal inflammation. High-fat diets reduce *FOXO3A* expression in both intestinal epithelial cells and colonic macrophages, while concomitantly enhancing inflammatory signaling and tumor-promoting pathways [187]. Loss of FOXO3 signaling also facilitates development of colitis-associated cancer, highlighting the importance of this pathway in linking chronic inflammation with tumorigenesis [181,187,188]. Moreover, consistent with its tumor-suppressive role, FOXO3 restrains epidermal growth factor receptor (EGFR)-driven proliferation of colon cancer cells through the FOXO3/sirtuin-6 pathway. Conversely, loss of FOXO3 promotes metabolic reprogramming characterized by increased lipid droplet accumulation and enhanced proliferative capacity [189].

#### 3.6.6. Concluding Remarks

FOXO3 functions as an important counter-regulatory determinant of intestinal *NOS2* expression. By directly repressing *NOS2* transcription and indirectly limiting NF-κB activity through the FOXO3/sirtuin-6 axis, FOXO3 restrains inflammatory NO production. Accordingly, loss of FOXO3 signaling is associated with enhanced intestinal inflammation, increased susceptibility to colitis, and promotion of colitis-associated tumorigenesis.

### 3.7. Retinoic Acid: Context-Dependent Modulator of NOS2 Expression and Intestinal Inflammation

#### 3.7.1. Overview of Retinoic Acid Signaling

Retinoic acid (RA), the active metabolite of vitamin A, regulates transcription through RAR/RXR heterodimers that bind retinoic acid response elements (RAREs) in target genes. Beyond classical RAR signaling, RXR also forms heterodimers with PPARs (‘peroxisome proliferator-activated receptors’), linking retinoic acid signaling to metabolic and inflammatory pathways. In the gut, retinoic acid regulates epithelial homeostasis and immune-cell differentiation, although its role in intestinal inflammation remains controversial [190].

#### 3.7.2. Retinoic Acid Response Elements in *NOS2* Regulation

Zou et al. identified a functional RARα/RXRα-responsive element in the human *NOS2* promoter and demonstrated direct induction of *NOS2* transcription by all-trans and 9-cis retinoic acid [191]. Mutation of this element abolished promoter responsiveness, establishing *NOS2* as a direct transcriptional target of retinoid signaling [191]. In addition, a functional PPAR response element (PPRE) has been identified in the murine *NOS2* promoter, suggesting potential regulation through PPAR/RXR complexes [192].

#### 3.7.3. Integration with Inflammatory Signaling Pathways

The influence of retinoic acid on *NOS2* expression is highly context-dependent. Whereas retinoic acid stimulates *NOS2* transcription in several epithelial and tumor cell models [191], it suppresses inducible *NOS* expression in macrophages, keratinocytes, and vascular smooth muscle cells [193,194,195]. Differential engagement of RAR/RXR and PPAR/RXR pathways has been proposed as one explanation for these opposing effects [190,192].

#### 3.7.4. Translational Evidence from Human IBD

Although retinoic acid is traditionally viewed as an anti-inflammatory mediator due to its ability to promote regulatory T-cell differentiation [196], recent studies suggest a pro-inflammatory role in IBD. Intestinal macrophages from CD patients exhibit increased retinoic acid production that promotes acquisition of an inflammatory phenotype [197], while elevated mucosal retinoic acid concentrations in UC correlate positively with IFNγ and IL-17 expression and negatively with IL-10 expression [198].

#### 3.7.5. Experimental Evidence

Experimental studies support both anti-inflammatory and pro-inflammatory effects of retinoic acid. Under homeostatic conditions, retinoic acid promotes FOXP3^+^ regulatory T-cell differentiation and suppresses Th17 development [196]. However, under inflammatory conditions, retinoid acid-conditioned dendritic cells enhance Th1 and Th17 responses [199], suggesting that the overall impact of retinoic acid signaling depends on the immunological context.

#### 3.7.6. Concluding Remarks

Retinoic acid represents one of the most context-dependent regulators of *NOS2* expression. While direct RARα/RXRα signaling activates the human *NOS2* promoter, retinoic acid may also suppress inducible *NOS* expression in inflammatory cells. Similarly, retinoic acid contributes both to immune tolerance and to amplification of intestinal inflammation, indicating that its effect on *NOS2* is determined by receptor usage, cellular context, and disease activity.

### 3.8. ETS Factors: Balancing Pro- and Anti-Inflammatory Control of NOS2 Expression

#### 3.8.1. Overview of ETS Signaling

The ‘E26 transformation-specific’ (ETS) family comprises 28 transcription factors in humans and plays an important role in the development and activation of innate and adaptive immune cells [200]. Several ETS family members have been implicated in *NOS2* regulation, including ELF3, ETS2, ELK3, and ELF4, which exert either positive or negative effects on inflammatory gene expression.

#### 3.8.2. ETS-Dependent Regulation of *NOS2* Expression

Among ETS family members, ELF3 is the best-characterized regulator of *NOS2*. Although predominantly expressed in epithelial cells, ELF3 can also be induced by inflammatory stimuli in macrophages, endothelial cells, and smooth muscle cells [201]. ELF3 contains both a canonical ETS DNA-binding domain and an AT-hook motif and stimulates *NOS2* expression through direct promoter activation as well as cooperation with NF-κB. In particular, ELF3 interacts with the NF-κB subunit p50 and enhances p65/p50-mediated transcriptional activation of the *NOS2* promoter [201]. ELF3-dependent induction of *NOS2* and *COX2* has been implicated in endothelial injury and vascular inflammation [202].

Additional ETS family members also participate in *NOS2* regulation. ETS2 enhances both basal and lipopolysaccharide-induced *NOS2* expression in murine macrophages [203], whereas ELK3 acts as a transcriptional repressor of *NOS2* and mediates part of the inhibitory effect of TGFβ1 [203]. In contrast to ELF3 and ETS2, ELF4 suppresses *NOS2* expression while inducing arginase-1, thereby promoting an anti-inflammatory M2 macrophage phenotype [204].

#### 3.8.3. Translational and Experimental Evidence in Intestinal Inflammation

Recent studies suggest that ETS factors contribute to intestinal inflammation through regulation of macrophage activation states. ETS2 has been proposed as a master regulator of inflammatory responses in human macrophages, and its overexpression induces a transcriptional program closely resembling that observed in macrophages isolated from inflamed IBD mucosa [205].

Evidence for a protective role of ELF4 is supported by both experimental and human observations. ELF4 expression is reduced in experimental colitis models, whereas inflammatory cytokine expression is increased [204,206]. Furthermore, patients with the rare syndrome termed deficiency in ELF4, X-linked (DEX) develop recurrent gastrointestinal ulceration and IBD-like manifestations [206,207]. Together with its ability to repress *NOS2* and promote arginase-1 expression, these findings suggest that ELF4 contributes to maintenance of intestinal immune homeostasis [204,206,207].

#### 3.8.4. Concluding Remarks

ETS family members exert both stimulatory and inhibitory effects on intestinal *NOS2* expression. Whereas ELF3 and ETS2 promote *NOS2* induction and inflammatory activation, ELK3 and ELF4 counteract these responses and favor anti-inflammatory pathways. Thus, regulation of *NOS2* by ETS factors appears to depend on the balance between distinct ETS family members rather than a single ETS-driven signaling axis.

### 3.9. HMG Proteins: Architectural Regulators of NOS2 Transcription

#### 3.9.1. Overview of HMG Proteins

High-mobility group (HMG) proteins constitute a family of architectural transcription factors that regulate gene expression by modifying DNA conformation rather than acting as classical sequence-specific transcriptional activators. A defining feature of HMG proteins is the presence of AT-hook motifs that bind AT-rich regions of DNA and facilitate interactions between transcription factors, co-regulators, and the basal transcriptional machinery [208]. Through these architectural functions, HMG proteins influence assembly and stability of transcriptional complexes controlling inflammatory gene expression.

#### 3.9.2. HMG Proteins in *NOS2* Regulation

Both HMGB1 and HMGA1 have been implicated in transcriptional regulation of *NOS2*. HMGB1 promotes assembly of higher-order transcriptional complexes containing NF-κB and Oct-1 at the *NOS2* promoter [209]. In addition, HMGB1 modulates NF-κB activity in a dimer-specific manner by enhancing transcription driven by p65/p50 and p50/p50 complexes while destabilizing p65/p65-DNA interactions [210]. This observation is of particular relevance to *NOS2* because the p65/p50 heterodimer represents the principal inducer of *NOS2* transcription, although alternative NF-κB dimers may contribute to cell-specific regulation [78,211,212].

HMGA1 (HMG-I/Y) directly facilitates formation of an enhanceosome containing NF-κB and IRF1 and promotes recruitment of this complex to the *NOS2* promoter [213]. Functional HMGA1-binding sites are located within AT-rich regions approximately 3.5–3.8 kb upstream of the TATA box. Disruption of HMGA1 DNA-binding activity abolishes *NOS2* promoter activity in both resting and cytokine-stimulated epithelial cells, indicating that HMGA1 is required for efficient constitutive as well as inducible *NOS2* transcription [214].

#### 3.9.3. Evidence from Human Intestinal Inflammation

Among HMG family members, HMGB1 has been linked to human IBD. *HMGB1* was included among the original gene-expression signatures distinguishing peripheral blood mononuclear cells from patients with IBD and healthy controls [215]. Given its ability to enhance NF-κB-dependent transcriptional responses and facilitate assembly of *NOS2*-regulatory complexes, HMGB1 may contribute to inflammatory NO production in intestinal inflammation.

#### 3.9.4. Concluding Remarks

HMG proteins act as architectural regulators of *NOS2* transcription by facilitating assembly and stability of NF-κB-, IRF1-, and Oct-1-containing transcriptional complexes. Whereas HMGB1 modulates NF-κB-dependent promoter activation, HMGA1 is required for efficient enhanceosome formation at the *NOS2* promoter. Thus, HMG proteins contribute to *NOS2* regulation primarily by shaping chromatin architecture and transcriptional complex organization rather than by functioning as conventional sequence-specific activators.

### 3.10. OCT Factors: Facilitators of Cytokine-Induced NOS2 Transcription

#### 3.10.1. Overview of OCT Factors

Octamer-binding transcription factors (OCTs) belong to the POU-domain family of transcription factors and regulate gene expression through binding to octamer response elements (OREs) within target promoters. Among the OCT proteins, Oct-1 has emerged as an important regulator of inducible *NOS2* expression, which does not primarily control basal promoter activity but rather modulates cytokine-induced transcriptional responses [73,216,217].

#### 3.10.2. OCT-Responsive Elements in *NOS2* Regulation

The human *NOS2* promoter contains a functional ORE located approximately 10.2 kb upstream of the transcription start site, together with several additional ORE-like sequences carrying single nucleotide mismatches [73]. Functional analyses demonstrated that Oct-1 enhances cytokine-induced *NOS2* promoter activity, resulting in increased NOS2 mRNA and protein expression in multiple human cell lines, including the intestinal epithelial cell lines DLD-1 and HCT-116. Mutation of the distal −10.2 kb ORE reduces cytokine-induced *NOS2* promoter activity by approximately 40%, establishing this element as an important contributor to inducible *NOS2* transcription [73].

The effect of OCT factors on *NOS2* expression depends on dimer composition. Whereas the Oct-1/Oct-1 homodimer promotes *NOS2* induction, formation of Oct-1/Oct-2 heterodimers suppresses *NOS2* transcription in epithelial cells, including colonic epithelium [216].

More recently, an additional functional Oct-1-binding site located in close proximity to the TATA box (−0.057 to −0.064 kb) was identified [217]. In contrast to the distal enhancer-associated ORE, this proximal site is constitutively occupied by Oct-1 in unstimulated intestinal epithelial cells. Although insufficient to initiate transcription on its own, its occupancy is required for efficient activation of the *NOS2* promoter by cytokine-responsive factors such as NF-κB, suggesting that Oct-1 contributes to the establishment of a transcriptionally competent promoter architecture prior to inflammatory stimulation [217].

Evidence from other inflammatory genes further highlights the importance of Oct-1-NF-κB cooperation. A functional *TNFA* promoter polymorphism associated with susceptibility to CD and UC affects Oct-1 binding and disrupts Oct-1 interaction with the NF-κB subunit p65, resulting in altered *TNFA* transcription [218]. Although this mechanism was not examined at the *NOS2* promoter, it supports a broader role of Oct-1-dependent transcriptional complexes in regulating inflammatory responses relevant to IBD.

#### 3.10.3. Experimental Evidence

Direct evidence linking OCT factors to intestinal inflammation remains limited. However, conditional deletion of Oct-1 (*Pou2f1*) in murine intestinal stem cells impaired recovery from dextran sulfate sodium (DSS)-induced epithelial injury despite having little effect on steady-state intestinal homeostasis. Oct-1 deficiency also reduced the capacity of intestinal organoids to regenerate after passage, indicating a role for Oct-1 in epithelial stress responses and mucosal repair [219].

#### 3.10.4. Concluding Remarks

OCT proteins contribute to *NOS2* regulation through both distal enhancer-dependent and proximal promoter-dependent mechanisms. Oct-1 generally functions as a positive regulator of cytokine-induced *NOS2* expression, whereas Oct-1/Oct-2 heterodimers exert inhibitory effects. Beyond *NOS2*, emerging evidence linking Oct-1 to *TNFA* regulation and epithelial regeneration suggests a broader role in transcriptional programs relevant to intestinal inflammation and repair.

### 3.11. TCF4: Linking Wnt Signaling to NOS2 Expression and Epithelial Defense

#### 3.11.1. Overview of TCF4 Signaling

T-cell factor 4 (TCF4) is a major downstream effector of the canonical Wnt/β-catenin pathway and plays a central role in intestinal epithelial homeostasis. In the gut, TCF4 regulates stem-cell maintenance, antimicrobial peptide production, and differentiation of secretory epithelial lineages, particularly Paneth and goblet cells [220]. Upon activation of the Wnt pathway, β-catenin accumulates in the nucleus and associates with TCF4 to induce transcription of Wnt-responsive genes.

#### 3.11.2. TCF4-Responsive Elements in *NOS2* Regulation

Direct regulation of *NOS2* by Wnt signaling was demonstrated by Du et al. [74], who identified two functional TCF4-binding elements within the human *NOS2* promoter. Mutation of either element reduced both basal and cytokine-induced promoter activity, whereas simultaneous mutation of both sites produced an even greater inhibitory effect. Binding of β-catenin/TCF4 complexes to both promoter elements was confirmed by electrophoretic mobility shift assays, establishing *NOS2* as a direct target of Wnt/β-catenin signaling [74].

Overexpression of either β-catenin or TCF4 increased *NOS2* promoter activity, *NOS2* mRNA abundance, and protein expression in HCT116 and DLD1 cells [74]. Although the stimulatory effect of β-catenin/TCF4 on *NOS2* promoter activity was relatively modest when combined with a strong cytokine cocktail, it became substantially more pronounced in the presence of weaker stimuli such as IFNγ alone [74]. Consistent with these findings, pharmacological stabilization of β-catenin using lithium chloride as well as stimulation with Wnt3A increased *NOS2* expression and NO production, further supporting a functional role of Wnt signaling in *NOS2* regulation [74].

#### 3.11.3. Translational Evidence from Human IBD

Although direct evidence connecting TCF4-dependent *NOS2* regulation with IBD pathogenesis is currently lacking, Wehkamp et al. demonstrated significantly reduced TCF4 expression and activity in ileal CD, accompanied by decreased expression of the Paneth-cell α-defensins HD5 and HD6 [221]. Notably, reduced TCF4 expression was observed irrespective of disease activity and NOD2 genotype, suggesting that impaired Wnt/TCF4 signaling represents an independent pathogenic mechanism. The functional significance of these observations was supported by studies in mice with *Tcf4* haploinsufficiecy, in which partial loss of TCF4 resulted in reduced α-defensin production and diminished antibacterial activity of Paneth cells [221].

#### 3.11.4. Concluding Remarks

TCF4 acts as a positive regulator of *NOS2* expression through direct interaction of β-catenin/TCF4 complexes with functional TCF4-binding elements within the *NOS2* promoter. Beyond its role in *NOS2* regulation, TCF4 is a critical determinant of intestinal epithelial defense and Paneth-cell function, and reduced TCF4 activity represents a recognized feature of ileal CD.

### 3.12. C/EBPβ and XBP1: Integrators of Inflammatory and Endoplasmic Reticulum Stress Signaling in NOS2 Regulation

#### 3.12.1. Overview of C/EBPβ and XBP1 Signaling

C/EBPβ (formerly NF-IL6) belongs to the ‘CCAAT/enhancer-binding protein’ (C/EBP) family of bZIP transcription factors that integrate inflammatory, metabolic, and stress-related signals. Its transcriptional activity is highly context-dependent and determined by the expression of distinct isoforms. Whereas the LAP* and LAP isoforms function as transcriptional activators, the shorter LIP isoform lacks transactivation domains and acts as a dominant-negative regulator. In addition, C/EBPβ activity is heavily influenced by post-translational modifications and interactions with other transcription factors, including NF-κB. The LAP* isoform contains a Rel-interacting domain that promotes cooperation with NF-κB, while C/EBPβ can further enhance NF-κB signaling through inhibition of IκBα [222,223].

XBP1 (‘X-box-binding protein 1’) is a central effector of the unfolded protein response. Endoplasmic reticulum (ER) stress triggers unconventional splicing of XBP1 mRNA, generating the transcriptionally active isoform XBP1s from the inactive precursor XBP1u. XBP1 is particularly important in highly secretory intestinal epithelial cells and plays an essential role in maintaining intestinal homeostasis [224].

#### 3.12.2. C/EBPβ/XBP1-Responsive Elements in *NOS2* Regulation

The human *NOS2* promoter contains multiple NF-IL6-responsive elements. Among these, the ‘A activator-binding site’ (AABS) located approximately 0.192 kb upstream of the transcription start site serves as a functional binding site for both C/EBPβ and XBP1 [83,224]. In hepatic cells, C/EBPβ binding to the AABS contributes substantially to *NOS2* transcription, whereas in intestinal epithelial cells (DLD-1) the site has little influence on basal *NOS2* expression. However, mutation of the AABS markedly reduces responsiveness of the *NOS2* promoter to cytokine stimulation, indicating that C/EBPβ participates primarily in inducible rather than constitutive *NOS2* expression [83].

The same regulatory element also binds XBP1. Under conditions of ER stress, accumulation of the active spliced isoform XBP1s promotes *NOS2* transcription, whereas predominance of the unspliced isoform is associated with reduced promoter activity [224]. These findings identify the AABS as a point of convergence between inflammatory signaling and the unfolded protein response.

#### 3.12.3. Translational Evidence from Human IBD

The strongest translational evidence concerns XBP1. Kaser et al. [225] identified genetic associations between XBP1 variants and susceptibility to both CD and UC and further demonstrated the presence of rare hypomorphic XBP1 variants in patients with IBD. These findings established XBP1 as one of the first ER stress-response genes directly implicated in IBD pathogenesis [225].

Evidence for C/EBPβ involvement is more indirect. Transcriptomic analyses of newly diagnosed patients with CD and UC identified *CEBPB* among the transcription factors most consistently associated with disease-related inflammatory transcriptional programs, supporting a role for C/EBPβ in human intestinal inflammation [226].

More recently, activation of the IRE1-XBP1 pathway has been demonstrated in intestinal ILC3s from inflamed IBD tissues, and the frequency of XBP1s^+^ ILC3s in CD patients correlated with responsiveness to anti-IL-23 therapy, suggesting potential utility of XBP1-related pathways as biomarkers of therapeutic response [227].

#### 3.12.4. Experimental Evidence

Experimental studies demonstrated that C/EBPβ promotes mucosal inflammation in both murine intestinal epithelial cells and human Caco-2 cells and contributes to the pathogenesis of experimental colitis [228,229].

The role of XBP1 has been explored extensively in vivo. Kaser et al. [225] showed that its intestinal epithelial cell-specific deletion causes spontaneous enteritis, Paneth-cell dysfunction, and increased susceptibility to experimental colitis. XBP1-deficient epithelium also exhibited exaggerated responses to bacterial products and TNFα, establishing a causal link between impaired unfolded protein response signaling and intestinal inflammation.

Consistent with these observations, genetic disruption of IRE1α-XBP1 signaling in murine ILC3s impaired cytokine production and increased susceptibility to experimental colitis, further supporting a protective role of this pathway in intestinal homeostasis [227].

#### 3.12.5. Concluding Remarks

C/EBPβ and XBP1 regulate *NOS2* through a shared promoter element that integrates inflammatory and ER stress signaling. While C/EBPβ enhances cytokine-induced *NOS2* expression in cooperation with NF-κB, XBP1 links activation of the unfolded protein response to *NOS2* transcription. The strong genetic, translational, and experimental evidence connecting XBP1 to IBD further highlights the importance of ER stress pathways in the regulation of intestinal inflammation and NO production.

### 3.13. CNC-bZIP Factors: Linking Redox Homeostasis to Repression of NOS2 Expression

#### 3.13.1. Overview of CNC-bZIP Signaling

The ‘Cap’n’Collar basic leucine zipper’ (CNC-bZIP) family of transcription factors comprises NRF1 (also known as TCF11/NFE2L1), NRF2 (NFE2L2), and NRF3 (NFE2L3), which function as key regulators of cellular adaptation to oxidative, electrophilic, and inflammatory stress [230]. Members of this family form heterodimers with small Maf proteins (MafG, MafK, and MafF) and bind NF-E2, Maf recognition, or antioxidant response elements located within regulatory regions of target genes [230]. Through regulation of antioxidant defenses, cellular metabolism, and inflammatory signaling, CNC-bZIP proteins contribute to maintenance of redox homeostasis and cytoprotection.

Among CNC-bZIP family members, TCF11/NRF1 and NRF2 have been implicated in transcriptional repression of *NOS2*. Although they act through distinct cis-regulatory elements, both factors limit *NOS2* expression as part of broader cellular mechanisms aimed at constraining excessive oxidative and nitrosative stress [75,231,232].

#### 3.13.2. CNC-bZIP-Responsive Elements in *NOS2* Regulation

Berg et al. [75] identified a functional NF-E2 recognition element within the human *NOS2* promoter that binds TCF11/MafG heterodimers and mediates transcriptional repression of *NOS2*. The activity of this repressor complex is regulated by TGFβ signaling. Specifically, TGFβ1 acting in concert with SMAD6 enhances TCF11/MafG-dependent repression of *NOS2*, thereby contributing to the inhibitory effect of TGFβ1 on *NOS2* expression, whereas SMAD7 antagonizes this pathway and abrogates TGFβ1-mediated repression [75].

Repression of *NOS2* by CNC-bZIP transcription factors is not restricted to TCF11/NRF1. Recent studies demonstrated that NRF2 suppresses *NOS2* expression through a distinct mechanism involving antioxidant response elements located within a distal enhancer of the *NOS2* locus [231]. NRF2 recruitment to this enhancer is associated with epigenetic repression of *NOS2* transcription and reduced enhancer activity. Functional studies in *KRAS*-driven pancreatic cancer cells showed that NRF2 binding limits *NOS2* expression and thereby restrains nitrosative stress despite the presence of strong oncogenic and oxidative stimuli [231]. Subsequent work further established NOS2 as part of a broader NRF2-dependent metabolic adaptation program, in which NRF2 regulates the balance between oxidative and nitrosative stress responses [232].

Beyond direct *NOS2* regulation, NRF2 has emerged as an important negative regulator of inflammatory gene expression. In macrophages, NRF2 activation suppresses pro-inflammatory transcriptional programs and limits recruitment of RNA polymerase II to inflammatory genes, providing a mechanistic framework for its broader anti-inflammatory effects [233].

#### 3.13.3. Concluding Remarks

Members of the CNC-bZIP family function predominantly as repressors of *NOS2* expression. Whereas TCF11/NRF1 mediates TGFβ-dependent repression through a promoter-associated NF-E2 element, NRF2 inhibits *NOS2* through distal enhancer-associated antioxidant response elements and epigenetic mechanisms. Together, these pathways link redox sensing, cytoprotective responses, and anti-inflammatory signaling to the control of inducible NO production.

### 3.14. TGFβ-SMAD Signaling: Preserving Endogenous Control of NOS2 Expression

#### 3.14.1. Overview of TGFβ/SMAD Signaling

Transforming growth factor-β (TGFβ) is a pleiotropic cytokine with potent immunoregulatory and anti-inflammatory activities. Upon binding to its receptors, TGFβ induces phosphorylation of SMAD2 and SMAD3, which associate with SMAD4 and translocate to the nucleus to regulate transcription of target genes. This signaling pathway is negatively regulated by SMAD7, an inhibitory SMAD that interferes with receptor-mediated activation of SMAD2/3 [234,235,236].

In the intestine, TGFβ is a major regulator of immune tolerance and mucosal homeostasis. Impaired TGFβ signaling has been linked to chronic intestinal inflammation, highlighting the importance of this pathway in maintaining physiological control of mucosal immune responses [235,236].

#### 3.14.2. TGFβ-Dependent Regulation of *NOS2* Expression

TGFβ is one of the most potent endogenous repressors of *NOS2* expression [237]. As discussed in the previous section, TGFβ suppresses *NOS2* transcription in part through induction of TCF11/MafG-dependent repression at a functional NF-E2 element within the *NOS2* promoter [75]. This mechanism is enhanced by SMAD6 and antagonized by SMAD7 [75]. In addition, TGFβ limits *NOS2* expression through broader suppression of inflammatory signaling pathways involved in *NOS2* induction, including transcriptional programs depending on NF-κB, STAT1, and C/EBPβ. Through these complementary mechanisms, TGFβ restrains inflammatory NO production and contributes to resolution of mucosal inflammation.

#### 3.14.3. Translational Evidence from Human IBD

The role of defective TGFβ signaling in human IBD was established by Monteleone et al. [238], who demonstrated marked overexpression of SMAD7 in the intestinal mucosa of CD and UC patients. Increased SMAD7 prevented efficient TGFβ signaling despite abundant local production of TGFβ1. Antisense-mediated blockade of *SMAD7* restored TGFβ signaling in IBD mucosal samples, indicating that excessive SMAD7 expression contributes directly to persistent intestinal inflammation.

More recently, Zorzi et al. [239] showed that SMAD7 overexpression is already detectable in the neoterminal ileum of CD patients during early postoperative recurrence, even before the appearance of overt endoscopic lesions. SMAD7 levels correlated with mucosal IFNγ production, supporting the concept that defective TGFβ signaling is an early pathogenic event in disease reactivation.

The translational relevance of the TGFβ-SMAD7 pathway is further supported by therapeutic studies targeting SMAD7. In a phase II trial, the oral *SMAD7* antisense oligonucleotide—mongersen—induced significantly higher rates of clinical remission in patients with active CD, providing proof-of-concept that restoration of endogenous TGFβ signaling may be therapeutically beneficial [240]. Although a subsequent phase III trial failed to confirm efficacy, later analyses suggested that differences in the biological activity of drug batches may have contributed to the discrepant outcomes, leaving the therapeutic potential of SMAD7 inhibition unresolved [241].

#### 3.14.4. Experimental Evidence

Experimental studies similarly support a protective role for TGFβ signaling in intestinal inflammation. Boirivant et al. [242] demonstrated that administration of TGFβ1 ameliorates experimental colitis and suppresses mucosal inflammatory responses. Conversely, Nakao et al. [243] showed that transgenic mice overexpressing *SMAD7* in T cells develop severe intestinal inflammation owing to impaired responsiveness to TGFβ-mediated immunoregulation. Together, these studies established defective TGFβ/SMAD signaling as a causal mechanism promoting chronic intestinal inflammation. Given the ability of TGFβ to suppress *NOS2* expression through both direct promoter-associated and indirect anti-inflammatory mechanisms, disruption of this pathway may contribute to sustained *NOS2* induction during chronic intestinal inflammation.

#### 3.14.5. Concluding Remarks

TGFβ/SMAD signaling constitutes a major endogenous mechanism limiting *NOS2* expression and inflammatory NO production. In IBD, overexpression of SMAD7 impairs TGFβ signaling, thereby weakening an important anti-inflammatory brake on *NOS2*-inducing pathways. Consequently, loss of effective TGFβ/SMAD signaling may contribute to persistent mucosal inflammation and chronic activation of NOS2-dependent responses.

## 4. Epigenetic Regulation

*NOS2* expression in IBD is tightly controlled by the interplay of histone modifications, DNA methylation, chromatin remodelers, environmental metabolites, and feedback mechanisms involving NOS2 itself. Dysregulation of these epigenetic processes contributes to both chronic inflammation and colitis-associated cancer, underscoring the therapeutic potential of epigenetic interventions in IBD.

For transcription factors to access promoters and enhancers and induce gene expression, chromatin must adopt an open conformation. In contrast, condensed, or closed, chromatin restricts binding of transcription factors and represses gene transcription. Such epigenetic silencing contributes to the relative hyporesponsiveness of the human *NOS2* promoter to inflammatory stimuli in macrophages, endothelial cells, and vascular smooth muscle cells [244,245].

Chromatin accessibility is regulated by histone post-translational modifications, including acetylation, methylation, phosphorylation, SUMOylation, ubiquitination, and poly(ADP-ribosyl)ation. The functional consequences of a given modification depend on the histone type, the modified amino acid residue, the degree of modification, co-occurring histone marks, and the cellular context. Trimethylation of Lys^9^ and Lys^27^ in histone H3 (H3K9me3 and H3K27me3) is generally associated with transcriptional repression, whereas mono- and dimethylation of Lys^4^ in H3 (H3K4me1 and H3K4me2) marks active enhancers, and H3K4me2/3 marks active promoters [246,247]. Histone acetylation at H3K9 and H3K27 (H3K9ac and H3K27ac) is likewise a hallmark of transcriptionally active chromatin.

### 4.1. Acetylation and Methylation in NOS2 Expression

As discussed in Section 3.13, NRF2 represses *NOS2* expression through a distal enhancer-associated mechanism [231,232]. Recent studies identified two antioxidant response elements within a 0.269-kb enhancer fragment located approximately 22 kb downstream of the *NOS2* transcription start site in fetal fibroblasts and HepG2 cells. Unlike the active enhancers located at −5 and −7 kb, this enhancer displays a poised chromatin signature characterized by the absence of H3K27ac and the presence of H3K4me1. Consistent with this profile, NRF2 suppresses *NOS2* transcription by reducing enhancer activity and preventing acetylation of H3K27 and H3K9 [231,232].

DNA methylation further contributes to maintenance of a closed chromatin state. Although the *NOS2* promoter lacks classical CpG islands, both proximal and distal CpG motifs have been identified, and their methylation represses *NOS2* expression. Accordingly, human primary alveolar macrophages and THP-1 monocytes exhibit extensive methylation at these sites and display only limited *NOS2* induction and NO production in response to stimulation with lipopolysaccharide and IFNγ [245].

Similarly, endothelial cells are characteristically hyporesponsive to inflammatory stimuli because of extensive H3K9 methylation, including di- and trimethylated forms associated with transcriptional repression [244,248]. This modification is mediated, at least in part, by the histone methyltransferase EZH2 (‘enhancer of zeste homolog 2’) [248]. In addition to repressive histone marks, approximately 75% of CpG dinucleotides within region I and the TATA box of the *NOS2* promoter are methylated in human umbilical vein endothelial cells (HUVECs) as well as in dermal and lung microvascular endothelial cells [244].

By comparing multiple human cell lines, Chan et al. [244] demonstrated an inverse exponential relationship between *NOS2* inducibility and promoter methylation status, in contrast to the binary on-off pattern of methylation-dependent regulation typically observed for tumor suppressor genes. Importantly, these authors further showed that reversible methylation of the proximal *NOS2* promoter participates in cytokine-dependent regulation of *NOS2* expression in intestinal epithelial cells [244].

Consistent with the repressive role of histone methylation, EZH2 expression is decreased in inflamed mucosa from patients with UC and CD, as well as in experimental colitis models. Moreover, *EZH2* silencing in Caco-2 cells enhances the expression of inflammatory mediators, including NOS2 [249].

### 4.2. Chromatin Regulators

Chromatin regulators further modulate *NOS2* expression. Among them, PARP1, a founding member of the ‘poly(ADP-ribose) polymerase’ family, has been shown to influence *NOS2* transcription. In macrophages responding to intracellular flagellin, PARP1 is cleaved by caspases downstream of the NLRC4 inflammasome, resulting in altered chromatin accessibility at NF-κB-binding sites within the *NOS2* promoter. Although PARP1 cleavage is not required for NF-κB activation per se, caspase-1/PARP1, and to a lesser extent caspase-7/PARP1, enhance NF-κB-dependent *NOS2* expression [104]. Consistent with this finding, macrophages from mice expressing non-cleavable PARP1 exhibit reduced *NOS2* expression, diminished NO and nitrotyrosine production, and impaired NF-κB transcriptional activity. Accordingly, these animals are protected from lipopolysaccharide-induced endotoxemia and intestinal ischemia–reperfusion injury [250]. In colon cancer, *PARP1* and *NOS2* transcript levels are positively correlated, and NOS2-derived NO contributes to the stemness-promoting activity of PARP1 in p53 wild-type tumors [251].

Environmental factors, including dietary components and microbial metabolites, also shape the epigenetic regulation of *NOS2* expression. Short-chain fatty acids (SCFAs), particularly butyrate, suppress *NOS2* expression in intestinal macrophages by inhibiting histone deacetylases (HDACs) and increasing H3K9 acetylation. This modification promotes recruitment of the Mi-2/NuRD repressor complex to the *NOS2* promoter, thereby attenuating expression of secondary-response genes [252]. Dysregulated HDAC activity has also been implicated in colorectal carcinogenesis. In murine models of colitis-associated cancer, inhibition of HDAC1-5 by aspirin restores global H3K27 acetylation and suppresses tumor growth. Notably, experimental cancer (azoxymethane/DSS model) is characterized by marked enrichment of H3K27ac at specific loci, including the *NOS2* promoter, resulting in more than 50-fold upregulation of *NOS2* expression; this effect is prevented by aspirin-mediated HDAC inhibition [253]. Similarly, in *Leishmania amazonensis*-infected macrophages, recruitment of inhibitory NF-κB p50/p50 homodimers to the *NOS2* promoter facilitates HDAC1 binding, thereby preventing CBP/p300-mediated H3K9 acetylation and transcriptional activation [212].

**Figure 3:**
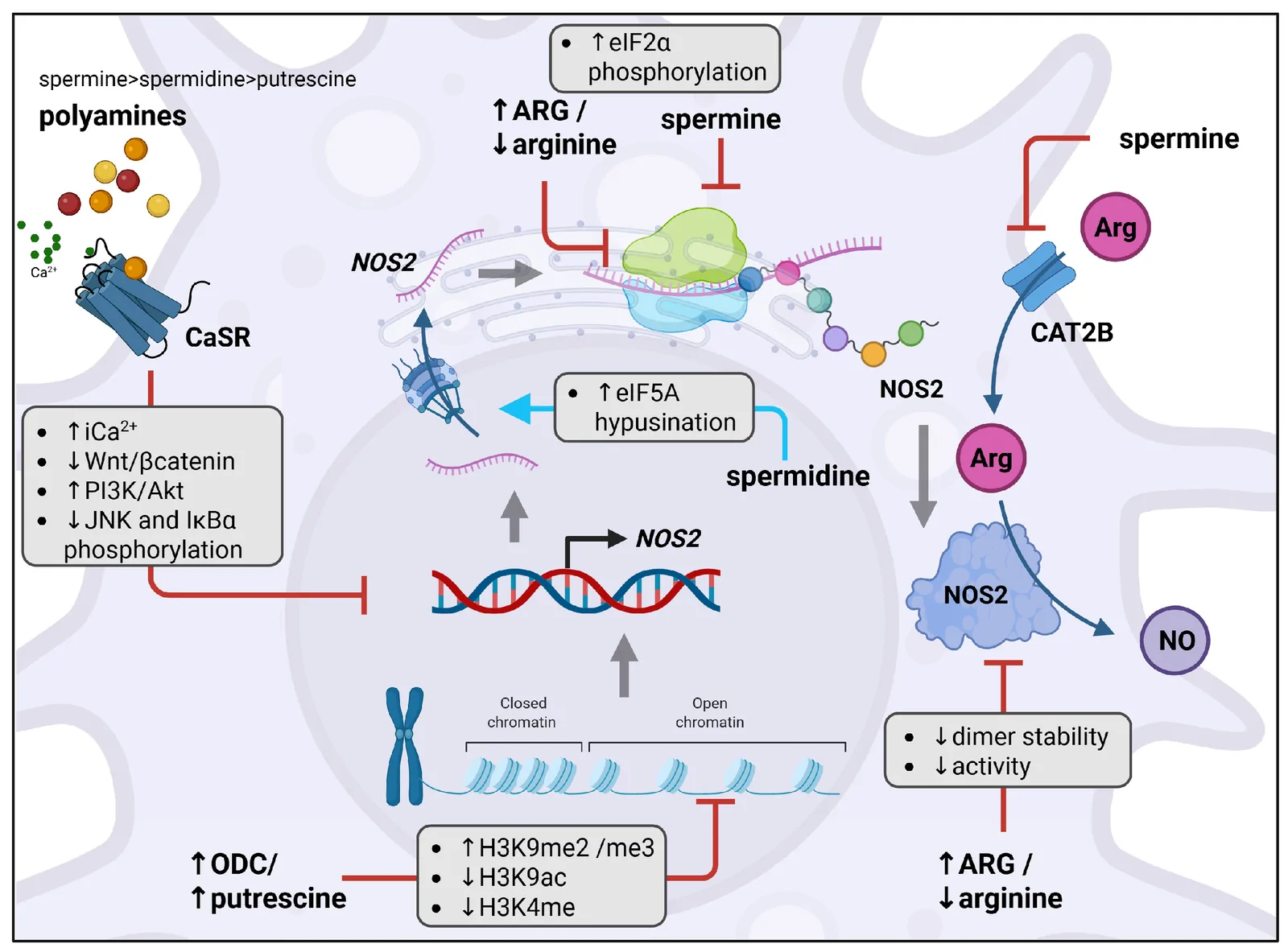
Impact of Arg/ARG pathway metabolites on *NOS2* expression and translation. Created in BioRender. Krzystek-Korpacka, M. (2026) https://core.local.biorender.dev/api/shortlink/evu9u57. ARG, arginase; Arg, arginine; CaSR, Ca^2+^-sensing receptor; CAT2B, arginine transporter (SLC7A2); eIF, eukaryotic initiation factor; H3K4 or 9me, histones methylated on lysine residues 4 or 9; iCa^2+^, intracellular calcium ions; IκBα, inhibitory κBα; JNK, ‘c-Jun N-terminal kinase’; NO, nitric oxide; NOS2, inducible nitric oxide synthase; ODC, ornithine decarboxylase; PI3K/Akt, ‘phosphoinositide 3-kinase/protein kinase B’; Wnt, ‘wingless-related integration site’.

Metabolites generated by competing arginine-utilizing pathways can also regulate *NOS2* expression through epigenetic mechanisms. Mice lacking ornithine decarboxylase (ODC) in myeloid cells exhibit exaggerated *NOS2* induction in the gastric and intestinal mucosa following enteric bacterial infection. Likewise, *ODC* deficiency enhances *NOS2* expression in macrophages stimulated with microbial products or IFNγ. Mechanistically, ODC-derived putrescine promotes H3K4 di- and trimethylation together with H3K9 deacetylation, thereby restricting *NOS2* transcription. In its absence, transcriptionally permissive marks, including H3K9 acetylation and H3K4 monomethylation, accumulate at the *NOS2* locus [254]. Interestingly, the putrescine-derived polyamines spermidine and spermine also regulate *NOS2* expression at post-transcriptional levels by influencing *NOS2* mRNA transport [255] and translation [256], as summarized in Figure 3 and discussed in Section 5.3.

NOS2 itself can participate in epigenetic regulation during transflammation, a process whereby inflammatory stimuli induce epigenetic remodeling to facilitate tissue repair. Following activation of pattern-recognition receptors, NF-κB- and IRF-dependent signaling induces *NOS2* expression, and a fraction of the protein translocates to the nucleus. There, NOS2 interacts with repressive chromatin-remodeling complexes, including NuRD and RING1A-containing ‘Polycomb Repressive Complex 1’ (PRC1). Through S-nitrosylation of these complexes, NOS2 prevents their association with chromatin, leading to loss of repressive histone marks (H3K27me3 and H3K9me3) and accumulation of activating marks (H3K4me2/3, H3K9ac, and H3K27ac) at target loci [257]. Notably, RING1A deficiency exacerbates colitis in murine models, highlighting the context-dependent consequences of NOS2-mediated epigenetic regulation [258].

### 4.3. Implications for IBD and Intestinal Inflammation

Accumulating evidence indicates that epigenetic mechanisms are important determinants of *NOS2* expression in the intestinal mucosa. Altered DNA methylation, histone modifications, and chromatin-remodeling pathways can enhance transcriptional accessibility of the *NOS2* locus and amplify inflammatory signaling. Several epigenetic regulators discussed above, including EZH2, HDACs, and NRF2-associated enhancer mechanisms, are dysregulated in IBD or colitis-associated cancer [231,232,249,253]. In addition, microbial metabolites and NOS2-derived NO can modify chromatin structure, creating feedback mechanisms that may sustain inflammation. Together, these findings suggest that epigenetic dysregulation contributes to persistent *NOS2* expression during chronic intestinal inflammation and represents a potential therapeutic target in IBD.

## 5. Post-Transcriptional Regulation of *NOS2* Expression

**Figure 4:**
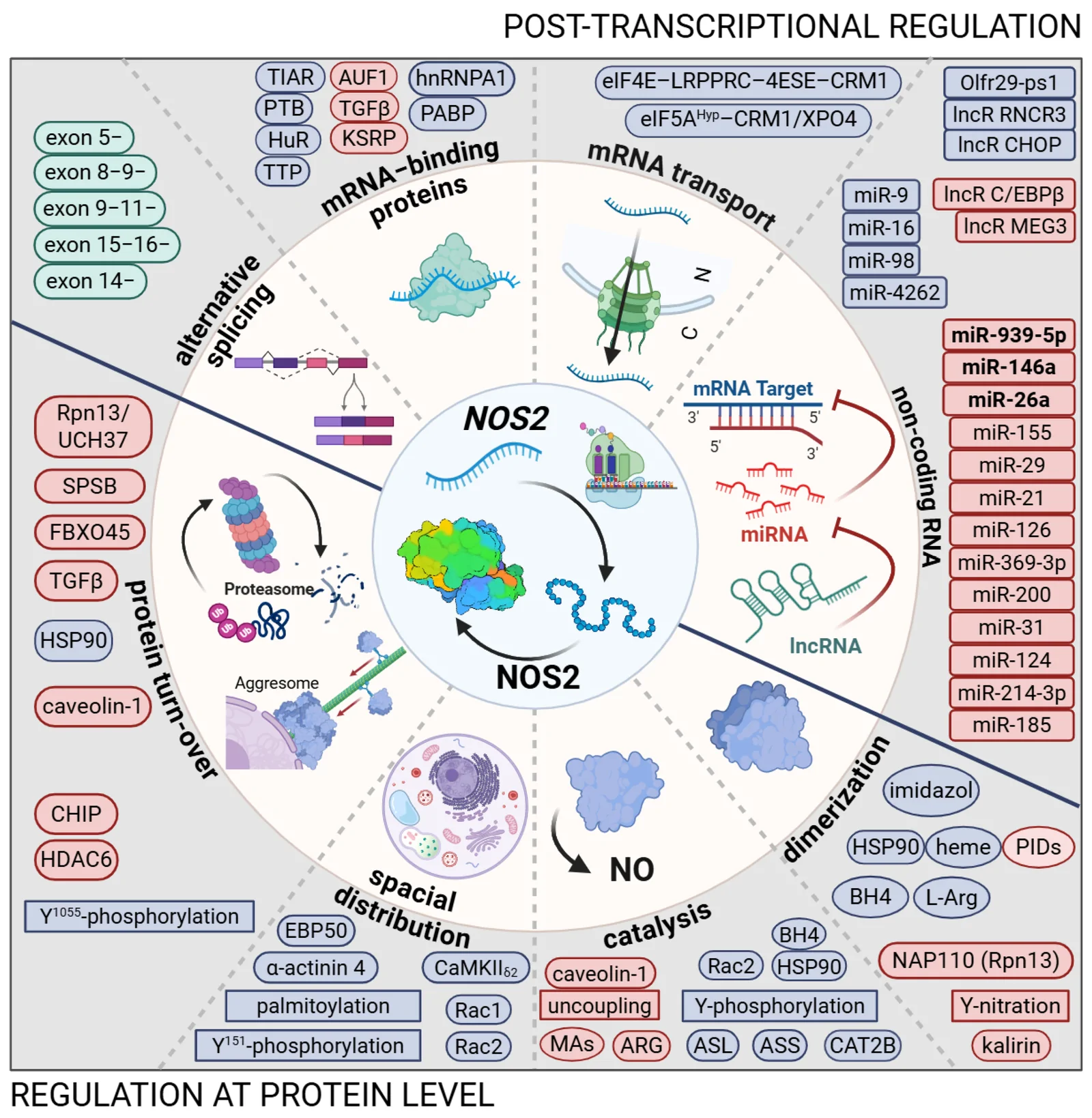
Post-transcriptional regulation of *NOS2* expression and enzyme activity. Created in BioRender. Krzystek-Korpacka, M. (2026) https://core.local.biorender.dev/api/shortlink/evu9u57. ARG, arginase; ASL, argininosuccinate lyase; ASS, argininosuccinate synthase; BH4, tetrahydrobiopterin; CaMKII, calcium/calmodulin-dependent protein kinase II; CAT2B, cationic amino acid transporter 2B; CHIP, ‘carboxyl-terminus of Hsc70-interacting protein’; CRM1/XPO, exportin; EBP50, ‘ezrin-radixin-moesin-binding phosphoprotein 50’; eIF4E, eukaryotic translation initiation factor 4E; eIF5A^Hyp^, hypusinated eukaryotic translation initiation factor 5A; 4ESE, ‘eIF4E sensitivity element’; FBXO45, ‘F-box/SPRY domain-containing protein 1’; HDAC, histone deacetylase; HSP90, heat shock protein 90 kDa; HUR, human antigen R; L-Arg, L-arginine; lncR, long non-conding RNA; LRPPRC, ‘leucine-rich pentatricopeptide repeat-containing protein; MAs, methylarginines; miR, microRNA; NAP110, ‘NOS-associated protein of 110 kDa’; NO, nitric oxide; NOS2, inducible nitric oxide synthase; PIDs, pyrimidine imidazole derivatives; PTB, ‘polypyrimidine tract-binding protein’; Rac, GTPase; Rpn13/UCH37, ‘ubiquitin receptor subunit of the 19S regulatory particle in the 26S proteasome’/deubiquitinase complex; SPSB, ‘SPRY domain- and SOCS box-containing protein’; TIAR, ‘T-cell intracellular antigen-1-related protein’; TGFβ, ‘transforming growth factor β’; TTP, tristetraprolin.

Contrary to earlier assumptions, *NOS2* expression is subject to extensive post-transcriptional regulation, although the scope and underlying mechanisms are still being elucidated. Processes regulating *NOS2* expression beyond transcription include alternative splicing, mRNA processing, stability and transport, modulation by non-coding RNAs, as well as variation in translation initiation and translational control. An overview of these mechanisms is presented in Figure 4.

### 5.1. Alternative Splicing

*NOS2* pre-mRNA undergoes alternative splicing, generating a diverse repertoire of transcript variants [259]. Early studies identified several exon-skipping events involving exons 5, 8–9, 9–11, and 16–17 (originally designated exons 15–16) [260], giving rise to transcript variants S1, S2, S3, and S4, respectively [261]. Variant S1 contains a frameshift-induced premature termination codon and therefore encodes a truncated polypeptide. In contrast, the deletions present in variants S2-S4 preserve the reading frame. The deletions in S2 and S3 do not directly affect cofactor-binding domains, whereas the deletion in S4 overlaps with the flavin mononucleotide (FMN)-binding site [260].

Nevertheless, exons 8 and 9 encode amino acid residues that are essential for NOS2 oxidase activity and dimer formation. Consequently, variants S2 and S3 are unable to form functional dimers or synthesize NO, although S2 retains reductase activity [262]. Eissa et al. [263] identified four conserved amino acid residues within exons 8 and 9, Trp^260^, Asn^261^, Tyr^267^, and Asp^280^, that are responsible for the loss of enzymatic activity in the S2 variant. All except Asp^280^ were also required for efficient NOS2 dimerization.

Alternative splicing of *NOS2* is tissue-specific and dynamically regulated by inflammatory stimuli. Exposure to cytokines or lipopolysaccharide increases expression of both the full-length transcript and alternatively spliced variants, suggesting that splicing contributes to fine-tuning of NO production during immune responses [260]. It has been proposed that truncated or monomeric variants may regulate NOS2 activity through heterodimerization with full-length NOS2 monomers, analogous to mechanisms previously described for NOS3 [264].

Distinct cell types exhibit characteristic repertoires of *NOS2* splice variants, indicating that alternative splicing contributes to cell-specific functional outcomes. In DLD-1 intestinal epithelial cells, alternatively spliced transcripts were virtually undetectable under basal conditions but were markedly induced following cytokine stimulation, particularly the S2 and S3 variants [260]. Similarly, alveolar macrophages exhibited little to no expression of alternatively spliced *NOS2* transcripts, with the notable exception of the S2 variant [260].

Emerging evidence suggests a functional role for *NOS2* alternative splicing in colorectal cancer. Comparative analyses of the isogenic colorectal adenocarcinoma cell lines SW480 and SW620, derived from a primary tumor and a lymph node metastasis, respectively, demonstrated expression of both S2 and S3 variants, with substantially higher levels in SW480 cells. Functional studies linked the predominant S3 variant to sustained proliferation and survival of SW480 cells under nutrient-restricted conditions. By limiting excessive NO production, S3 protected tumor cells from nitrosative stress and autophagy, supporting the concept that alternatively spliced *NOS2* variants function as endogenous negative regulators that fine-tune NO synthesis [261].

Additional splice variants lacking exon 14, which partially encodes the FMN-binding domain, have been identified in normal B lymphocytes and chronic lymphocytic leukemia cells [265]. Moreover, distinct NOS2 isoforms are expressed during stem cell differentiation, further indicating developmental regulation of *NOS2* splicing [66].

### 5.2. mRNA Processing and Stability

In addition to the transcription termination signal, exon 27 contains four nuclear matrix attachment regions (MARs), which enable regulation of gene expression by nuclear matrix-associated proteins. Although the canonical polyadenylation signal (AATAAA) is absent from the human *NOS2* gene, its 3′ untranslated region (UTR) contains a GT-rich sequence that functions as a minimal polyadenylation signal [266].

Despite being regulated primarily at the transcriptional level, *NOS2* expression is transient even in the continued presence of inducing stimuli, indicating an important contribution of post-transcriptional regulatory mechanisms [71]. Consistent with this notion, the 3′-UTR contains five AU-rich elements that promote mRNA degradation. Thus, although promoter activity may persist, *NOS2* transcripts remain unstable in the absence of cytokine-mediated stabilization. These elements facilitate recruitment of exosome-associated nucleases, enabling rapid degradation of *NOS2* mRNA when elevated NO production is no longer required [267]. Interestingly, inhibition of translation prolongs *NOS2* mRNA half-life [268].

Regulation of transcript stability is mediated by several RNA-binding proteins. Among them, KSRP (‘KH-type splicing regulatory protein’) and AUF1 (‘AU-binding factor 1’; hnRNP D) promote *NOS2* mRNA degradation, whereas HuR (‘human antigen R’) exerts the opposite effect by stabilizing the transcript [269]. KSRP and HuR compete for binding to the two terminal AU-rich elements within the 3′-UTR. Cytokine-induced stabilization of *NOS2* mRNA is associated with enhanced HuR binding and displacement of KSRP, a process facilitated by the zinc-finger protein tristetraprolin (TTP), which sequesters KSRP and thereby indirectly promotes transcript stabilization [270]. In this context, TTP acts as a positive regulator of *NOS2* expression [270], in contrast to its well-established role in destabilizing cyclooxygenase-2 and TNFα mRNAs through recruitment of mRNA degradation machinery [271]. However, the role of TTP appears to be species- and cell type-dependent. While Fechir et al. [272] and Linker et al. [270] found no evidence for direct TTP binding to *NOS2* mRNA in human DLD-1 cells, studies in murine colitis models demonstrated that TTP can bind the *NOS2* 3′-UTR and suppress *NOS2* expression, as TTP deficiency specific to intestinal epithelial cells resulted in increased NOS2 levels [273].

Additional RNA-binding proteins involved in *NOS2* mRNA stabilization include PTB (‘polypyrimidine tract-binding protein’), PABP (‘poly(A)-binding protein’), and TIAR (‘T-cell intracellular antigen-1-related protein’) [274,275,276]. PABP-binding sites have been identified in both the 5′- and 3′-UTRs of *NOS2* mRNA, including a site within exon 2 and two sites at the 3′ end. However, in human intestinal epithelial cells (DLD-1), a stabilizing effect has been demonstrated only for PABP binding to the 5′-UTR [276].

### 5.3. Nuclear Export of NOS2 mRNA

Nuclear export of *NOS2* mRNA involves the eukaryotic translation initiation factors eIF4E and eIF5A, both of which possess functions that extend beyond their canonical roles in translation and include chaperoning specific mRNAs from the nucleus to the cytoplasm [277,278,279,280].

eIF4E is a 7-methylguanosine (m^7^G) cap-binding protein that initiates assembly of the eIF4F complex and promotes recruitment of the 43S preinitiation complex to mRNA during translation initiation (reviewed in [280]). In the nucleus, however, eIF4E binds a subset of spliced transcripts that contain a 50-nucleotide eIF4E-sensitivity element (4ESE) within their 3′-UTR. This interaction is mediated by LRPPRC (‘leucine-rich pentatricopeptide repeat-containing protein’) and enables formation of an export complex recognized by the nuclear export receptor CRM1 (exportin 1; XPO1) [281]. Through this mechanism, eIF4E facilitates nuclear export of selected transcripts, including *NOS2* mRNA [277], thereby increasing their cytoplasmic abundance and probability of translation.

Although originally classified as a translation initiation factor, eIF5A is now recognized primarily as a regulator of translation elongation and termination (reviewed in [278]). eIF5A is unique in containing hypusine, a rare amino acid generated post-translationally by transfer of a 4-aminobutyl moiety from spermidine to the ε-amino group of Lys^50^, followed by hydroxylation [278]. Macrophage polarization toward the M1 phenotype is associated with increased hypusination, whereas inhibition of this process suppresses M1 activation [282]. Consistent with these observations, hypusinated eIF5A (eIF5A^Hyp^) is required for NOS2-dependent protection against enteric pathogens [255] and mediates nuclear export of *NOS2* mRNA in murine macrophages [255] and T cells [283]. Inhibition of deoxyhypusine synthase (DHPS), and consequently of eIF5A hypusination, blocks transport of *NOS2* mRNA to the cytoplasm. While CRM1-dependent export has been implicated in pancreatic β cells [284], other studies suggest that exportin 4 (XPO4) mediates eIF5A^Hyp^-dependent nuclear export [285].

The eIF5A pathway has also been linked to intestinal inflammation and colorectal cancer. *EIF5A* expression is elevated in colorectal tumors and has been associated with poorer patient survival [286]. Moreover, *EIF5A* has been identified as one of three blood transcriptomic markers capable of discriminating IBD patients with high accuracy [181,287]. Interestingly, Gobert et al. [288,289] reported reduced DHPS and eIF5A^Hyp^ expression in intestinal epithelial cells but increased expression in lamina propria cells from patients with IBD. Targeted deletion of *DHPS* in intestinal epithelial cells rendered mice susceptible to intestinal inflammation and neoplasia, highlighting the importance of eIF5A hypusination in maintenance of mucosal homeostasis [288]. In contrast, myeloid cell-specific *DHPS* deletion had no effect on the severity or course of experimental colitis or colitis-associated cancer, indicating cell type-specific functions of this pathway in intestinal inflammation [289].

### 5.4. Non-Coding RNA in NOS2 Regulation

A growing body of evidence implicates non-coding RNAs (ncRNAs) in the pathogenesis, progression, and complications of IBD [290,291]. Importantly, their expression patterns differ from those of healthy individuals not only in inflamed but also in quiescent mucosa, suggesting a role in disease development rather than merely reflecting ongoing inflammation [292]. ncRNAs regulate gene expression through diverse mechanisms. MicroRNAs (miRNAs; miR) act primarily at the post-transcriptional level by targeting mRNAs for degradation or translational repression, whereas long non-coding RNAs (lncRNAs) influence gene expression through multiple mechanisms, including epigenetic regulation, modulation of transcription factor activity, and sequestration of miRNAs (“sponging”), thereby preventing miRNA interaction with target transcripts [293].

*NOS2* expression is also subject to ncRNA-mediated regulation through direct interactions with *NOS2* transcripts or indirectly via modulation of pathways involved in *NOS2* induction. According to miRTargetLink 2.0 [294], miR-26a-5p is the only miRNA with strong evidence for direct targeting of *NOS2*, whereas MiRTarBase [295] additionally lists miR-155-5p and miR-939-5p as experimentally validated regulators. Beyond these miRNAs, a number of ncRNAs implicated in IBD influence pathways known to control *NOS2* expression and are therefore discussed in this section.

Because miRNA research has been particularly affected by the publication of unreliable or fraudulent studies [296], the literature considered in this review was carefully screened, and studies that had been retracted or were subject to an expression of concern were excluded. For orientation, miRNAs (Table 3) and lncRNAs/pseudogene-derived transcripts (Table 4), together with their proposed mechanisms of action and relevance to IBD, are summarized before the detailed discussion below.

#### 5.4.1. MiR-26

Compared with normal tissue, miR-26a is upregulated in both non-inflamed and inflamed mucosa from patients with UC [41] and CD [292], as well as in actively inflamed lesions from CD patients [292]. Mechanistically, miR-26a has been shown to attenuate inflammatory responses by suppressing NF-κB-responsive genes (reviewed in [297]). Although this effect was initially attributed to targeting the architectural transcription factor HMGA1, a well-established inducer of *NOS2* [213,214], subsequent studies identified *NOS2* itself as a direct target of miR-26a-5p [298,299].

Collectively, the studies by Zhu et al. [298] and Rasheed et al. [299] provide strong evidence for direct post-transcriptional regulation of *NOS2* by miR-26a-5p. In ALK (‘anaplastic lymphoma kinase’)-positive anaplastic large-cell lymphoma, Zhu et al. [298] demonstrated inverse regulation of *NOS2* by miR-26a through gain- and loss-of-function approaches, analyses of NOS2 mRNA and protein expression, luciferase reporter assays containing the *NOS2* 3′UTR, binding-site mutagenesis, and rescue experiments using *NOS2* constructs lacking the 3′UTR. These studies established sequence-specific repression of *NOS2* by miR-26a and further showed that constitutive NPM-ALK (‘nucleophosmin-anaplastic lymphoma kinase’)/STAT3 signaling promotes *NOS2* expression indirectly through repression of miR-26a [298]. Independent validation was subsequently provided by Rasheed et al. [299] in primary osteoarthritis chondrocytes, where bioinformatic prediction of conserved miR-26a-5p recognition sites, *NOS2* 3′UTR luciferase reporter assays, and miR-26a-5p mimic and inhibitor experiments demonstrated reciprocal effects on *NOS2* mRNA, iNOS protein, and NO production. Moreover, the authors showed that IL-1β-induced NF-κB activation suppresses miR-26a-5p expression, thereby relieving miR-26a-mediated repression of *NOS2* and enhancing iNOS activity [299]. Together, these studies indicate that inflammatory and oncogenic signaling pathways can converge on the miR-26a/*NOS2* axis, with STAT3 and NF-κB promoting *NOS2* expression at least in part by attenuating miR-26a-dependent post-transcriptional control.

Similarly, miR-26a-5p-mediated repression of *NOS2* has been reported in gastric cancer cells, where TET (‘ten-eleven translocation’) proteins function as competing endogenous RNAs (ceRNAs) that sequester miR-26a-5p and thereby restore *NOS2* expression [300]. TET-dependent miR-26a-5p sequestration also increases expression of the histone methyltransferase EZH2 [249,300]. Because EZH2 represses *NOS2* [249], the observed induction of *NOS2* is most likely attributable to direct relief of miR-26a-5p-mediated repression rather than to EZH2-dependent effects.

#### 5.4.2. MiR-939-5p

Strong experimental evidence supports direct post-transcriptional regulation of human *NOS2* by miR-939-5p. Guo et al. [301] identified five putative miR-939 binding sites within the human *NOS2* 3′-untranslated region (UTR) and demonstrated that two of these sites are functionally required for *NOS2* repression using site-directed mutagenesis. Direct interaction was further supported by luciferase reporter assays, Argonaute pull-down experiments, and gain- and loss-of-function studies in primary human hepatocytes. Notably, miR-939-5p reduced cytokine-induced iNOS protein expression and NO production without affecting *NOS2* mRNA abundance, indicating that translational repression is the predominant mechanism of regulation [301]. The authors further demonstrated that inflammatory cytokines induce endogenous miR-939 expression, suggesting the existence of a negative feedback loop that limits excessive *NOS2* induction during inflammatory responses [301].

Regulation of *NOS2* by miR-939-5p has subsequently been confirmed in additional cellular systems, including endothelial and cardiomyocyte models [302]. In HUVECs and H9C2 cells, miR-939-5p mimics reduced NOS2 mRNA expression, iNOS protein abundance, and NO production, whereas antagomirs exerted opposite effects. These studies also demonstrated that miR-939-5p protects endothelial and myocardial cells from cytokine-induced apoptosis and identified *TNFA* as an additional direct target of miR-939-5p, placing *NOS2* regulation within a broader anti-inflammatory network [302].

Colonic miR-939 expression is reduced in inflammatory lesions from patients with UC [303], and decreased miR-939-5p levels have also been associated with poor clinical outcomes in colorectal cancer [304]. Additional support for the biological relevance of the miR-939-5p/*NOS2* axis was provided in triple-negative breast cancer, where miR-939-5p overexpression reduced *NOS2* expression and NO production, resulting in decreased cell viability, migration, and colony-forming capacity. These effects were reversed by exogenous NO donors, supporting a functional role for the miR-939-5p/NOS2/NO pathway in tumor progression [305].

Several competing endogenous RNA (ceRNA) mechanisms have been reported to antagonize miR-939-5p activity. LncRNA HEIH functions as a miR-939-5p sponge, thereby relieving repression of *NOS2* and promoting NO-dependent tumor growth and migration in triple-negative breast cancer cells [305]. Consistent with this mechanism, HEIH is expressed in both normal and malignant colonic epithelial cells and is overexpressed in colorectal cancer, where its elevated expression is associated with poor prognosis [306]. HEIH has also been implicated in cholangiocarcinoma [307], a malignancy of particular relevance in IBD-associated hepatobiliary disease.

An additional layer of regulation is provided by the *NOS2* pseudogene transcript, ‘Nitric Oxide Synthase 2 Pseudogene 3’ (NOS2P3), which acts as a miR-939-5p sponge [302]. In endothelial cells, NOS2P3 increases expression of both *NOS2* and *TNFA* in a dose-dependent manner by sequestering miR-939-5p and limiting its inhibitory activity. Accordingly, manipulation of NOS2P3 alters cytokine-induced apoptosis through the miR-939-5p/NOS2/TNFα axis, further illustrating the complexity of post-transcriptional regulation governing *NOS2* expression [302].

#### 5.4.3. MiR-146a and lncRNA HCG18 and CHR

Direct inhibition of *NOS2* by miR-146a has been demonstrated in mice [308]. MiR-146a is expressed predominantly in intestinal leukocytes, and miR-146a-deficient animals exhibit more than threefold higher *NOS2* transcript levels than wild-type mice. In this setting, enhanced *NOS2* expression reduces susceptibility to dextran sulfate sodium (DSS)-induced colitis, suggesting a negative regulatory role of miR-146a in intestinal homeostasis [309]. Additional evidence for direct *NOS2* regulation was provided by Simanovich et al. [310], who showed that miR-146a-5p suppresses *NOS2* translation in lipopolysaccharide- and IFNγ-stimulated mouse colon cancer cells (CT26). Antagonism of miR-146a-5p restored NOS2 protein expression and enhanced macrophage-mediated NO-dependent tumor cell killing, indicating that miR-146a-mediated *NOS2* repression may promote tumor survival [310].

Dysregulation of miR-146a and the closely related miR-146b has been reported in human IBD and linked to impaired immune responses and mucosal barrier integrity [292]. Beyond its intracellular functions, miR-146a can also be transferred between immune cells via exosomes. Alexander et al. [311] demonstrated that dendritic cells release exosomes containing miR-146a, which are taken up by recipient dendritic cells and suppress inflammatory responses to endotoxin stimulation. In vivo, exosome-mediated transfer of miR-146a attenuated inflammation, supporting a role for miR-146a as an intercellular anti-inflammatory signal that contributes to the maintenance of immune homeostasis [311]. MiR-146a is elevated in colonic tissue from UC patients and in inflamed mucosa of pediatric IBD patients [309,312], while Zhu et al. [313] reported increased expression of miR-146a-3p in both active CD and inactive CD/UC. Interestingly, miR-146a haplodeficiency aggravated DSS-induced colitis in their model, contrasting earlier observations in miR-146a knockout mice [309,313]. These discrepancies may, at least in part, reflect differences in mouse strains and experimental settings [313]. Notably, efforts to identify direct interaction between miR-146a and the human *NOS2* transcript have been less successful than for miR-26a. Zhu et al. [298] were unable to identify putative miR-146a-binding sites within the human *NOS2* 3′UTR, whereas miR-26a was predicted and experimentally confirmed to directly repress *NOS2* expression.

Although human *NOS2* is therefore not considered a major direct target of miR-146a, the microRNA can indirectly suppress *NOS2* expression through several pathways. Induced by NF-κB, miR-146a/b establishes a negative-feedback loop by repressing kinase *IRAK1* and adaptor protein *TRAF6*, key mediators of IL-1R- and TLR-dependent activation of NF-κB [314]. In addition, miR-146a directly inhibits *STAT1* translation, thereby attenuating IFNγ/JAK/STAT signaling, the second principal pathway driving *NOS2* expression.

Consistent with these actions, miR-146a suppresses M1 macrophage polarization and promotes M2 differentiation. These effects can be reversed by the lncRNAs HCG18 and CHRF, which act as miR-146a sponges. Beyond macrophages, miR-146a supports Treg function and dampens pro-inflammatory and cytotoxic activities of dendritic and natural killer (NK) cells, respectively (reviewed in [315]). Despite its predominantly anti-inflammatory properties, miR-146a may also protect against colitis-associated carcinogenesis. In an azoxymethane-induced mouse model, miR-146a limited tumor development by suppressing IL-17/IL-17R signaling through repression of kinase *RIPK2* in myeloid cells and *TRAF6* in intestinal epithelial cells [316].

#### 5.4.4. MiR-155

MiR-155 is an NF-κB-inducible miRNA that amplifies inflammatory responses. It is overexpressed in inflammatory lesions from adult [292,317,318,319,320] and pediatric [321] patients with IBD, as well as in colorectal cancer [322], although associations with disease phenotype and activity remain inconsistent. Inflammatory stimuli induce miR-155 expression in both immune cells and human intestinal epithelial cells (HT-29) [320,321].

MiR-155 regulates multiple immune-cell populations involved in intestinal inflammation. It influences Treg and Th17 differentiation and promotes Th1-cell responses by targeting *SOCS1* (‘suppressor of cytokine signaling 1’) [323]. It also serves as a key regulator of the M1 macrophage transcriptional program [324,325], of which NOS2 expression is a hallmark. Accordingly, miR-155 overexpression drives repolarization of M2 and tumor-associated macrophages toward an M1 phenotype, accompanied by increased NOS2 expression, whereas its depletion promotes M2 polarization and reduces NOS2 levels [326]. Consistent with its pro-inflammatory functions, miR-155 is among the most highly expressed UC-associated miRNAs in blood [327], and its deficiency attenuates intestinal inflammation in experimental colitis [328].

Although miRTarBase lists *NOS2* as a validated target of miR-155 [295], experimental evidence supporting direct regulation is limited. Instead, most studies indicate indirect effects on *NOS2* expression. Although miR-155 can suppress selected inflammatory pathways by targeting mRNAs of C/EBPβ [329], MyD88 [330], TAB2 [331], and GSK-3β [332,333], its net effect in the intestine is generally pro-inflammatory and associated with enhanced *NOS2* expression. This reflects the predominant repression of anti-inflammatory regulators, including *SMAD2* [334], *BCL6* [335], *SHIP1* (phosphoinositide phosphatase), and *SOCS1* [323,336], which collectively augment TGFβ-, NF-κB-, PI3K/Akt-, and JAK/STAT-dependent signaling pathways and favor M1 macrophage polarization.

In UC mucosa, increased miR-155 expression is accompanied by reduced levels of the transcription factor FOXO3a. In HT-29 cells, TNFα-induced miR-155 directly suppresses *FOXO3A*, resulting in reduced IκBα expression and enhanced NF-κB activation [320]. Likewise, miR-155 promotes NF-κB signaling and *NOS2* expression in injured arteries and vascular smooth muscle cells. It represses *MST2,* encoding ‘mammalian sterile 20-like kinase 2’—a component of the Hippo pathway regulating Raf-1/ERK activity [337].

MiR-155 may also mediate the effects of other miRNAs on *NOS2* expression. For example, miR-342-5p indirectly stimulates *NOS2* expression by targeting *AKT1*, a negative regulator of miR-155. Consequently, *AKT1* suppression leads to increased miR-155 expression and enhanced *NOS2* induction in activated macrophages [338]. Notably, the functional consequences of miR-155 may depend on both timing and strand selection. As reviewed by Dawson et al. [339], miR-155-3p promotes early inflammatory responses in dendritic cells through repression of inhibitory kinase IRAK-M, whereas miR-155-5p dampens them during later phases by targeting adaptor protein TAB2, transducing cytokine and microbial signals.

#### 5.4.5. MiR-29

MiR-29a [41,292,320] and miR-29b [292,320] are overexpressed in both inflamed and non-inflamed mucosa from patients with UC, with miR-29a levels being significantly higher in inflamed than in non-inflamed tissue [41]. Similarly, patients with CD exhibit increased miR-29b expression in both inflamed and non-inflamed mucosa [292] and elevated circulating levels of miR-29a [327].

Although miR-29 does not directly target *NOS2*, it may potentially modulate its expression through the regulation of several upstream signaling pathways and transcriptional regulators. Notably, miR-29 represses IL-12p40, a subunit shared by IL-12 and IL-23, as well as activating transcription factor 2 (ATF2), which regulates expression of the IL-23-specific subunit IL-23p19 [340]. Since IL-23 signaling promotes *NOS2* expression [341] and plays a central role in IBD pathogenesis, with IL-23-deficient animals exhibiting resistance to experimental colitis (reviewed in [342]), miR-29 may indirectly limit *NOS2* induction through suppression of the IL-23 pathway.

In addition, ATF2 has been shown to positively regulate *NOS2* expression in microglia via activation of the TRAF6-p38/JNK-ATF2 signaling pathway following stimulation with lipopolysaccharide [343]. Finally, miR-29b-3p suppresses *HMGB1*, a positive regulator of *NOS2* transcription [209], in both lipopolysaccharide-stimulated Caco-2 cells and a rat colitis model [344]. Together, these findings suggest that miR-29 acts predominantly as an indirect negative regulator of *NOS2* expression by targeting multiple components of pro-inflammatory signaling pathways.

#### 5.4.6. MiR-21

MiR-21 is widely expressed in both immune and non-immune cells and plays an important role in the regulation and resolution of inflammatory responses [345]. Consistent with this function, miR-21 is overexpressed in the intestinal mucosa of patients with IBD, although reported associations with disease phenotype and activity remain inconsistent [41,292,317,318,319,320,346]. Its expression is observed predominantly in lamina propria mononuclear cells and, to a lesser extent, in epithelial cells [346]. MiR-21 is also upregulated in both sporadic colorectal cancer and colitis-associated cancer, with the highest expression levels detected in the latter [319]. In pediatric IBD, miR-21 is similarly elevated in both UC and CD, and miR-21-5p forms part of a seven-miRNA panel capable of predicting residual disease activity in CD after three months of treatment [347]. Reports regarding circulating miR-21 levels are less consistent, with studies describing increased [327], decreased [317], or unchanged concentrations relative to healthy controls [348].

Both miR-21 [349] and *NOS2* are induced downstream of NF-κB activation in immune cells. Reflecting its predominantly anti-inflammatory role, miR-21-5p has been reported to directly target *TLR4* in macrophages and human monocytic cells (THP-1), thereby dampening inflammatory responses during *Mycobacterium tuberculosis* infection [350]. Consistently, miR-21-deficient macrophages infected with *M. tuberculosis* display increased *NOS2* expression [351]. Likewise, genetic deletion of miR-21 leads to upregulation of IRAK2 and IRAK4 [352], key kinases mediating TLR- and IL-1R-dependent inflammatory signaling [353]. Notably, IRAK2 has also been implicated in the nuclear export of inflammatory mRNAs [354].

Additional indirect effects of miR-21 on *NOS2* may involve regulation of cellular metabolism. MiR-21 suppresses methylthioadenosine phosphorylase (MTAP) [355], a key enzyme of methionine and adenine salvage pathways, resulting in accumulation of 5′-deoxy-5′-methylthioadenosine (MTA). MTA inhibits polyamine synthesis via the competitive arginase-1 pathway while promoting *NOS2* expression and M1 macrophage polarization [356]. Conversely, miR-21 may restrain *NOS2*-inducing inflammatory pathways by targeting *PDCD4* (‘programmed cell death protein 4’), a mediator of lipopolysaccharide-induced NF-κB activation and IL-10 suppression [348]. In addition, miR-21 targets tumor suppressor *PTEN* (‘phosphatase and tensin homolog deleted on chromosome 10’), which has recently been implicated in promoting a pro-inflammatory phenotype in tumor-associated macrophages [357].

Collectively, these findings suggest that miR-21 exerts complex and context-dependent effects on *NOS2* expression, integrating inflammatory, metabolic, and immune-regulatory signals.

#### 5.4.7. MiR-126

MiR-126 is upregulated in the colonic mucosa of patients with UC [292,320,358] and CD [292], as well as in the blood of patients with CD [327]. Increased expression has also been reported in inflamed lesions from pediatric IBD patients [312,347]. Both the 3p and 5p strands of miR-126 are highly expressed in endothelial cells and play important roles in vasculogenesis, angiogenesis, lymphangiogenesis, and endothelial repair following injury [359]. Consistent with these functions, elevated miR-126 expression in UC has been localized predominantly to the intestinal endothelium [346].

MiR-126 may influence *NOS2* expression indirectly through modulation of NF-κB signaling. Specifically, miR-126 represses *IKBA*, encoding a key inhibitor of NF-κB and, consequently, of NF-κB-dependent *NOS2* transcription. IκBα expression is reduced in active UC lesions, but not in inactive UC, irritable bowel syndrome, or normal mucosa, and inversely correlates with miR-126 levels [358]. Furthermore, experimental stabilization of IκBα protects against colitis, supporting the functional importance of this pathway [125].

In contrast, miR-126 may also limit *NOS2* expression by targeting *HMGB1* in endothelial cells [360]. Because HMGB1 facilitates *NOS2* transcription [209], suppression of *HMGB1* represents an additional mechanism through which miR-126 could modulate inflammatory NO production. Collectively, these findings suggest that miR-126 exerts context-dependent effects on *NOS2* regulation through its influence on both NF-κB and HMGB1 signaling pathways.

#### 5.4.8. MiR-98-5p and lncRNA MEG3

MiR-98-5p, a microRNA implicated in the regulation of inflammatory responses, is upregulated in the intestinal mucosa of patients with UC, although studies have yielded conflicting results regarding whether its expression is higher in active or inactive disease [361]. Using a murine model of IBD, Peng et al. [362] demonstrated that both miR-98-5p and NOS2 were significantly upregulated in inflamed colonic tissue compared with healthy controls.

Mechanistic studies revealed that, in peritoneal macrophages stimulated with lipopolysaccharide, miR-98-5p promotes M1 polarization and inflammatory responses by suppressing *TRIB1* (‘tribbles homolog 1’), encoding a positive regulator of the M2 phenotype. Inhibition of miR-98-5p reduced gene expression of proinflammatory NOS2, TNFα, and monocyte chemoattractant protein-1 (CCL2), while increasing expression of M2 markers, including arginase-1. Conversely, TRIB1 deficiency enhanced NOS2 expression and suppressed arginase-1 [362]. In vivo, antagonism of miR-98-5p attenuated intestinal inflammation and shifted macrophage polarization toward an M2 phenotype, as evidenced by reduced NOS2 and increased arginase-1 expression [362].

MiR-98-5p is negatively regulated by the lncRNA MEG3, which acts as a molecular sponge. Consistent with the pro-inflammatory role of miR-98-5p, lncMEG3 protects against excessive inflammation, oxidative stress, and apoptosis in murine models of colitis [363]. These findings suggest that the MEG3/miR-98-5p/TRIB1 axis contributes to the regulation of macrophage polarization and may indirectly influence *NOS2* expression during intestinal inflammation.

#### 5.4.9. MiR-9

Fasseu et al. [292] identified miR-9 as being overexpressed in inflamed mucosa from CD patients. Functional studies have shown that miR-9 directly represses *RUNX1* (‘runt-related transcription factor 1’) in myeloid-derived suppressor cells, leading to restoration of *NOS2* expression and NO production. These findings suggest opposite roles for the two molecules, with RUNX1 acting as a negative and miR-9 as a positive regulator of *NOS2* expression [364].

*RUNX1* is among the candidate genes associated with IBD susceptibility [215] and progression to colorectal cancer [365]. Beyond its role in *NOS2* regulation, RUNX1 participates in multiple aspects of mucosal immunity, regulating the development and function of T cells and innate lymphoid cells (reviewed in [366]). Evidence from mouse models indicates that RUNX1 contributes to immune homeostasis by supporting regulatory T-cell function and limiting aberrant T-cell activation. Disruption of the RUNX1-CBFβ complex in regulatory T cells impairs suppressive activity and promotes immune dysregulation [367], whereas RUNX1 deficiency in CD4^+^ T cells results in spontaneous activation and severe autoimmune inflammation [368].

RUNX1 has also been implicated in the conversion of Th1 to Th17 cells within the inflamed intestine following induction by TGFβ. Consistent with this mechanism, TGFβ, a known suppressor of *NOS2* expression [75], induces RUNX1 expression and enhances its transcriptional activity by increasing accessibility of RUNX1 target-gene promoters [369]. Moreover, expression of RUNX1 and NOS2 is inversely correlated in M1-polarized macrophages, in line with the association of RUNX1 with the anti-inflammatory M2 phenotype [370]. Collectively, these observations suggest that miR-9 may indirectly promote *NOS2* expression through repression of *RUNX1*, thereby influencing macrophage polarization and inflammatory responses in the intestinal mucosa.

#### 5.4.10. MiR-369-3p

MiR-369-3p has been implicated as a negative regulator of intestinal inflammation and is downregulated in inflamed mucosa from patients with IBD [371], as well as in the colons of dextran sulfate sodium (DSS)-treated mice, particularly in colonic and lipopolysaccharide-stimulated bone marrow-derived dendritic cells [371,372]. Mechanistically, miR-369-3p targets both TNFα and C/EBPβ [372], potentially attenuating *NOS2* induction driven by pro-inflammatory cytokines. More recently, a binding site for miR-369-3p was identified in silico within the *NOS2* coding sequence, and the miRNA has been shown to directly suppress NOS2 expression in mouse dendritic cells at both the mRNA and protein levels [371].

In addition to inhibiting NOS2, miR-369-3p reduces the production of several pro-inflammatory mediators, including IL-1α, IL-1β, IL-6, and IL-12, while promoting secretion of the anti-inflammatory cytokine IL-10 and the IL-1 decoy receptor (IL1RA) by dendritic cells [371]. Furthermore, miR-369-3p inhibits p65 phosphorylation and prevents NF-κB nuclear translocation, thereby suppressing transcription of multiple NF-κB-dependent inflammatory genes, including *NOS2* [371].

Collectively, these findings identify miR-369-3p as a possible direct and indirect negative regulator of *NOS2* expression that limits inflammatory signaling in the intestinal mucosa.

#### 5.4.11. MiR-200

The miR-200 family is principally involved in maintaining epithelial identity and is therefore extensively studied in the context of cancer and epithelial–mesenchymal transition. Its best-characterized member, miR-200c, is upregulated in the blood of patients with CD [327] but downregulated, together with miR-200a, in active UC lesions compared with normal colonic mucosa from healthy individuals [320]. Similarly, miR-200b expression is reduced in active UC relative to inactive UC [41], while another family member, miR-141, is downregulated in inflamed lesions from pediatric patients with CD and UC [312].

MiR-200 family members regulate multiple targets implicated in inflammation and carcinogenesis. Depending on the cellular context, miR-200c may exert oncogenic effects through repression of *PTEN* or *KLF6*, or tumor-suppressive effects through inhibition of *HMGB1*, *HIF1A*, and other targets (reviewed in [373]). Consequently, downregulation of KLF6, HMGB1, and HIF1α may indirectly reduce *NOS2* expression. In addition, kinase ROCK2 has been identified as a direct miR-200 target [374]. Since ROCK1/2 signaling promotes *NOS2* expression in myeloid cells, including dendritic cells and macrophages [375], and contributes to NF-κB-dependent inflammatory responses in neutrophils [376] and microglia [377], inhibition of the ROCK pathway represents another mechanism through which miR-200 family members may negatively regulate NOS2 expression. Collectively, these findings suggest that miR-200 family members influence NOS2 largely through indirect mechanisms involving KLF6, HMGB1, HIF1α, and ROCK-dependent inflammatory signaling pathways.

#### 5.4.12. MiR-31

Elevated expression of miR-31 has been reported in inflamed mucosa from adult patients with CD and UC, as well as in non-inflamed CD tissue [292,317]. Similar findings have been observed in pediatric IBD, where increased miR-31 expression discriminated UC from CD patients [312]. Fang et al. reported significant upregulation of miR-31-3p specifically in UC biopsies from adult patients [378], whereas Schaefer et al. detected increased miR-31 levels in saliva from patients with UC but reduced circulating levels in both CD and UC [317].

Functionally, miR-31 attenuates experimental colitis and suppresses inflammatory responses in colonocytes through repression of RhoA (‘Ras homolog family member A’) GTPase [378]. Although its direct effect on *NOS2* expression has not been examined, RhoA is known to promote *NOS2* induction in several inflammatory settings via MEK1/ERK1/2 [379,380], NF-κB (IKKα)-CBP/p300 [381], and RhoA/ROCK1 signaling pathways [377]. Accordingly, miR-31 may indirectly limit *NOS2* expression by dampening RhoA-dependent pro-inflammatory signaling.

Collectively, these findings suggest that miR-31 exerts protective effects in intestinal inflammation and may contribute to suppression of NOS2-inducing pathways through inhibition of RhoA signaling.

#### 5.4.13. MiR-16

MiR-16 is upregulated in the blood of patients with both CD and UC [327,382], with expression levels correlating with the extent and severity of CD [382]. Increased miR-16 expression has also been reported in UC mucosa, where levels are highest in inflamed tissue but remain elevated in non-inflamed areas as well [41].

MiR-16 may indirectly promote *NOS2* expression through repression of adenosine receptor A2A (A2AR) signaling (reviewed in [383]). Activation of the adenosine/A2AR pathway negatively regulates *NOS2* expression in immune cells [384] and plays an important role in limiting inflammatory responses mediated by macrophages, neutrophils, and dendritic cells [383]. Consistent with its anti-inflammatory function, A2AR signaling is dysregulated in IBD, and enhancement of this pathway reduces intestinal inflammation and the accumulation of pro-inflammatory immune cells in the gut mucosa [385].

In intestinal epithelial cells, miR-16 has been shown to restore NF-κB activity by suppressing adenosine/A2AR signaling [386]. Thus, although no direct interaction between miR-16 and NOS2 has been reported, miR-16 may indirectly facilitate *NOS2* induction by relieving the inhibitory effects of adenosine/A2AR signaling on NF-κB-dependent inflammatory pathways.

#### 5.4.14. MiR-124

MiR-124 expression is markedly reduced in inflamed colonic biopsies from pediatric, but not adult, patients with UC, suggesting a particular role for this miRNA in pediatric disease [387]. Its downregulation is accompanied by increased levels of phosphorylated STAT3, a direct target of miR-124, in both patient samples and experimental colitis models [387].

This finding is of potential relevance to NOS2 regulation, as the IL-6R/STAT3 pathway contributes to *NOS2* induction, and pharmacological inhibition of this pathway with fenofibrate reduces NO production in patients with IBD [388]. In addition, miR-124 is induced in monocytes exposed to IL-4 and IL-13, where it promotes M2 macrophage polarization. Conversely, miR-124 silencing results in increased *NOS2* expression, further supporting a role for this miRNA as a negative regulator of pro-inflammatory macrophage activation (reviewed in [389]). Collectively, these observations suggest that miR-124 may indirectly suppress *NOS2* expression through inhibition of STAT3 signaling and promotion of anti-inflammatory M2 macrophage polarization.

#### 5.4.15. MiR-4262

MiR-4262 is overexpressed in colonic mucosal samples from children with IBD compared with normal mucosa from non-IBD controls. Similarly, its expression is increased in Caco-2 cells treated with dextran sulfate sodium (DSS) [390]. Functional studies have shown that miR-4262 directly targets and represses the deacylase sirtuin-1 [390], thereby indirectly influencing *NOS2* expression.

Sirtuin-1 exerts anti-inflammatory and antioxidant effects through deacetylation of key components of the NF-κB and AP-1 pathways, including p65 and c-Jun. In addition, sirtuin-1 stabilizes IκBα and thereby limits NF-κB activation in macrophages stimulated with lipopolysaccharide (reviewed in [391]). Consistent with these functions, sirtuin-1 activation, or treatment with sirtuin-1 analogs, suppresses *NOS2* expression in diverse biological contexts, including chondrocytes, bone marrow-derived macrophages treated with lipopolysaccharide, microglia, intestinal ischemia–reperfusion injury, and cardiovascular disease-associated monocytes (reviewed in [391,392]).

Interestingly, sirtuin-1 and NOS2 participate in a reciprocal regulatory loop. While sirtuin-1 suppresses NF-κB- and AP-1-dependent *NOS2* expression, NOS2-derived NO can, in turn, inhibit sirtuin-1 activity through S-nitrosylation of the protein, thereby relieving repression of these pro-inflammatory pathways [391]. Consequently, upregulation of miR-4262 may promote intestinal inflammation by suppressing sirtuin-1 and enhancing conditions that favor *NOS2* expression.

#### 5.4.16. Lnc–CHOP and Lnc–C/EBPβ

NOS2 expression is a characteristic feature of the immunosuppressive phenotype of myeloid-derived suppressor cells (MDSCs), where it contributes, among other functions, to T-cell apoptosis [393]. Recent studies have shown that *NOS2* expression is reciprocally regulated by the long non-coding RNAs lnc-CHOP and lnc-C/EBPβ through interactions with CHOP (‘C/EBP homologous protein’; GADD153) and the LIP isoform of C/EBPβ, respectively.

Binding of lnc-C/EBPβ to LIP stabilizes the transcriptionally inactive LIP-LAP complex, thereby suppressing expression of MDSC effector genes, including *NOS2* [394]. In contrast, lnc-CHOP interacts with CHOP and promotes its association with LIP, facilitating release of the transcriptionally active LAP isoform. This process enhances C/EBPβ-dependent transcription and induces *NOS2* expression [395]. In addition, lnc-CHOP promotes activating H3K4 methylation at target loci, further supporting *NOS2* transcription [395].

Together, these findings identify lnc-CHOP and lnc-C/EBPβ as opposing regulators of *NOS2* expression in MDSCs, acting through coordinated control of C/EBPβ activity and chromatin accessibility.

#### 5.4.17. MiR-185 and lncRNA RNCR3

CHOP, a positive regulator of *NOS2* expression in MDSCs [394,395], is negatively regulated by miR-185-5p. This inhibition can be relieved by the lncRNA RNCR3 (‘retinal non-coding RNA 3’), which acts as a molecular sponge for miR-185-5p, thereby restoring CHOP expression [396].

MiR-185-5p contributes to the maintenance of intestinal barrier integrity and exhibits tumor-suppressive properties. Consistent with these functions, its expression is reduced in both inflamed colonic mucosa from patients with IBD [397] and colorectal cancer tissue [398]. Through modulation of the CHOP/C/EBPβ pathway, the RNCR3/miR-185-5p axis may indirectly influence *NOS2* expression and function of MDSCs during intestinal inflammation and tumorigenesis.

#### 5.4.18. MiR-214-3p and lncRNA Pseudogene Olfr29-ps1

The pseudogene Olfr29-ps1, acting as a lncRNA, promotes *NOS2* expression and NO production in monocytic MDSCs [399]. Mechanistically, Olfr29-ps1 functions as a sponge for miR-214-3p, which negatively regulates MyD88 [399], a key adaptor of TLR- and IL-1R-dependent NF-κB signaling involved in host–microbiota interactions and intestinal immune homeostasis [400]. Consistent with this role, MyD88-deficient mice exhibit increased susceptibility to infection accompanied by reduced *NOS2* expression [401].

MiR-214-3p also influences macrophage polarization. Its suppression in M0 macrophages promotes M1 polarization and increases *NOS2* expression, whereas its overexpression inhibits M1 differentiation. The anti-inflammatory effects associated with its overexpression have been linked to repression of transferrin receptor protein 1 and reduced ferroptosis [402]. Notably, Olfr29-ps1 expression is induced by IL-6, which promotes the N6-methyladenosine (m6A) modification required for interaction between Olfr29-ps1 and miR-214-3p [399].

Additional evidence suggests that miR-214-3p regulates the STAT6 pathway. In UC biopsies, miR-214-3p expression is decreased, whereas STAT6 expression is increased relative to controls [403]. In macrophages, STAT6 mediates the inhibitory effects of IL-4 and IL-13 on the NOS2/NO pathway, primarily through induction of arginase-1 and subsequent L-arginine depletion rather than direct suppression of *NOS2* expression [404]. In contrast, STAT6 exerts protective effects in intestinal epithelial cells, where its deficiency enhances apoptosis induced by dextran sulfate sodium (DSS) and exacerbates intestinal inflammation [405].

Beyond its role in inflammation, miR-214-3p also functions as a tumor suppressor. Its expression is reduced in colorectal cancer, where it directly targets ARL2 (‘ADP-ribosylation factor-like protein 2’) and inhibits tumor progression [406]. Collectively, these findings suggest that the Olfr29-ps1/miR-214-3p axis modulates *NOS2* expression indirectly through regulation of MyD88-dependent inflammatory signaling and macrophage polarization.

### 5.5. Reading Frame Variation and Translational Control

In addition to classical exon skipping, more recent studies have revealed further complexity in *NOS2* transcript architecture. Multiple *NOS2* mRNA variants differing in 5′-UTR structure and first-exon usage have been identified. Notably, transcripts lacking exon 1 are more abundant in both unstimulated and stimulated intestinal epithelial cells (DLD-1 and T84) [259]. Such variation can influence translation efficiency and protein production independently of alterations in the coding sequence.

Chu et al. [259] reported that the human *NOS2* 5′-UTR contains eight partially overlapping upstream open reading frames (uORFs) located before the main ATG start codon, suggesting an additional layer of translational regulation. Transcripts containing uORFs are common targets of nonsense-mediated mRNA decay (NMD). Although NMD primarily functions as an mRNA surveillance pathway that prevents translation of aberrant transcripts encoding potentially toxic truncated proteins, it also contributes to fine-tuning the expression of genes involved in stress responses and immunity [407].

Based on studies in DLD-1 cells, Gather et al. [408] proposed that *NOS2* translation involves a leaky scanning mechanism, as translation of the upstream ORF did not impair translation of the main coding sequence. Interestingly, the upstream ORF contains a stop codon located 39 bp upstream of intron 1, a structural feature commonly associated with NMD-sensitive transcripts. Consistent with this hypothesis, the silencing of UPF1, an RNA helicase essential for NMD activation, enhanced cytokine-induced *NOS2* expression in DLD-1 cells [408]. Since UPF1 promotes degradation of NMD-targeted transcripts by removing associated proteins and facilitating nuclease access [407], these findings suggest that NMD contributes to post-transcriptional regulation of *NOS2* expression.

Together, these observations indicate that variation in 5′-UTR structure, uORF utilization, and NMD-dependent transcript turnover adds another regulatory layer controlling *NOS2* expression and may influence cellular responsiveness to inflammatory stimuli.

### 5.6. Implications for IBD and Intestinal Inflammation

Contrary to earlier assumptions, *NOS2* expression is regulated not only at the transcriptional level but also through multiple post-transcriptional mechanisms, including alternative splicing, control of mRNA stability and nuclear export, translational regulation, and modulation by non-coding RNAs. These processes add several context-dependent layers of regulation that enable fine-tuning of NO production in response to inflammatory and environmental cues. Many post-transcriptional regulators of *NOS2*, particularly microRNAs and long non-coding RNAs, are dysregulated in the intestinal mucosa and circulation of patients with CD and UC. Several remain altered even in non-inflamed tissue, suggesting a contribution to disease susceptibility and persistence rather than merely reflecting ongoing inflammation. By modulating key pathways involved in *NOS2* induction, including NF-κB, IFNγ/STAT1, TGFβ, and macrophage polarization, these regulators may influence both the magnitude and duration of intestinal inflammatory responses.

Beyond inflammation, post-transcriptional mechanisms may contribute to colitis-associated carcinogenesis by modifying cellular responses to nitrosative stress, proliferation, and survival. Collectively, these observations highlight post-transcriptional regulation as an important determinant of NOS2 activity in IBD and a potential source of biomarkers and therapeutic targets.

## 6. Regulation of NOS2 Protein and Its Catalytic Activity

Compared with other NOS isoenzymes, regulation of NOS2 at the protein level remains relatively understudied. Nevertheless, accumulating evidence indicates that NOS2 activity is controlled through regulation of dimer assembly, subcellular localization, catalytic activity, and protein turnover. These processes are frequently modulated by protein–protein interactions and post-translational modifications, including tyrosine nitration, S-nitrosylation, phosphorylation, ubiquitination, and acylation. An overview of these mechanisms is presented in Figure 4.

### 6.1. NOS2 Assembly

All NOS isoenzymes are synthesized as monomers, but their catalytically active form is a homodimer. Dimerization requires heme, L-arginine, and tetrahydrobiopterin, with tetrahydrobiopterin exerting a stronger stabilizing effect than L-arginine [409,410]. Heme incorporation is ATP-dependent and facilitated by HSP90A; its inhibition (e.g., by radicicol) nearly abolishes NOS2 assembly [411].

Among NOS isoenzymes, NOS2 forms the least stable dimers and is therefore most prone to disassembly [409,410]. NO and its derivatives are key regulators of this process. Excessive NO production, such as during macrophage activation, can inhibit NOS2 dimerization by limiting heme availability [412]. Additionally, NO-derived species can interfere with dimer formation through coordination with heme Fe^3+^ after its incorporation [413].

Post-translational modifications further destabilize NOS2. Tyrosine nitration by reactive oxygen and nitrogen species (e.g., peroxynitrite or NO_2_•) promotes monomerization and loss of activity, with Tyr^299^, Tyr^336^, Tyr^446^, and Tyr^698^ identified as targets [414]. Similarly, S-nitrosation of cysteine residues within the tetrathiolate zinc-binding motif leads to Zn loss and dimer disruption [415].

Small molecules can modulate NOS2 dimerization. Imidazole promotes dimer formation, whereas bulkier derivatives such as clotrimazole inhibit it [416]. Pyrimidine imidazole derivatives (PIDs) bind NOS2 heme, initially thought to trap the enzyme in an inactive monomeric state. However, they also bind dimers, inducing their dissociation and preventing reassembly, with catalytic inhibition preceding physical separation [417]. The inhibitory effects of PIDs may be clinically relevant because, in addition to its antifungal activity, clotrimazole has been shown to reduce mucosal inflammation by limiting inflammatory and angiogenic responses [418]. Given the involvement of NOS2-derived NO in both processes, this mechanism may contribute to the anti-inflammatory activity of clotrimazole alongside suppression of the NF-κB/IL-8 pathway [418].

Cells also employ regulatory proteins to limit NOS2 activity. In murine macrophages, NAP110 (‘NOS-associated protein 110 kDa’) binds the N-terminus of NOS2 and prevents dimerization; its expression is induced by inflammatory stimuli [419]. NAP110, also referred to as ADRM1 or Rpn13, has subsequently been implicated in proteasomal degradation of NOS2 [420]. The N-terminus also interacts with kalirin, a Rho guanine exchange factor, which similarly inhibits dimerization [421].

NOS2 assembly is further supported by phosphatidylinositol 3-kinase (PI3K). In macrophages, deficiency of its p85α regulatory subunit markedly reduces NO production, likely due to impaired tetrahydrobiopterin synthesis via reduced induction of GTP cyclohydrolase 1, a regulatory enzyme of the pathway [422].

### 6.2. Substrate Availability

L-arginine availability is a key determinant of NOS2 activity. In intestinal epithelial cells, it derives from dietary or endogenous protein degradation and de novo synthesis. Because demand may exceed supply under certain conditions, L-arginine is considered semi-essential.

**Figure 5:**
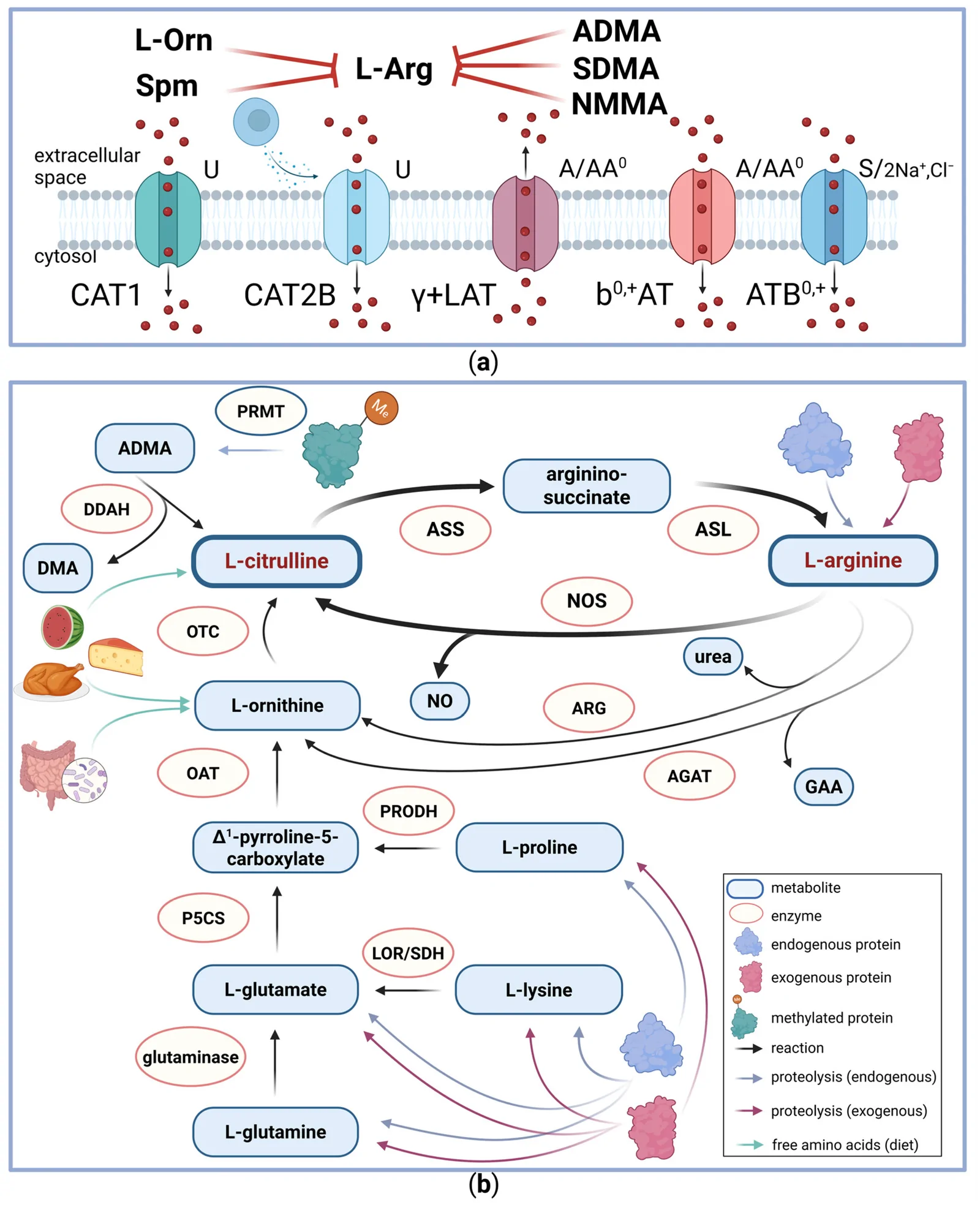
Availability of L-arginine: (**a**) transporters for L-arginine and its competitors; (**b**) intracellular sources of L-arginine, including the L-citrulline–NO cycle. Krzystek-Korpacka, M. (2026) https://core.local.biorender.dev/api/shortlink/evu9u57. ADMA, asymmetric dimethylarginine; AGAT, arginine:glycine amidinotransferase; ARG, arginase; ASL, argininosuccinate lyase; ASS, argininosuccinate synthetase; DDAH, dimethylarginine dimethylaminohydrolase; DMA; dimethylamine; GAA, guanidinoacetate; L-Arg, L-arginine; LOR, lysine-oxoglutarate reductase; L-Orn, L-ornithine; NMMA, N^G^-monomethyl-L-arginine; OAT, ornithine aminotransferase; OTC, ornithine transcarbamylase; P5CS, delta-pyrroline-5-carboxylate synthase; PRMT, protein arginine methyltransferase; PRODH, proline dehydrogenase; SDH, saccharopine dehydrogenase; SDMA, symmetric dimethylarginine; Spm, spermine.

Transport across membranes of intestinal epithelial cells is mediated primarily by cationic amino acid transporters CAT1 (constitutive) and CAT2B (inducible by lipopolysaccharide, IFNγ, TNFα, and IL-1β), as well as antiporters γ^+^LAT1 and b^0,+^AT (Figure 5a). CAT1, CAT2B, and b^0,+^AT mediate L-arginine uptake, whereas γ^+^LAT1 primarily supports export. CAT1 and γ^+^LAT1 localize mainly to the basolateral membrane, while b^0,+^AT is apical [423,424,425].

Although CAT2B is expressed in epithelial cells [426,427], it is most prominent in immune cells, particularly macrophages [426,428]. In colonic epithelium, injury-induced CAT2B upregulation supports epithelial restitution and barrier repair, with transported L-arginine preferentially metabolized by arginase to proline [426]. In vivo, increased CAT2B expression attenuates colitis severity and limits the shift from IFNγ- to Th17-mediated responses [428], although it may also facilitate bacterial adhesion and niche establishment [429].

Transporter expression varies along the intestine: b^0,+^AT peaks in the ileum, γ^+^LAT1 in the jejunum, and ATB^0,+^—a Na^+^/Cl^−^-dependent symporter—dominates in the colon [425]. ATB^0,+^ reduces luminal amino acid availability to microbiota and is upregulated in colorectal cancer, CD, and UC [430,431,432]. L-arginine uptake is further modulated by competing amino acids (L-lysine and L-ornithine) and methylated arginine derivatives such as N^G^-monomethyl-L-arginine (NMMA) and asymmetric (ADMA) and symmetric (SDMA) dimethylarginines. Polyamines also compete for the same transporter, in addition to regulating CAT2B expression and translation [433,434].

Intracellularly, NOS2 competes for L-arginine with arginases, arginine:glycine amidinotransferase, and arginine decarboxylase [4]. Several products of the arginase pathway, including L-ornithine, putrescine, and spermine (but not spermidine), suppress *NOS2* expression and/or translation, illustrating the tight metabolic coupling between competing arginine-utilizing pathways [256,435] (Figure 3).

L-arginine can also be regenerated from L-citrulline via the L-citrulline–NO cycle, involving argininosuccinate synthetase (ASS) and argininosuccinate lyase (ASL) (Figure 5b) [4]. These enzymes are often co-induced with NOS2 [436], and ASL facilitates formation of a multiprotein complex (including NOS2, ASS, HSP90, and CAT1) required for efficient NO production [437]. Although kidneys account for most systemic L-arginine regeneration, ~40% occurs in extrarenal tissues [438].

L-citrulline itself can be derived from multiple sources, including reversal of the arginase pathway via intermediates such as L-ornithine, as well as via enzymes such as ornithine transcarbamylase and carbamoyl phosphate synthetase, which are expressed in liver and intestinal epithelial cells (Figure 5b) [439]. Additional sources include creatine synthesis mediated by arginine:glycine amidinotransferase and degradation of methylated arginines (e.g., ADMA) by dimethylarginine dimethylaminohydrolases [438]. Both dietary intake and proteolysis contribute to the amino acid pool.

The gut microbiota further supplements host metabolism by producing L-citrulline and contributing to luminal L-ornithine availability from undigested substrates via collective biosynthetic pathways, including ornithine transcarbamylase-mediated conversion of L-ornithine to L-citrulline and microbial agmatine/polyamine metabolic networks that interconnect agmatine, putrescine, and ornithine metabolism [440].

Because ASL is essential for intracellular L-arginine regeneration, the L-citrulline–NO cycle becomes particularly important under conditions of increased metabolic demand, such as inflammation and cancer. Accordingly, ASL expression is elevated in IBD and correlates with NOS2 levels [23]. Tumors lacking ASS/ASL are L-arginine auxotrophic and sensitive to depletion [441], whereas in colorectal cancer, ASL overexpression is associated with poor prognosis [442]. ASL silencing induces G2/M arrest, promotes autophagy, and reduces NO production [442].

Clinically, patients with colorectal cancer exhibit normal systemic L-arginine but reduced L-citrulline levels [443], while L-citrulline (but not L-arginine) is decreased in active IBD, particularly in UC [39]. Notably, the impact of impaired L-citrulline–NO cycling is cell-type dependent: ASL deficiency exacerbates colitis in intestinal epithelial cells but alleviates it in immune cells, likely due to reduced NO-driven macrophage activation and M1 polarization [23].

### 6.3. Catalytic Activity

In addition to competing for L-arginine transport, ADMA, NMMA, and, to a lesser extent, SDMA are endogenous products of arginine-methylated proteins and act as competitive inhibitors of all NOS isoenzymes [434]. Under conditions of tetrahydrobiopterin deficiency, such as inflammation, these compounds may also promote NOS2 uncoupling. In this state, electron transfer is redirected from L-arginine to molecular oxygen, resulting in superoxide production and reduced NO bioavailability due to rapid consumption via peroxynitrite formation [444]. Methylarginines are generated by protein arginine methyltransferases, whose activity is increased in the intestinal mucosa of CD and UC patients. Despite this, systemic ADMA levels remain unchanged due to concurrent upregulation of dimethylarginine dimethylaminohydrolases, enzymes involved in ADMA degradation [39]. Another endogenous inhibitor is N^G^-iminoethylornithine, an amidino amino acid that, similarly to NMMA, can act not only as a competitive inhibitor but also as a mechanism-based (suicidal) inhibitor [434].

Interactions with the cytoskeleton further regulate NOS2 activity. In macrophages, hypoxia reduces NO production independently of protein expression, dimer stability, or oxygen availability, instead disrupting NOS2 interaction with α-actinin 4, an anchor protein [445]. In activated macrophages, NOS2 activity is enhanced by overexpression of Rho family GTPases, particularly Rac2. However, the underlying mechanism has not been fully elucidated [446]. Enzyme activity is also negatively regulated by caveolin-1. This interaction, common to all NOS isoenzymes, likely involves a caveolin-binding motif located near Glu^361^, a residue important for L-arginine binding [447]. Overexpression of caveolin-1 in intestinal epithelial cells (Caco-2 and HT-29) significantly reduces NOS2 catalytic activity [448].

### 6.4. Post-Translational Covalent Modifications

Although less extensively regulated by post-translational covalent modifications than other NOS isoenzymes, NOS2 activity can be modulated by phosphorylation, acylation, nitration, S-nitrosylation, and ubiquitination.

Phosphorylation has been observed to coincide with NOS2 induction in activated macrophages and is enhanced upon phosphatase inhibition, suggesting a regulatory role in catalytic activity [449]. However, its functional impact appears to be cell type-dependent, with reports of unchanged or even reduced activity in other systems [450].

Src family kinases, including Src and Fyn, phosphorylate NOS2 in cytokine-stimulated intestinal epithelial cells (e.g., DLD-1) [450,451]. This modification does not directly enhance enzymatic activity but instead alters subcellular localization, with up to 85% of phosphorylated NOS2 found in the particulate fraction [450]. Src-mediated phosphorylation also increases NOS2 protein stability and extends its half-life without affecting gene expression [451]. Tyr^151^ [450] and Tyr^1055^ [451] have been identified as key phosphorylation sites, the latter being particularly important for enzyme stability and linked to upstream EGF/EGFR signaling in cancer cells [451].

Tyrosine nitration (Y-nitration), observed in septic patients, leads to loss of NOS2 activity [414], while cysteine S-nitrosylation can further modulate enzyme function [415]. S-nitrosylation has been proposed as a signaling mechanism analogous to phosphorylation, enabling NOS2 to act as a transnitrosylating agent. For example, NOS2 can cooperate with calprotectin, forming complexes in which NOS2 donates NO and calprotectin mediates transnitrosylation of target proteins [452]. However, the broader role of S-nitrosylation as a signaling mechanism remains debated because S–NO bonds are chemically labile and readily undergo conversion into disulfide bonds [453].

Palmitoylation of NOS2 at Cys^3^ is essential for NO production, and this modification depends on neighboring residues, particularly Pro^4^ and Lys^6^ [454].

### 6.5. Subcellular Localization

To deliver NO to appropriate cellular targets while limiting collateral cytotoxicity, NOS2 localization is tightly regulated. The enzyme is partially cytosolic (diffuse pattern) but can associate with membranes or be present in the nucleus in a cell type- and context-dependent manner [448,454,455,456]. More recently, NOS2 has been shown to accumulate in perinuclear aggresomes in a time-dependent manner, revealing a previously unrecognized regulatory mechanism [457].

NOS2 trafficking is mediated by interactions with Rho GTPases, including Rac1 and Rac2, which bind the oxidoreductase domain and direct the enzyme, e.g., to phagosomes. Importantly, NOS2 is transported in an inactive form, thereby protecting cellular components from oxidative damage. This trafficking function is independent of Rho enzymatic activity [446]. Subcellular localization is also influenced by post-translational covalent modifications. Palmitoylation is required for NOS2 exit from the Golgi/trans-Golgi network; inhibition of this modification leads to accumulation of inactive enzyme within the Golgi apparatus [454]. In intestinal epithelial cells, phosphorylation promotes membrane association [450].

NOS2 has also been detected within the plasma membrane, including caveolae [448], and the apical membrane of polarized epithelial cells such as airway [458] and intestinal [459] epithelium, facilitating access of NOS2-derived NO to nearby molecular targets. In intestinal epithelial cells (DLD-1, Caco-2), most NOS2 activity is associated with the particulate fraction, where the enzyme exists almost only in its active dimeric form [459]. Reported association with cortical actin [455] is likely mediated by the scaffold protein EBP50 (‘ezrin-radixin-moesin-binding phosphoprotein 50’), which links NOS2 to submembranous structures via its PDZ domains and the C-terminal Ser-Ala-Leu motif of NOS2 [458]. In vascular smooth muscle cells, subcellular localization is dependent on δ_2_ variant of calcium/calmodulin-dependent protein kinase II (CaMKIIδ_2_). Acute and chronic CaMKIIδ_2_ activation promotes nuclear and membrane NOS2 localization, while kinase inhibition endorses cytoplasmic distribution [460].

### 6.6. Protein Turnover

NOS2 is mainly degraded via the ubiquitin–proteasome system, as shown by studies using inhibitors of proteasomal, lysosomal, and calpain-dependent pathways [461].

#### 6.6.1. Proteasomal Degradation

NOS2 undergoes Lys^48^(K48)-linked polyubiquitination, targeting it for proteasomal degradation [462]. Three major ubiquitination pathways have been described: HSP70/CHIP-mediated, ECS (‘Elongin–Cullin–SOCS-box’), and SCF (‘Skp–Cullin–F-box’) systems [463,464,465].

In the ECS pathway, NOS2 interacts with SPSB (‘SPRY domain-containing SOCS box’) proteins (SPSB1, SPSB2, and SPSB4, but not SPSB3) [464,466]. These proteins recognize the N-terminal ^23^DINNN^27^ motif of NOS2, with Asp^23^, Asn^25^, and Asn^27^ being critical for SPSB2 binding and Asn^27^ confirmed for SPSB1 and SPSB4 interactions [464,466]. Binding of SPSB1 and SPSB4 can also influence NOS2 subcellular distribution [466]. SPSB proteins have additionally been implicated in the clearance of NOS2 aggregates via the ubiquitin–proteasome system [467].

In the SCF system, NOS2 interacts with the F-box protein FBXO45, which also recognizes the ^23^DINNN^27^ motif and requires Asn^27^ for binding. FBXO45 forms an E3 ligase complex with MYCBP2 (‘Myc-binding protein 2’) and SKP1 (‘S-phase kinase-associated protein 1’) [465].

HSP90 plays a protective role by preventing NOS2 aggregation; its deficiency leads to aggregate formation and subsequent degradation via SPSB-mediated pathways of the ubiquitin–proteasome system [467].

Deubiquitination is mediated by UCH37 (UCHL5), recruited to the 26S proteasome by Rpn13 (also known as NAP110). This complex facilitates NOS2 degradation while simultaneously promoting NF-κB signaling by targeting its inhibitor IκBα for degradation, thereby enhancing *NOS2* expression [420].

Caveolin-1 further contributes to NOS2 turnover by promoting its proteasomal degradation. In human intestinal epithelial cells (particularly HT-29), caveolin-1 reduces NOS2 half-life by approximately 30% [448]. This downregulation is associated with decreased tumorigenic potential, as demonstrated in mouse models using caveolin-1-expressing HT-29 and DLD-1 cells [468].

#### 6.6.2. Sequestration in Aggresomes

Aggresomes are inclusion bodies located at the microtubule-organizing center and have been shown to regulate NOS2 availability by acting as reservoirs of catalytically inactive but potentially functional enzyme [457,469]. Following induction, NOS2 is progressively trafficked from the cytoplasm to aggresomes via dynein-dynactin-dependent retrograde transport along microtubules, such that aggresomal localization becomes increasingly prominent over time [457]. Trafficking of NOS2 to aggresomes is accompanied by loss of enzymatic activity [469]. Perinuclear accumulation of NOS2 is associated with vimentin rearrangement, resulting in formation of a cage-like structure surrounding enzyme aggregates [457].

Aggresome formation requires CHIP-dependent ubiquitination, which enables interaction between NOS2 and histone deacetylase (HDAC)-6. HDAC6, in turn, anchors NOS2 to dynein and facilitates its transport to the microtubule-organizing center, where the aggresome is formed [470].

Deposition of NOS2 in aggresomes increases in response to cellular stress such as heat shock or HSP90 depletion [457]. Moreover, it is also facilitated by NO, operating in macrophages as a negative feedback mechanism [471].

### 6.7. Implications for IBD and Intestinal Inflammation

Regulation of NOS2 extends beyond gene expression and is strongly influenced by post-translational mechanisms that determine enzyme activity, stability, and substrate availability. Several of these processes are altered in IBD, including arginine metabolism, citrulline recycling, and signaling pathways controlling NOS2 turnover, thereby affecting the magnitude and duration of mucosal NO production.

Of particular relevance to intestinal inflammation is the dysregulation of the L-citrulline-NO cycle and competing arginine-utilizing pathways, which can influence both immune-cell activation and epithelial homeostasis. Moreover, abnormalities in NOS2 stability and activity have been implicated in colitis-associated carcinogenesis. Collectively, these findings indicate that post-translational regulation is an important determinant of NOS2 function in IBD and may provide therapeutic opportunities beyond the transcriptional control of *NOS2* expression.

## 7. NOS2 Expression in Non-Immune Cells of the Bowel

Numerous cell types in the intestinal wall express NOS2. Among non-immune cells, NOS2 is constitutively expressed by intestinal epithelial cells [9,472,473] and can be induced in smooth muscle cells [474], endothelial cells [475,476], fibroblasts [477], and myofibroblasts [478]. Among immune cells, NOS2 expression has been reported in activated macrophages [439], neutrophils [479], myeloid-derived suppressor cells (MDSCs) [480], mast cells [481], dendritic cells [482], natural killer (NK) cells [483], and lymphocytes [484,485,486].

Because non-immune intestinal cell populations differ in their patterns of NOS2 expression, regulatory mechanisms, and biological functions, the consequences of NO production are highly context dependent. Understanding the cellular origin of NOS2-derived NO is therefore essential for interpreting its roles in intestinal homeostasis and IBD pathogenesis. The principal non-immune cellular sources of NOS2 in the intestine, their proposed functions, and relevance for IBD are summarized in Table 5.

### 7.1. Intestinal Epithelial Cells

#### 7.1.1. NOS2 Expression in Intestinal Epithelial Cells

Intestinal epithelial cells (IECs) express all three NOS isoenzymes, with NOS1 and NOS3 primarily supporting homeostatic functions. In contrast, epithelial NOS2 represents the major source of NOS2-derived NO in the intestine [9]. Although NOS2 is classically regarded as an inducible enzyme activated by microbial products and inflammatory cytokines, continuous exposure of IECs, particularly colonocytes, to the intestinal microbiota provides sufficient stimulation to maintain low constitutive expression [487]. *NOS2* transcripts are enriched in enterocytes and gut-associated lymphoid tissue, whereas the highest protein expression has been reported in the nasopharynx [472]. Along the gastrointestinal tract, expression is the highest in the terminal ileum and transverse colon and moderate in the sigmoid colon [488].

Most knowledge regarding epithelial NOS2 regulation is derived from studies using transformed intestinal cell lines. Basal expression and cytokine responsiveness vary considerably among models, partly due to genetic differences and methodological advances in transcript detection. Improved analytical techniques have demonstrated constitutive or inducible *NOS2* expression in several cell lines previously considered NOS2-negative, including T84 and HT-29 cells [106,132,489,490]. In general, Caco-2 and HCT-116 cells exhibit constitutive *NOS2* expression with further induction by inflammatory cytokines [39,70,491], whereas DLD-1, HCT-8R, T84, and HT-29 cells display low basal expression but marked inducibility [81,132,489,492]. In contrast, WiDr cells appear largely unresponsive to cytokine stimulation [492]. Variability in *NOS2* expression is influenced by differentiation status, passage number, genetic alterations, and the efficiency of NF-κB activation [490,491,493,494,495,496,497].

Human biopsy studies consistently demonstrate increased epithelial NOS2 expression in active IBD [24,39,43,498,499]. Recent single-cell transcriptomic analyses have further revealed that *NOS2* expression is concentrated within a specialized epithelial population termed LND (LCN2–NOS2–DUOX2) cells. These cells exhibit strong antimicrobial and immunoregulatory transcriptional programs, are rare in non-IBD mucosa, emerge in inactive CD, and expand markedly in active disease [500]. Moreover, LND-cell abundance has been linked to disease activity and responsiveness to anti-TNF therapy, highlighting their potential clinical relevance.

#### 7.1.2. Regulation of NOS2 Expression in Intestinal Epithelial Cells

NOS2 expression in IECs is regulated by a complex network of microbial, cytokine, metabolic, and stress-related signals. Among these, the strongest evidence supports central roles for the TLR/NF-κB and IFNγ/JAK/STAT1 pathways, which integrate microbial and inflammatory cues and cooperate with additional transcription factors including IRFs, AP-1, and C/EBPβ [501,502,503,504]. The magnitude of *NOS2* induction is further shaped by inhibitory pathways, such as TGFβ/SMAD, PI3K/Akt, and PPARγ signaling [505,506]. During chronic intestinal inflammation, persistent exposure of IECs to cytokines, microbial products, and oxidative stress disrupts this regulatory balance, leading to sustained NOS2 expression and increased NO production, both characteristic features of IBD mucosa (reviewed in [507]).

##### NF-κB Signaling and Microbial Sensing

Evidence from human IBD tissue, animal models, and cultured intestinal epithelial cells identifies NF-κB as a key regulator of epithelial *NOS2* expression [501,502,508,509]. Under physiological conditions, basal NF-κB activity contributes to epithelial survival, barrier integrity, and mucosal homeostasis. Consistent with this protective role, IEC-specific deletion of TAK1, NEMO (IKKγ), or IKKα/β causes spontaneous intestinal inflammation in mice [508,509].

During inflammation, however, excessive activation of NF-κB promotes sustained expression of *NOS2* and other pro-inflammatory mediators. In human IEC lines, combined stimulation with lipopolysaccharide and inflammatory cytokines markedly increases NF-κB1, NF-κB2, TNF-α, IL-1β, and NOS2 expression [502]. Similarly, constitutive activation of IKKβ in intestinal epithelial cells induces chronic NF-κB signaling, excessive NOS2 expression, and tumor formation in experimental models. Conversely, loss of the endogenous NF-κB inhibitor A20 (TNFAIP3) results in spontaneous intestinal inflammation and colorectal tumorigenesis.

Several factors further modulate NF-κB-dependent *NOS2* induction. PARP1 enhances NF-κB transcriptional activity, and its cleavage appears necessary for efficient *NOS2* transcription [510]. In contrast, the deacetylase sirtuin-1 suppresses NF-κB signaling and limits *NOS2* expression (reviewed in [511]). These observations derive primarily from experimental systems, although altered PARP1 and sirtuin-1 signaling has also been documented in human inflammatory disorders.

Damage-associated molecular patterns (DAMPs) provide another mechanism linking epithelial injury to *NOS2* induction. Prototypical alarmin, HMGB1, released from activated immune cells or damaged IECs activates scavenger receptor RAGE (‘receptor for advanced glycation end-products’) and TLR signaling, leading to NF-κB activation and NOS2 upregulation in epithelial cells [512]. Importantly, increased HMGB1 and RAGE expression has been documented in human IBD and experimental colitis, and fecal HMGB1 levels correlate with disease activity (reviewed in [513]).

##### HSP70, Short-Chain Fatty Acids, and PPARγ

Microbial metabolites and cellular stress responses provide an additional layer of NOS2 regulation. Intracellular HSP70 generally suppresses *NOS2* expression through inhibition of both canonical and non-canonical NF-κB signaling and by facilitating NOS2 degradation, whereas extracellular HSP70 acts as a danger signal and promotes NF-κB activation [514,515,516,517,518]. Elevated HSP70 expression has been observed in both human IBD and experimental colitis, where it is generally considered protective due to its ability to preserve epithelial viability and limit excessive inflammation [519,520,521].

Among microbial metabolites, butyrate exerts predominantly inhibitory effects on *NOS2* expression. Experimental studies have shown that butyrate suppresses IFNγ/STAT1, NF-κB, and C/EBPβ signaling in IECs [141,228,506,522]. However, opposing effects have occasionally been reported, indicating that its influence may depend on species, cell type, and inflammatory context [503].

PPARγ, a major target of microbiota-derived butyrate, also suppresses NF-κB, AP-1, and JAK/STAT signaling [404]. Evidence from both human studies and animal models indicates reduced epithelial PPARγ expression in UC [523,524], while PPARγ deficiency is associated with enhanced *NOS2* expression and heightened intestinal inflammation [506]. Accordingly, PPARγ agonists suppress *NOS2* expression and attenuate inflammatory signaling.

##### Cytokine-Dependent JAK/STAT Signaling

Evidence from IEC cell lines, experimental models, and human IBD tissues consistently identifies IFNγ/JAK/STAT1 signaling as a major cytokine-dependent pathway driving *NOS2* expression [503,525,526]. Following stimulation with IFNγ, STAT1 binds GAS elements within the NOS2 regulatory region and promotes transcriptional activation. In human IECs, including DLD-1 and Caco-2 cells, STAT1 activation is required for cytokine-induced *NOS2* expression, while pharmacological or genetic disruption of the pathway markedly reduces *NOS2* induction [503].

Maximal *NOS2* expression often requires cooperation between STAT1 and additional transcription factors. IL-22, which is overexpressed in inflamed IBD mucosa [525], enhances IFNγ-induced *NOS2* expression through STAT3 activation [526]. Likewise, STAT1 cooperates with IRF proteins at adjacent GAS and ISRE/IRF-E elements within the *NOS2* promoter [132]. Consequently, *NOS2* induction in IECs reflects integration of multiple cytokine-derived signals rather than activation of a single pathway.

Negative regulation is provided by the TGFβ/SMAD axis. Experimental studies have shown that TGFβ suppresses STAT1-dependent *NOS2* transcription in IECs by promoting STAT1 interaction with the inhibitory factor PIAS1 without affecting STAT1 phosphorylation or nuclear translocation [505]. This mechanism may contribute to limiting excessive epithelial NO production during mucosal restitution and resolution of inflammation.

##### MAPK and EGFR Signaling

MAPK pathways provide an important link between inflammatory cytokines, growth factors, and stress responses. Activation of ERK, JNK, and p38 kinases induces AP-1 transcription factors and contributes to *NOS2* expression in IECs [78,504,527]. Evidence for this pathway derives primarily from cell-culture studies and experimental colitis models. In particular, JNK activation correlates with increased *NOS2* expression and epithelial apoptosis, whereas inhibition of PARP1 attenuates JNK/c-Jun activation and reduces *NOS2* induction [155].

MAPKs also mediate IFNγ-dependent induction of C/EBPβ in IECs [504]. In addition to directly stimulating *NOS2* transcription, C/EBPβ promotes expression of the purinergic receptor P2Y2 [229]. Since P2Y2 is upregulated in IBD and experimental colitis [528], this pathway may amplify inflammatory responses while simultaneously participating in mucosal repair through activation of COX2/PGE_2_ and NOS/NO signaling (reviewed in [529]).

Given the established role of EGFR in promoting NOS2 expression in macrophages [530], modulation of EGFR signaling could potentially influence NOS2 expression in IECs. However, evidence for this mechanism in the intestinal epithelium remains limited. In IECs, EGFR-MAPK signaling primarily promotes mucosal integrity by enhancing epithelial survival, regeneration, and restitution following injury [531,532]. Consistent with these cytoprotective functions, the EGFR ligand HB-EGF (‘heparin-binding EGF-like growth factor’) suppresses cytokine-induced NOS2 expression at both the mRNA and protein levels in DLD-1 cells [533]. However, the relationship between EGFR signaling and NOS2 appears to be context dependent. Although excessive *NOS2* expression has been associated with intestinal pathology, including necrotizing enterocolitis [534], studies using immature human intestinal mucosa have demonstrated that NOS2 mediates beneficial effects of EGF on tissue integrity. In these models, *NOS2* expression was suppressed by indomethacin, a nonsteroidal anti-inflammatory drug known to induce necrotizing enterocolitis as an adverse effect [535]. Likewise, animal studies suggest that NOS2-derived nitric oxide contributes positively to early mucosal repair processes [536]. Consistent with these observations, EGF induces NOS2 expression in HCT-116 cells and promotes the expression of COX2 and VEGFA, pointing to a potential role in inflammation-associated angiogenesis [537].

These seemingly divergent observations likely reflect the context-dependent nature of both EGFR and NOS2 signaling. Experimental studies indicate that EGFR may regulate NOS2 through both MAPK-dependent pathways and direct transcriptional mechanisms. Although the signaling pathway underlying *NOS2* induction has not been fully characterized in IECs, studies in human breast cancer cells have demonstrated that EGF-induced *NOS2* expression depends on EGFR functioning as a transcriptional cofactor through complex formation with STAT3 [538]. Two STAT3/EGFR response elements have been identified in the *NOS2* promoter, and EGF stimulation enhances STAT3/EGFR binding at these sites, resulting in a sevenfold increase in *NOS2* expression [538]. Consistent with the potential relevance of this mechanism to the intestinal epithelium, EGF-induced nuclear translocation of EGFR has been observed in rat IECs, where impaired nuclear trafficking under heat stress is associated with reduced activation of EGFR-, STAT3-, AKT-, and ERK1/2-dependent signaling and increased apoptosis [539].

Further complexity arises from the ability of EGFR to respond to cellular stress independently of ligand binding. In IECs (SW480) and other epithelial cell lines, stress-induced activation of TAK1/p38 or TAK1/ERK signaling promotes phosphorylation of EGFR at Ser^1046/7^ or Thr^669^, respectively, resulting in receptor activation despite the absence of ligand engagement or intrinsic tyrosine kinase activity [540,541]. Importantly, the TAK1/p38/EGFR axis promotes epithelial cell survival independently of the canonical anti-apoptotic TAK1/NF-κB pathway [541]. Moreover, signaling downstream of distinct EGFR ligands may produce different biological outcomes; for example, amphiregulin-induced activation of MAPK and NF-κB has been implicated in hepatic inflammation [542]. Together, these findings suggest that EGFR may influence NOS2 expression and epithelial homeostasis through multiple interconnected and context-dependent signaling mechanisms. However, the relative importance of these pathways in human IBD remains uncertain.

##### CaSR, Wnt/β-Catenin, and RhoA Signaling

Several additional pathways modulate NOS2 expression in IECs, although evidence for their involvement derives predominantly from experimental systems.

The calcium-sensing receptor (CaSR) functions as a sensor of extracellular calcium, amino acids, and polyamines [543]. Beyond its established role in mineral homeostasis, CaSR limits intestinal inflammation and suppresses *NOS2* expression. Intestinal epithelial-specific deletion of CaSR increases *NOS2* expression, impairs barrier integrity, and accelerates experimental colitis [544]. Potential mechanisms include activation of PI3K/Akt signaling and inhibition of JNK and NF-κB pathways [545]. Nevertheless, recent studies have reported beneficial effects of pharmacological CaSR inhibition in experimental colitis [546], suggesting that its role may be more complex than previously appreciated.

Wnt/β-catenin signaling is a central regulator of intestinal stem-cell maintenance, epithelial regeneration, and mucosal homeostasis. Because IBD is characterized by recurrent cycles of epithelial injury and repair, increased activity of the Wnt/β-catenin pathway is thought to support regenerative responses in the inflamed mucosa. The pathway is positively regulated by TNFα, which enhances β-catenin transcriptional activity, but negatively regulated by IFNγ through depletion of the Wnt co-receptor LRP6 [547]. Since both cytokines are potent inducers of *NOS2*, these observations further underscore the complexity of the relationship between NOS2 and Wnt/β-catenin signaling. Mechanistically, β-catenin can directly stimulate *NOS2* transcription in IECs via TCF4 [74]. Conversely, β-catenin may suppress *NOS2* expression by interacting with the NF-κB subunit p65 and attenuating NF-κB-dependent transcription [548].

Additional complexity arises from functional interactions between β-catenin, HIF1α, and XBP1, two transcription factors implicated in *NOS2* regulation [76,224]. Beyond its role in the unfolded protein response, XBP1 regulates antimicrobial and inflammatory responses, epithelial self-renewal, and apoptosis. IEC-specific deletion of XBP1 results in spontaneous enteritis associated with impaired Paneth-cell function and reduced expression of lysozyme and α-defensins, while colonic XBP1 deficiency exacerbates dextran sulfate sodium (DSS)-induced colitis [225]. Moreover, β-catenin has been shown to cooperate with HIF1α and XBP1 to suppress hypoxia-induced transcriptional programs [76,224], suggesting another mechanism through which Wnt/β-catenin signaling may influence *NOS2* expression.

The physiological relevance of β-catenin-mediated repression of NF-κB is illustrated by studies of cadmium toxicity. Exposure to cadmium disrupts Wnt/β-catenin signaling through downregulation of Wnt ligands, receptors, and β-catenin itself, accompanied by increased expression of the pathway inhibitor GSK3β. These changes relieve β-catenin-mediated inhibition of NF-κB, resulting in increased *NOS2* expression and intestinal inflammation [549]. Collectively, these findings indicate that the impact of Wnt/β-catenin signaling on *NOS2* expression is highly context-dependent and reflects the balance between its direct transcriptional effects, interactions with NF-κB, and crosstalk with stress-response pathways.

Rho GTPases are important regulators of NOS2 expression, with RhoA exerting predominantly inhibitory effects. In both inflamed mucosa from IBD patients and experimental colitis, RhoA accumulates in the cytoplasm in an inactive state due to impaired geranylgeranylation, a prenylation-dependent modification required for membrane localization and signaling activity [550]. The importance of this pathway for intestinal homeostasis is underscored by the spontaneous enteritis observed in RhoA-deficient mice, which is accompanied by increased infiltration of CD4^+^ T cells and neutrophils as well as enhanced TNFα production [550]. Consistent with a negative regulatory role, loss of RhoA activity is associated with increased NOS2 expression. Accordingly, RhoA suppresses lipopolysaccharide- and cytokine-induced NOS2 expression in vascular smooth muscle cells [551], whereas inhibition of RhoA signaling, either directly by *Clostridium difficile* toxin B or indirectly through statin-mediated blockade of geranylgeranylation, enhances *NOS2* expression in IECs and fibroblasts [552].

Mechanistically, RhoA signaling through Rho-associated kinase (ROCK/p160ROCK) limits cytokine-induced *NOS2* expression by promoting actin polymerization and stabilization of the cytoskeleton through maintenance of filamentous (F)-actin [453]. Conversely, cytoskeletal disruption facilitates *NOS2* induction in response to inflammatory stimuli [453]. This inhibitory effect involves activation of c-Fos and c-Jun, components of the AP-1 transcription factor complex that represses *NOS2* transcription [81,527,553]. However, cytoskeleton-dependent regulation of *NOS2* is not restricted to transcriptional control, and post-transcriptional mechanisms involving modulation of mRNA stability by RNA-binding proteins such as AUF1 and HuR have also been proposed [553].

Regulation of NOS2 by prenylation pathways extends beyond RhoA. Farnesylation-dependent activation of Ras signaling has likewise been reported to suppress IL-1- and NF-κB-driven NOS2 expression in rat macrophages [554]. Together, these findings identify protein prenylation and RhoA-dependent cytoskeletal signaling as important negative regulators of NOS2 expression and suggest that defective prenylation may contribute to excessive nitric oxide production during intestinal inflammation.

Collectively, current evidence supports roles for CaSR, Wnt/β-catenin, and RhoA as modulators rather than primary drivers of NOS2 expression in intestinal epithelial cells.

##### Regulation by Immune Cell Interactions

Most mechanistic studies of NOS2 regulation have been performed using isolated epithelial-cell cultures. However, evidence from co-culture systems suggests that epithelial NOS2 expression in vivo may be influenced substantially by interactions with mucosal immune cells. Hoffman et al. [555] demonstrated that rat IECs expressed NOS2 in response to individual cytokines when co-cultured with intraepithelial lymphocytes (IELs), whereas the same stimuli were insufficient in epithelial monocultures. Induction of NOS2 required both direct cell–cell contact and IFNγ signaling and occurred in epithelial cells as well as macrophages [555]. These observations suggest that epithelial NOS2 expression within the intestinal mucosa results not only from exposure to soluble inflammatory mediators but also from continuous bidirectional communication between intestinal epithelial cells and resident immune cells. Such interactions may explain why relatively modest inflammatory stimuli can trigger substantial NOS2 expression in vivo compared with isolated cell-culture systems.

### 7.2. Stromal Cells

Compared with epithelial and immune cells, the contribution of stromal cells to IBD pathogenesis has received relatively limited attention. However, intestinal fibroblasts and myofibroblasts (IMFs) are now recognized as active regulators of mucosal homeostasis. Recent single-cell and spatial transcriptomic studies have revealed marked fibroblast heterogeneity in IBD and identified inflammation- and fibrosis-associated stromal subsets, underscoring the active role of stromal cells in disease pathogenesis rather than merely extracellular matrix production [556,557]. In addition to producing extracellular matrix components, they support epithelial renewal through secretion of Wnt ligands and antagonists of bone morphogenetic proteins, modulate immune responses through cytokine and chemokine production, and interact directly with T cells via PD-L1 and PD-L2 [558]. Increasing evidence indicates that these stromal populations actively regulate epithelial barrier integrity, intestinal immunity, and tissue regeneration rather than serving solely structural roles [557].

Evidence from human IBD tissues indicates that stromal cells acquire an activated phenotype characterized by increased proliferation, reduced migratory capacity, and enhanced extracellular matrix production [558]. Persistent activation of IMFs promotes fibrosis through excessive synthesis of collagen types I and III, secretion of TGFβ1, resistance to apoptosis, and acquisition of a contractile phenotype, ultimately contributing to remodeling and distortion of intestinal architecture [559]. Such stromal remodeling is increasingly recognized as a major driver of fibrostenotic complications in IBD [557].

Fibroblasts express all three NOS isoenzymes, whereas NOS2 is induced in response to inflammatory stimulation [477,560]. Experimental studies suggest that NOS2 induction in IMFs is a two-step process requiring prior sensitization [478]. Exposure to IFNγ enhances TNFRII expression and sensitizes cells to TNFα, whereas TNFα increases IFNγRI expression and sensitizes cells to IFNγ. Subsequent stimulation activates NF-κB, Akt, and JAK/STAT1 signaling pathways, resulting in robust NOS2 expression [478].

Evidence from cultured IMFs further indicates that IL-22 potentiates IFNγ-induced *NOS2* expression through STAT3 activation [526]. Conversely, butyrate and other HDAC inhibitors suppress IFNγ/lipopolysaccharide-induced NOS2 expression [503], while overexpression of ELK3, a transcription factor induced by TGFβ/SMAD3 signaling, also represses *NOS2* transcription [203,561].

Additional evidence from fibroblast cultures suggests that hypoxia induces NOS2 expression through NF-κB activation. In peritoneal fibroblasts, hypoxic conditions promote phosphorylation and degradation of IκBα, leading to enhanced NOS2 expression [562].

Collectively, available evidence suggests that fibroblasts and myofibroblasts may represent an underappreciated source of NO in the inflamed intestine. In addition to participating in immune signaling, these cells play central roles in tissue repair and fibrosis, implying that dysregulated NOS2 expression could influence both mucosal healing and fibrostenotic complications of IBD. However, most current mechanistic evidence derives from in vitro studies, and further investigation is required to establish the contribution of stromal NOS2 to human disease.

### 7.3. Smooth Muscle Cells

Impaired intestinal motility is a common feature of IBD and has been linked, at least in part, to excessive NO production [563]. Evidence from experimental models indicates that induction of *NOS2* in the inflamed intestine contributes to smooth muscle dysfunction. In rats, *NOS2* expression induced in the colon by lipopolysaccharide is accompanied by reduced smooth muscle contractility, an effect that can be reversed by pharmacological inhibition of NOS activity with L-NAME [564].

Studies in cultured rat intestinal smooth muscle cells have identified inflammatory cytokines as major inducers of *NOS2* expression. IL-1β stimulates *NOS2* transcription, an effect enhanced by co-stimulation with lipopolysaccharide and synergistically amplified by TNFα, whereas TGFβ1 suppresses *NOS2* induction [474]. In addition to inflammatory mediators, mechanical stress associated with intestinal distension promotes *NOS2* expression in both smooth muscle cells and macrophages, suggesting that altered gut motility and inflammation may reinforce one another through NO-dependent mechanisms [565,566].

Although the molecular basis of TGFβ-mediated NOS2 inhibition has not been specifically established in intestinal smooth muscle cells, studies in vascular smooth muscle cells suggest several plausible mechanisms. These include SMAD3-dependent interference with C/EBPβ- and NF-κB-mediated transactivation of the *NOS2* promoter [567], induction of the repressive TCF11/MafG complex [75], and downregulation of HMG-I(Y), a chromatin-associated regulator required for efficient cytokine-induced *NOS2* transcription [568]. Thus, TGFβ signaling likely represents an important negative regulator of *NOS2* expression in smooth muscle cells.

Subcellular localization may provide an additional level of regulation. In cytokine-stimulated vascular smooth muscle cells, NOS2 displays a diffuse cytoplasmic distribution but accumulates preferentially in perinuclear aggresomes, a process regulated by CaMKIIδ_2_ activity [460]. However, the relevance of this mechanism to intestinal smooth muscle cells has yet to be determined.

Compared with rodent cells, human smooth muscle cells exhibit limited *NOS2* inducibility in vitro. Evidence from primary human smooth muscle cells and endothelial cells indicates that this hyporesponsiveness is associated with epigenetic repression, including extensive H3K9 methylation, high CpG methylation within the *NOS2* promoter, and recruitment of methyl-CpG-binding proteins such as MeCP2 [244].

Collectively, available evidence, largely derived from animal models and cell-culture studies, suggests that NOS2 contributes to inflammation-associated dysmotility and may participate in the reciprocal relationship between mechanical stress and intestinal inflammation. However, the regulation and functional significance of NOS2 in human intestinal smooth muscle remain insufficiently characterized.

### 7.4. Endothelial Cells

Pathological activation of the intestinal microvasculature contributes to increased vascular permeability, leukocyte recruitment, and tissue injury in IBD. Endothelial dysfunction is closely linked to altered NO homeostasis. Compared with many other cell types, endothelial cells exhibit limited inducibility of *NOS2*, a phenomenon attributed largely to epigenetic repression, including extensive H3K9 methylation mediated by the histone methyltransferase EZH2 [244,248].

Among non-immune intestinal cell types, evidence supporting a functional role for NOS2 in human disease is arguably the strongest for intestinal microvascular endothelial cells. Studies using primary human intestinal microvascular endothelial cells (HIMECs) demonstrated that NOS2-derived NO limits leukocyte adhesion, whereas pharmacological NOS inhibition promotes endothelial activation and leukocyte binding [475]. Moreover, HIMECs isolated from patients with IBD exhibit reduced NOS2 expression [476].

Under physiological conditions, endothelial NO is produced primarily by NOS3 and plays a central role in regulating vascular tone, maintaining barrier integrity, limiting leukocyte adhesion, and supporting angiogenesis [569]. Accordingly, endothelial dysfunction in IBD has generally been attributed to reduced NOS3 activity and NOS3 uncoupling, resulting in superoxide rather than NO production [569]. In contrast, the role of NOS2 appears more complex. Experimental studies indicate that NOS2 may exert protective effects in the intestinal microvasculature despite its well-established pro-inflammatory functions in other settings. In acetic acid-induced colitis, NOS2-deficient mice developed more persistent inflammation than wild-type animals, a phenomenon associated with reduced leukocyte recruitment [22]. Consistent with these findings, Binion et al. [475] demonstrated that inhibition of NOS2 in HIMECs increased leukocyte adhesion. Moreover, NOS2 expression was found to be reduced in HIMECs isolated from patients with IBD [476].

Endothelial NOS2 expression is regulated by multiple inflammatory and environmental stimuli. Hypoxia is a potent inducer of NOS2 in microvascular endothelial cells [570]. In addition, shear stress generated by blood flow activates both NOS3 and NF-κB-dependent *NOS2* expression [571]. Pattern recognition receptors, TLRs and NOD1/2, are expressed on HIMECs, enabling them to respond to microbial products through activation of NF-κB and IRF signaling pathways (reviewed in [572]).

Evidence from endothelial cell culture studies further indicates a role for C/EBP family transcription factors in NOS2 regulation. Several degenerate C/EBP-binding sites within the proximal *NOS2* promoter contribute to both basal and IL-1β-induced transcription. The transcriptionally active LAP isoform of C/EBPβ enhances *NOS2* expression, whereas the inhibitory LIP isoform suppresses it, although the effects of C/EBPβ appear weaker than those of C/EBPα and C/EBPδ [95]. Endothelial cells also express high levels of ELK3, a TGFβ-responsive transcriptional repressor that negatively regulates *NOS2* expression [573].

Collectively, the available evidence suggests that endothelial NOS2 differs from immune-cell NOS2 in that it may serve predominantly protective functions by limiting leukocyte adhesion and supporting vascular homeostasis. Notably, this conclusion is supported not only by experimental models but also by studies using primary human intestinal endothelial cells [475,476].

### 7.5. Enteric Nervous System (ENS)

The enteric nervous system (ENS), composed of enteric neurons and enteric glial cells (EGCs), is an important regulator of intestinal barrier function, secretion, and motility. In healthy tissue, NO produced primarily by NOS1 contributes to normal neuromuscular and epithelial signaling. During intestinal inflammation, however, NOS2 expression is induced within the ENS and becomes an additional source of NO [574].

Evidence from experimental colitis indicates that ENS-derived NO contributes to epithelial dysfunction. MacEachern et al. [574] demonstrated that NO produced by NOS2-expressing EGCs, rather than enteric neurons, is responsible for impaired epithelial ion transport during colitis. These findings suggest that glial NOS2 may contribute to barrier dysfunction and altered intestinal physiology in inflamed mucosa.

Communication between the ENS and intestinal epithelium is mediated, in part, by vasoactive intestinal peptide (VIP), a neuropeptide with important regulatory effects on epithelial secretion and mucosal homeostasis. IBD-associated inflammation has been linked to altered VIP signaling and dysregulated NO production within the ENS. However, data regarding VIP expression remain inconsistent. Soufflet et al. [575] observed higher VIP expression in colonic biopsies from patients with UC than from patients with CD, with increased expression particularly evident in inflamed UC mucosa. Moreover, supernatants derived from UC biopsies increased VIP expression in primary ENS cultures, whereas CD-derived supernatants had a weaker effect. Further analyses identified IL-6 as a mediator capable of suppressing VIP expression in ENS cultures exposed to supernatants from inflamed CD tissue [575].

Collectively, the available evidence suggests that the ENS, particularly enteric glial cells, represents an additional source of NOS2-derived NO during intestinal inflammation. Although enteric glial activation and broader ENS remodeling are increasingly recognized features of human IBD, most mechanistic evidence implicating NOS2 originates from animal and ex vivo studies. Consequently, the quantitative contribution of ENS-derived NOS2 to intestinal inflammation in patients remains uncertain [576,577].

### 7.6. Extracellular Regulation of NOS2

Extracellular matrix (ECM) remodeling represents an additional mechanism regulating NOS2 expression. Beyond its structural role, the ECM serves as a reservoir of biologically active molecules and a source of damage-associated molecular patterns (DAMPs) generated during tissue injury. Increasing evidence indicates that ECM-derived signals actively shape inflammatory and reparative responses in IBD rather than merely reflecting tissue damage [578]. In the intestine, human studies have documented profound alterations in ECM composition and progressive fibrosis in chronically inflamed tissues, suggesting that extracellular signals may contribute to NOS2 dysregulation during IBD progression [579].

One of the best-characterized examples is provided by hyaluronan (HA). While high-molecular-weight HA is generally associated with tissue homeostasis, HA fragments generated during inflammation and tissue damage act as endogenous danger signals. Mechanistic studies in cultured cells and experimental animal models have demonstrated that low-molecular-weight HA activates TLR4-dependent signaling, resulting in NF-κB activation and induction of inflammatory genes, including *NOS2* [580]. Likewise, HA fragments engage TLR2- and TLR4-mediated pathways, establishing a direct link between ECM degradation and innate inflammatory responses [581]. In contrast, observational studies in human IBD tissues have shown accumulation of HA-rich matrices in both CD and UC [582], suggesting that ECM turnover may contribute to local NOS2 regulation during chronic intestinal inflammation.

Another ECM-derived mechanism has recently been described for the large chondroitin sulfate proteoglycan versican. Human studies have identified increased versican deposition in inflamed intestinal tissues and areas of tissue remodeling, where it has been associated with leukocyte recruitment and chronic inflammation (reviewed in [583]). In turn, mouse models and complementary mechanistic experiments demonstrated that versican accumulation induces NOS2 expression through AKT signaling and promotes aortic disease pathogenesis [584]. Specifically, versican-dependent activation of AKT increased NOS2 expression in the vascular wall, whereas genetic or pharmacological disruption of this pathway attenuated disease severity. Although a comparable versican-AKT-NOS2 axis has not yet been demonstrated in the intestine, these findings raise the possibility that fibrosis-associated ECM remodeling may contribute to NOS2 dysregulation in IBD.

ECM remodeling may also suppress NOS2 expression through regulation of transforming growth factor-β (TGFβ) bioavailability. TGFβ is stored in the ECM in latent complexes associated with latent TGFβ-binding proteins and can be released through proteolytic remodeling or integrin-dependent activation (reviewed in [585]). In cultured vascular smooth muscle cells, Finder et al. demonstrated that TGFβ suppresses cytokine-induced NOS2 expression and nitric oxide production by reducing NOS2 mRNA and protein levels [586]. Similar inhibitory effects have subsequently been reported in other experimental systems, supporting a broader role for TGFβ as a negative regulator of inflammatory nitric oxide synthesis.

Collectively, these findings indicate that ECM remodeling can regulate NOS2 expression through multiple mechanisms. Degradation products such as HA fragments and accumulation of proteoglycans such as versican promote inflammatory signaling and NOS2 induction, whereas activation of TGFβ exerts opposing effects. Notably, most mechanistic evidence linking ECM remodeling to NOS2 regulation derives from cell culture systems and animal models, whereas direct evidence connecting these pathways to NOS2 expression in human IBD tissues remains limited. Nevertheless, the available data suggest that ECM remodeling may represent an important interface between intestinal inflammation, fibrosis, and nitric oxide production.

## 8. NOS2 Expression in Gut Immune Cells

Immune cells represent the principal source of NOS2-derived NO in the intestinal mucosa and play central roles in host defense, maintenance of immune homeostasis, and the pathogenesis of IBD. Among them, macrophages are the best-characterized NOS2-expressing population, but increasing evidence indicates that dendritic cells, lymphocytes, neutrophils, mast cells, and myeloid-derived suppressor cells also contribute to intestinal NO homeostasis. Importantly, the consequences of NOS2 expression vary considerably among immune-cell populations owing to differences in activation stimuli, downstream effector functions, and local microenvironmental cues. Consequently, NOS2-derived NO may exert either pro-inflammatory or immunoregulatory effects depending on the cellular source and disease context. The principal immune-cell sources of NOS2 in the intestine, their proposed functions, and relevance to IBD are summarized in Table 6.

### 8.1. Macrophages

#### 8.1.1. NOS2 Expression and Macrophage Polarization

Macrophages are the principal innate immune cells responsible for intestinal surveillance and host defense [587]. NOS2 was first identified in activated macrophages and remains a defining marker of classically activated (M1) macrophages, which promote antimicrobial, pro-inflammatory, and antitumor responses. In contrast, alternatively activated (M2) macrophages preferentially metabolize L-arginine through arginase 1, generating ornithine and urea and supporting resolution of inflammation, tissue repair, and fibrosis [439].

Human IBD tissues display increased numbers of NOS2-expressing macrophages together with enrichment of M1-associated transcriptional programs. However, macrophage phenotypes in vivo rarely conform to the classical M1/M2 paradigm. Recent single-cell and spatial transcriptomic studies have further revealed substantial macrophage heterogeneity in IBD, identifying multiple resident, inflammatory, and transitional macrophage states that coexist within inflamed lesions. Rather than representing discrete M1 and M2 populations, intestinal macrophages appear to occupy a continuum of activation states shaped by local microenvironmental cues [498,588]. Particularly in CD, intestinal macrophages frequently exhibit mixed inflammatory and reparative signatures, reflecting the complex microenvironment of chronically inflamed tissue and their involvement in both inflammation and fibrotic remodeling [589]. Consequently, regulation of NOS2 expression has emerged as a key determinant of macrophage function in IBD.

#### 8.1.2. Pro-Inflammatory Pathways Driving NOS2 Expression

The strongest evidence supports TLR/NF-κB and IFNγ/JAK/STAT1 signaling as the principal pathways responsible for *NOS2* induction in macrophages [590,591]. These pathways cooperate rather than operate independently, as inhibition of NF-κB abolishes *NOS2* induction even in the presence of IFNγ [132]. In addition, ERK1/2, p38, and JNK MAPK pathways contribute to *NOS2* expression through activation of AP-1 transcription factors [592,593].

Purinergic signaling represents an important amplifier of macrophage activation. ATP stimulation of the P2X7 receptor activates NF-κB, ERK1/2, and the NLRP3 inflammasome, resulting in enhanced NOS2 expression [594]. Notably, P2X7R expression is increased in macrophages and dendritic cells within inflamed IBD mucosa, while pharmacological blockade of the pathway attenuates experimental colitis [594,595]. By contrast, adenosine signaling through A1 and A3 receptors suppresses NOS2 expression and exerts anti-inflammatory effects, in part through inhibition of p38 and ERK activation [596,597]. Additional crosstalk exists between purinergic and prostaglandin signaling, as inhibition of COX2/PGE_2_ signaling attenuates UTP/P2Y-mediated NOS2 induction in macrophages primed by lipopolysaccharide [598].

An additional layer of regulation involves the macrophage-inducible C-type lectin receptor (Mincle). In response to mycobacterial products or danger-associated signals, Mincle activates p38 MAPK, promotes eIF5A hypusination, and facilitates nuclear export of NOS2 transcripts [599]. The resulting NO production contributes to the resolution phase of inflammation by suppressing NLRP3 inflammasome activity through S-nitrosylation, establishing a negative-feedback circuit between NOS2 and inflammasome signaling [599].

Additional reinforcement of NOS2 expression is provided by C/EBPβ, which cooperates with NF-κB to activate the *NOS2* promoter. C/EBPβ is induced by lipopolysaccharide, TNFα, and IFNγ and contributes to M1 polarization, although evidence suggests that its role may be particularly important during the early phase of inflammatory activation [600,601,602]. The upstream regulator Egr2 further promotes *NOS2* expression by inducing C/EBPβ in lipopolysaccharide- and TNFα-stimulated macrophages [600]. Notably, C/EBPβ also participates in the regulation of *ARG1*, encoding arginase-1, and other M2-associated genes, whereas its inhibitory LIP isoform contributes to alternative macrophage polarization [602,603].

EGFR has emerged as an important regulator of macrophage activation and function. In macrophages stimulated by bacterial infection, EGFR signaling promotes production of pro-inflammatory cytokines, enhances antimicrobial activity, and facilitates bacterial clearance [530]. These effects are associated with activation of NF-κB-dependent transcriptional programs, including induction of *NOS2*. Accordingly, NF-κB activation downstream of EGFR signaling represents another pro-inflammatory pathway relevant to intestinal disease. In macrophages, EGFR activation promotes nuclear translocation of RELA/p65 and induction of *NOS2* expression and has been implicated in colitis-associated carcinogenesis [604].

Hypoxia, a hallmark of the inflamed intestinal mucosa, is another important regulator of macrophage NOS2 expression. HIF signaling not only adapts macrophages to reduced oxygen availability but also governs their functional polarization. HIF1α, preferentially induced by Th1 cytokines, promotes M1 polarization and stimulates *NOS2* expression, whereas HIF2α, induced by Th2 cytokines, enhances *ARG1* expression and favors M2 differentiation [605,606,607]. By controlling the activity of these competing arginine-catabolizing enzymes, HIF proteins integrate metabolic adaptation with inflammatory and tissue-repair responses. The reciprocal functions of HIF1α and HIF2α exemplify the extensive network of competing and counter-regulatory pathways that govern macrophage activation and prevent excessive nitric oxide production. For example, the TNFα/NF-κB axis promotes M1 polarization while simultaneously repressing M2-associated markers, including *ARG1*, thereby indirectly favoring *NOS2* expression and pro-inflammatory macrophage functions [608,609].

#### 8.1.3. Negative Regulation of NOS2

Because excessive NO production may cause tissue injury, several counter-regulatory pathways limit NOS2 expression in macrophages.

Among anti-inflammatory pathways, IL-10 and TGFβ are particularly important. IL-10 suppresses *NOS2* through STAT3-dependent mechanisms, whereas TGFβ antagonizes IFNγ signaling by preventing STAT1 activation [610,611]. Mechanistically, TGFβ receptor 1 directly interacts with IFNγ receptor 1 and phosphorylates it on serine residues, thereby impairing downstream STAT1 signaling [610]. TGFβ signaling is mediated primarily through SMAD2 and SMAD3, as combined deficiency of these mediators leads to exaggerated *NOS2* induction and reduced responsiveness to TGFβ [237]. Conversely, IFNγ induces SMAD7, which antagonizes TGFβ/SMAD signaling and establishes a reciprocal regulatory loop controlling macrophage activation [612,613]. In addition to suppressing IFNγ signaling, TGFβ exerts multiple post-transcriptional effects on NOS2 expression. Studies in activated macrophages demonstrated that TGFβ accelerates *NOS2* mRNA decay, inhibits translation of *NOS2* transcripts, and limits accumulation of NOS2 protein, thereby suppressing NO production even when transcriptional regulation is only modestly affected [614].

PI3K/Akt signaling exerts predominantly inhibitory effects on the TLR/NF-κB pathway and macrophage activation [615], although its influence is isoform-dependent. Akt1 suppresses *NOS2* expression and M1 polarization, whereas Akt2 promotes inflammatory activation. Consistent with these observations, Akt1-deficient mice develop more severe dextran sulfate sodium (DSS)-induced colitis accompanied by increased *NOS2* expression [616]. However, the role of Akt signaling appears context-dependent, as Akt2-deficient mice display impaired control of *Salmonella enterica* infection and develop more severe intestinal inflammation [617]. The anti-inflammatory actions of Akt1 involve inhibition of GSK3β, a positive regulator of NF-κB-dependent *NOS2* transcription [618,619,620,621]. Akt1 signaling additionally induces IRAK-M, which dampens TLR- and IL-1-mediated responses [622], and may suppress *NOS2* transcription by promoting processing of the NF-κB precursor p105 into transcriptionally repressive p50/p50 homodimers capable of recruiting HDAC1 to target promoters [211,212]. Although p110δ has been proposed to promote M1 polarization, stabilization of p110δ transcripts activates PI3K/Akt signaling and suppresses NF-κB-dependent *NOS2* expression, contributing to FAM76B-mediated protection against experimental intestinal inflammation [623].

Peroxisome proliferator-activated receptors (PPARs) provide an additional mechanism limiting NOS2 expression. The cyclooxygenase-derived prostaglandin metabolite 15-deoxy-Δ^12,14-prostaglandin J_2_ (15d-PGJ_2_) suppresses *NOS2* induction by interfering with NF-κB-, AP-1-, and STAT1-dependent transcriptional activation. These effects are mediated in part through activation of PPARγ, which competes with pro-inflammatory transcription factors for limiting amounts of transcriptional co-activators and thereby inhibits IFNγ-driven *NOS2* expression [624]. However, 15d-PGJ_2_ can also repress *NOS2* independently of PPARγ by inducing reactive oxygen species that subsequently impair JAK2/STAT signaling [625]. Similar anti-inflammatory effects have been described for PPARα activation, although in this case suppression of NOS2 appears to result primarily from enhanced proteasomal degradation of NOS2 protein rather than inhibition of transcription [626]. The relevance of this pathway to intestinal inflammation is underscored by evidence that PPARγ expression is reduced in UC and that pharmacological activation of PPARγ attenuates experimental colitis and contributes to the therapeutic effects of 5-aminosalicylic acid [627,628]. These observations suggest that impaired PPARγ signaling may facilitate persistent NF-κB- and STAT1-dependent NOS2 expression in the inflamed intestinal mucosa.

Beyond direct transcriptional regulation, NOS2 expression is also constrained by hormonal signaling pathways. Atrial natriuretic peptide (ANP) exerts inhibitory effects on the NOS2/NO pathway through several complementary mechanisms. It suppresses NOS2 expression through activation of NPR-A-dependent cGMP signaling. Besides inhibiting NF-κB- and AP-1-dependent transcription, ANP destabilizes *NOS2* mRNA and limits substrate availability by reducing CAT-2B-mediated arginine uptake, thereby restricting NO production at multiple levels [629,630].

#### 8.1.4. Metabolic and Environmental Regulation

Macrophage NOS2 expression is highly sensitive to metabolic and environmental cues present within inflamed tissues. Changes in nutrient availability, microbial metabolites, cellular stress responses, and innate immune sensing pathways can substantially alter macrophage polarization and thereby influence the magnitude and duration of NO production. In the intestinal mucosa, where macrophages are continuously exposed to dietary components, microbial products, and fluctuating oxygen levels, these mechanisms are particularly relevant to IBD pathogenesis and resolution [631].

Evidence from both human and experimental studies supports an inhibitory role of butyrate on macrophage NOS2 expression. Produced by bacterial fermentation of dietary fiber, butyrate suppresses inflammatory activation of intestinal macrophages through multiple complementary mechanisms. In lamina propria macrophages isolated from patients with UC, butyrate inhibits NF-κB activation and reduces inflammatory mediator production, including NO, indicating direct suppression of inflammatory signaling within the diseased intestinal mucosa [632]. Mechanistically, subsequent studies in murine intestinal macrophages demonstrated that butyrate promotes a homeostatic, hyporesponsive phenotype through inhibition of histone deacetylases (HDACs), resulting in reduced expression of *NOS2* and other pro-inflammatory genes [252]. Together, these findings suggest that butyrate constrains the NOS2/NO axis through both acute inhibition of NF-κB-dependent activation and longer-term epigenetic reprogramming of intestinal macrophages. Given that depletion of butyrate-producing bacteria is a common feature of IBD-associated dysbiosis [633], loss of this physiological inhibitory pathway may contribute to sustained *NOS2* expression and inflammatory macrophage activation within the intestinal mucosa.

Iron metabolism constitutes another important regulator of macrophage NOS2 expression. Early studies demonstrated that intracellular iron availability directly influences inducible NO synthesis, with iron loading suppressing cytokine-induced *NOS2* expression and NO production, whereas iron depletion had the opposite effect. Mechanistically, iron inhibits *NOS2* transcription through modulation of inflammatory transcriptional pathways, including NF-IL6/C/EBPβ-dependent signaling, thereby limiting macrophage NO generation [634,635]. However, subsequent studies revealed a more complex relationship between iron metabolism and macrophage activation. Classically activated M1 macrophages acquire an iron-retention phenotype characterized by increased ferritin expression and reduced ferroportin-mediated iron export, whereas alternatively activated M2 macrophages preferentially release iron [636]. Notably, M1 macrophages are also distinguished by high NOS2 expression, a canonical marker of classical activation [439]. This apparent discrepancy suggests that the effects of iron on NOS2 expression are strongly influenced by the broader activation state of the macrophage and the surrounding inflammatory milieu. Further studies are required to define the precise mechanisms through which iron metabolism shapes macrophage NO production.

Dietary and metabolic stress can further amplify NOS2 expression. The saturated fatty acid palmitate promotes M1 polarization through induction of ER stress, particularly activation of the PERK/eIF2α branch of the unfolded protein response. This pathway enhances JAK2/STAT1 and TLR4 signaling while suppressing STAT6-dependent M2 polarization, thereby favoring NOS2 induction and inflammatory macrophage activation [637]. These findings may be particularly relevant to IBD, where persistent ER stress has been implicated in disease pathogenesis and where dietary factors associated with Westernized lifestyles are thought to contribute to chronic intestinal inflammation [638]. Consistent with this concept, genetic variation in XBP1, a key regulator of the unfolded protein response, is associated with susceptibility to both CD and UC, while defective ER stress signaling promotes intestinal inflammation [225]. Thus, ER stress-dependent macrophage activation may represent a mechanism linking dietary and metabolic disturbances within the intestinal microenvironment to sustained NOS2 expression and inflammatory NO production.

Innate danger-sensing pathways also link tissue injury to NOS2 regulation. Activation of the cGAS-STING axis (‘Cyclic GMP-AMP synthase-stimulator of interferon genes’) promotes mitochondrial production of reactive oxygen species, metabolic reprogramming, and intracellular accumulation of succinate, a tricarboxylic acid cycle intermediate that functions as a pro-inflammatory signaling metabolite. Elevated succinate stabilizes HIF1α, thereby reinforcing inflammatory macrophage activation and establishing a metabolic state that favors NOS2 expression and M1 polarization [639,640,641]. The relevance of this pathway to intestinal inflammation is supported by reports of altered cGAS-STING signaling in both experimental models and human IBD, as well as observations of increased succinate levels in inflamed intestinal tissues. These findings suggest that STING-dependent metabolic reprogramming may contribute to sustained macrophage activation and persistent NOS2-dependent NO production within the inflamed intestinal mucosa [639,640].

#### 8.1.5. Neuroimmune Regulation of Macrophage NOS2

The nervous system provides an additional layer of control over macrophage activation. Several neuropeptides, including vasoactive intestinal peptide (VIP), pituitary adenylate cyclase-activating peptide (PACAP), α-melanocyte-stimulating hormone (α-MSH), urocortin, adrenomedullin, cortistatin, and ghrelin, suppress NOS2 expression and M1 polarization while promoting anti-inflammatory macrophage programs [642,643,644,645,646].

Among these, VIP is the best-characterized regulator. Acting through VPAC1 and VPAC2 receptors, VIP activates adenylate cyclase-dependent cAMP signaling, resulting in PKA- and CREB-mediated transcriptional responses that oppose classical macrophage activation. VIP inhibits key pathways required for NOS2 induction, including IFNγ/JAK/STAT1, NF-κB, AP-1, and MAPK signaling, thereby reducing transcription of *NOS2* and other pro-inflammatory genes. At the same time, VIP enhances the production of IL-10 and TGFβ and promotes the acquisition of a regulatory or M2-like phenotype. Notably, VIP does not merely suppress individual inflammatory mediators but interferes with the establishment of the broader M1 transcriptional program, leading to coordinated reductions in NOS2, TNF, IL-6, IL-12, and costimulatory molecule expression [642,643,644,645]. Consistent with these actions, VIP attenuates activation of tumor-associated macrophages and limits inflammatory NO production in diverse pathological settings. PACAP exerts largely analogous effects through shared VPAC receptors and cAMP-dependent signaling mechanisms. Other neuropeptides, including α-MSH, urocortin, adrenomedullin, cortistatin, and ghrelin, likewise converge on NF-κB- and MAPK-regulated inflammatory pathways, suppressing *NOS2* expression while favoring anti-inflammatory and tissue-repair responses [642,643,644,645,646].

#### 8.1.6. Additional Regulatory Mechanisms

Beyond the canonical cytokine-, TLR-, and metabolic pathways, several intracellular signaling and transcriptional regulators contribute to fine-tuning macrophage NOS2 expression. These molecules do not typically initiate *NOS2* induction but instead modulate the magnitude, duration, and transcriptional efficiency of inflammatory responses.

Among these, poly(ADP-ribose) polymerase 1 (PARP1) acts as an important positive regulator of *NOS2* transcription. PARP1 functions as a transcriptional co-activator of NF-κB and is required for maximal *NOS2* induction in response to inflammatory stimuli, including lipopolysaccharide and TNFα [250,510,647,648,649]. Mechanistically, PARP1 interacts directly with NF-κB at the *NOS2* promoter, and its transcriptional activity is enhanced by acetylation mediated by CBP/p300, whereas deacetylation by HDAC1-3 diminishes its co-activator function [649]. In addition to NF-κB, PARP1 may cooperate with AP-1, Oct-1, and STAT family transcription factors, placing it at the intersection of multiple inflammatory signaling pathways [155,649,650]. Although its role in macrophage NOS2 regulation during IBD has not been examined directly, studies in experimental colitis have implicated PARP activation in intestinal inflammation, and genetic or pharmacological inhibition of PARP attenuates disease severity and reduces expression of inflammatory mediators, including NOS2 [155,651]. These findings suggest that PARP1 may contribute to sustained NF-κB-dependent *NOS2* expression in the inflamed intestinal mucosa.

Signal integration upstream of NF-κB and MAPK activation is further provided by TAK1 ‘transforming growth factor-β-activated kinase 1’). As a central mediator of signaling downstream of TLRs and TNF and cytokine receptors, TAK1 coordinates multiple pathways required for inflammatory macrophage activation. Its activity is enhanced by TIGAR (‘TP53-induced glycolysis and apoptosis regulator’) and suppressed by kinase WNK1 (‘With-No-Lysine [K]’), leading, respectively, to increased and decreased *NOS2* expression [652,653]. Given the importance of TLR- and cytokine-driven macrophage activation in intestinal inflammation, modulation of TAK1 activity may substantially influence the intensity of *NOS2* induction within the inflamed mucosa.

Additional transcriptional regulation is provided by members of the ETS and Krüppel-like factor families. KLF4 has been reported to promote *NOS2* transcription through cooperation with RelA/NF-κB, whereas ELK3 acts as a transcriptional repressor and mediates TGFβ-dependent suppression of *NOS2* expression [170,203]. These findings further illustrate how inflammatory and anti-inflammatory signals converge at the level of the *NOS2* promoter to shape macrophage activation states.

Intracellular calcium signaling also contributes to the regulation of NO production. Activation of the calcium-calcineurin-NFAT pathway has been implicated in *NOS2* transcription in stimulated macrophages, providing an additional mechanism through which environmental and receptor-mediated signals can influence inflammatory NO synthesis [654]. Although the role of NFAT-dependent *NOS2* regulation in intestinal macrophages remains incompletely defined, calcium signaling is increasingly recognized as an important component of innate immune activation within the intestinal microenvironment.

Collectively, current evidence suggests that macrophage NOS2 expression is governed less by individual mediators than by the balance between pro-inflammatory pathways centered on TLR/NF-κB and IFNγ/STAT1 signaling and counter-regulatory mechanisms driven by IL-10, TGFβ, PI3K/Akt, and neuroimmune networks. Particularly important recent advances have highlighted the role of tissue-specific factors, including microbiota-derived metabolites, ER stress responses, hypoxia, iron homeostasis, and immunometabolic reprogramming, in shaping NOS2 expression within the intestinal mucosa. The prominence of these pathways in both experimental models and human IBD suggests that local metabolic and environmental cues may be as important as classical cytokine signaling in sustaining NOS2-dependent inflammation. Thus, NOS2 emerges not only as an effector molecule but also as a sensitive readout of the immune-metabolic state of intestinal macrophages.

### 8.2. Dendritic Cells

Dendritic cells (DCs) are professional antigen-presenting cells that play a central role in intestinal immune homeostasis by discriminating between harmless and pathogenic antigens and directing T-cell differentiation toward tolerance or effector responses [655]. Human IBD is associated with alterations in populations of dendritic cells. Inflamed intestinal tissue and peripheral blood exhibit increased numbers of plasmacytoid dendritic cells (pDCs), characterized by enhanced production of TNFα, IL-6, and IL-8, whereas patients in remission show reduced numbers of myeloid dendritic cells (mDCs) [655,656].

Dendritic cells express both NOS1 and NOS2, but NOS2-derived NO appears to be particularly important in shaping the function of dendritic cells and downstream immune responses. Evidence from experimental colitis models indicates that NOS2 promotes differentiation toward a regulatory rather than pro-inflammatory phenotype. NOS2-deficient mice infected with *Citrobacter rodentium* develop more severe colitis accompanied by increased numbers of effector dendritic cells producing TNFα, IL-6, and IL-12. Moreover, NOS2-derived NO is required for suppression of CD4^+^ T-cell activation, while pharmacological inhibition of NOS2 promotes acquisition of an effector DCs phenotype [482]. Collectively, these findings suggest a predominantly protective role for DCs-derived NO in intestinal inflammation.

Similar to macrophages, dendritic cells induce *NOS2* expression in response to activation of pattern-recognition receptors (PRRs), including TLRs, NOD receptors, and C-type lectin receptors (CLRs) [657,658,659,660]. In particular, NOD2 activation promotes differentiation toward a highly bactericidal phenotype characterized by enhanced NOS2 expression and NO production [658]. The relevance of this pathway to IBD is underscored by the strong association between NOD2 polymorphisms and susceptibility to CD [87,119], suggesting that impaired NOD2-dependent induction of *NOS2* may contribute to defective host–microbe interactions in genetically susceptible individuals. PRR signaling also induces several microRNAs involved in post-transcriptional regulation of *NOS2*. NOD2 activation upregulates the miR-29 family [340], whereas TLR stimulation induces miR-155 and miR-146 expression [661], all of which have been implicated in the regulation of NOS2 synthesis. Emerging evidence further indicates that microbiota-derived signals can modulate miRNA networks in human dendritic cells, raising the possibility that intestinal dysbiosis may influence NOS2 expression indirectly through post-transcriptional mechanisms [661].

Beyond its antimicrobial functions, NOS2 also contributes to metabolic regulation of DCs activity. Studies in experimental systems indicate that NO generated by NOS2 inhibits mitochondrial oxidative phosphorylation and promotes glycolytic reprogramming of activated dendritic cells, thereby influencing cytokine production, antigen presentation, and T-cell priming [662]. These observations suggest that NOS2 functions not only as an effector molecule downstream of innate immune activation but also as a regulator of DCs differentiation and immune function.

Available evidence, derived predominantly from murine and in vitro studies, suggests that NOS2 exerts largely immunoregulatory functions in dendritic cells. By limiting effector DCs differentiation, shaping cellular metabolism, and restraining T-cell activation, DCs-derived NO may contribute to maintenance of intestinal immune tolerance and protection against excessive inflammation. Recent advances in single-cell technologies have considerably refined our understanding of intestinal conventional dendritic-cell subsets and their interactions with T cells and the microbiota [663]. Nevertheless, the contribution of specific NOS2-expressing DCs populations to human IBD remains poorly defined. Future studies combining single-cell transcriptomics with spatial and functional analyses will be required to determine whether the regulatory functions attributed to NOS2 in experimental models are conserved in human intestinal inflammation.

### 8.3. T-Cells

T lymphocytes are central orchestrators of intestinal immunity and play critical roles in both the initiation and resolution of mucosal inflammation. Dysregulated activation of effector T-cell subsets, particularly Th1 and Th17 cells, together with impaired regulatory T-cell (Treg) function, is a hallmark of IBD [484,485]. Although T cells are not considered major sources of NO when compared with macrophages, NOS2 expression has been detected in activated γδ intraepithelial lymphocytes (IELs), αβ T cells, B cells, and plasma cells, where NO exerts both immunoregulatory and pro-survival functions [484,485]. Compared with macrophages, however, direct evidence for NOS2 expression and function in human intestinal T-cell subsets remains limited. Most mechanistic insights derive from murine or in vitro studies of γδ T cells, plasma cells, and specialized T-cell populations. Consequently, the physiological significance of T cell-derived NO in human intestinal inflammation remains incompletely understood.

Among the pathways implicated in T-cell NOS2 regulation, IFNγ-dependent signaling appears to be the best characterized. IFNγ induces both *NOS2* and *SMAD7*, an endogenous inhibitor of TGFβ signaling [613]. By interfering with SMAD2/3 activation, SMAD7 promotes effector T-cell responses and antagonizes the suppressive effects of TGFβ. Conversely, TGFβ inhibits IFNγ-dependent STAT1 activation through MEK/ERK signaling, thereby limiting NOS2 expression and inflammatory responses [613]. Dysregulation of this reciprocal IFNγ-SMAD7-TGFβ axis has been implicated in persistent activation of mucosal T cells and contributes to the imbalance between effector and regulatory T-cell populations characteristic of both CD and UC. Given the established role of SMAD7 overexpression in human IBD [238], this pathway represents one of the strongest links between NOS2 regulation and T-cell-mediated intestinal inflammation.

Among intestinal lymphocytes, γδ T cells are of particular interest. These cells constitute a major proportion of IELs and display characteristics of both innate and adaptive immunity [664,665]. Studies in peripheral γδ T cells have demonstrated inducible NOS2 expression and revealed a role for NOS2-derived NO in supporting cellular proliferation and glycolytic metabolism [484]. During intestinal injury, γδ T cells accumulate at sites of epithelial damage and promote tissue repair through the production of keratinocyte growth factor (KGF), TGFβ, and IL-22 [664]. Experimental depletion of γδ T cells exacerbates dextran sulfate sodium (DSS)-induced colitis and delays mucosal healing, whereas their reconstitution ameliorates disease severity [664]. Retinoic acid produced by mucosal dendritic cells promotes IL-22 production by γδ T cells and innate lymphoid cells, thereby enhancing epithelial regeneration and limiting bacterial translocation [664]. Thus, despite their capacity to produce IFNγ and other pro-inflammatory mediators, γδ T cells also exert important protective functions in the intestinal mucosa. These observations suggest that NOS2 expression in γδ T cells may participate in specialized programs integrating epithelial repair, metabolism, and immune surveillance.

Hypoxia-related signaling provides another link between T-cell differentiation and NOS2 expression. HIF1α promotes differentiation of Th9 cells by directly transactivating both *IL9* and *NOS2* promoters. In this context, amphiregulin/EGFR signaling enhances HIF1α activity, whereas disruption of either pathway impairs Th9-cell differentiation and effector function [666]. These findings identify *NOS2* as a component of a specialized HIF1α-dependent transcriptional program and suggest that NO may contribute to T-cell-mediated immunity beyond the classical Th1 and Th17 paradigms.

Several additional signaling and transcriptional regulators may influence NOS2 expression in T cells, although direct evidence remains limited. TAK1 is a central mediator of T-cell receptor-dependent NF-κB and JNK activation and is essential for intestinal immune homeostasis. Mice with T cell-specific TAK1 deficiency develop spontaneous colitis accompanied by profound disturbances in effector and regulatory T-cell populations [667]. Given its critical role in NF-κB signaling, TAK1 may contribute to *NOS2* regulation in activated T cells, although this possibility has not been directly investigated. Similarly, members of the Krüppel-like factor (KLF) family may link *NOS2* regulation to T-cell polarization. KLF6 activates the *NOS2* promoter in lymphoid and myeloid cells and is overexpressed in inflamed intestinal tissues from IBD patients, where it promotes NF-κB-dependent inflammatory responses [84,172]. KLF4, another IFNγ-responsive transcription factor, promotes Th17-cell differentiation through direct activation of the IL17A promoter [668,669]. Because both KLF6 and KLF4 are associated with pro-inflammatory signaling pathways that overlap with known regulators of *NOS2* expression, they may represent points of convergence between T-cell differentiation and NO-dependent immune responses. However, direct evidence for NOS2 regulation by KLF proteins in intestinal T cells remains lacking.

Collectively, current evidence suggests that NOS2 is not a major determinant of global T-cell activation but rather a context-dependent regulator of specialized lymphocyte functions. The strongest data support roles in IFNγ-responsive pathways, γδ T-cell biology, and HIF1α-dependent Th9 differentiation, whereas evidence linking other signaling and transcriptional regulators to NOS2 expression in intestinal T cells remains largely indirect. Most mechanistic insights derive from murine and in vitro studies, and the contribution of NOS2-expressing T-cell subsets to human IBD remains poorly defined. Consequently, T cells are likely to influence intestinal NO homeostasis predominantly through cytokine-mediated regulation of NOS2 expression in macrophages, epithelial cells, and other cell populations rather than through direct NO production.

### 8.4. NKcells

Compared with macrophages, dendritic cells, or T cells, the regulation of NOS2 expression in NK cells remains relatively poorly characterized. Existing evidence, summarized by Cifone and colleagues [483], indicates that *NOS2* can be induced in activated NK cells, although the reported mechanisms and functional consequences vary considerably between experimental systems. In rat NK cells, IL-2 induces NOS2 expression at both mRNA and protein levels, and NOS2-derived NO contributes to cytotoxic activity and IFNγ production [670]. Consistent with these findings, studies in iNOS-deficient mice demonstrated impaired NK-cell responses during infection, supporting a functional role for NOS2 in innate immunity in vivo [671]. Human studies have yielded less consistent results. While IL-12 and TNFα induce NOS2 expression in human NK cells and generate NO that negatively regulates lytic activity [672], other investigators failed to detect substantial *NOS2* induction and instead identified constitutive NOS3 expression as the principal source of NO [673]. These findings suggest that NO may function as either an effector molecule or an autoregulatory signal depending on the activation context and species studied.

However, the relevance of these observations to intestinal immunity remains uncertain. Although NK cells are increasingly recognized as important regulators of mucosal homeostasis and inflammation, direct evidence linking NOS2 expression in NK cells to intestinal disease is lacking. Current knowledge of gut-associated NK cells derives primarily from studies examining their cytokine production, interactions with epithelial cells, and responses to IL-12 and IL-23 rather than from investigations of NO signaling [674].

Unlike macrophages, dendritic cells, and even selected T-cell subsets, NK cells have not been extensively investigated as a source of NOS2 in the intestine. Consequently, whether NO contributes to the specialized regulatory functions of gut-associated NK cells in health and IBD remains an important unresolved question.

### 8.5. Myeloid-Derived Suppressor Cells (MDSCs)

Myeloid-derived suppressor cells (MDSCs) comprise a heterogeneous population of pathologically activated immature myeloid cells with potent immunomodulatory activity. They suppress T-, B-, and NK-cell responses through several mechanisms, including production of reactive oxygen and nitrogen species (RONS) and depletion of L-arginine by arginases and NOS2 [480]. However, the relative contribution of these pathways differs between MDSC subsets. Granulocytic MDSCs primarily employ peroxynitrite-mediated mechanisms, whereas monocytic MDSCs are characterized by high NOS2 expression and preferentially utilize NO-mediated immunosuppression [480].

L-arginine depletion and RONS production impair lymphocyte function by reducing proliferation, limiting migration, and promoting apoptosis [480]. Nevertheless, the role of MDSCs in intestinal inflammation appears more complex than their classical immunosuppressive designation suggests. Studies in experimental colitis models have reported that intestinal MDSCs can promote rather than suppress inflammation by enhancing T-cell proliferation and stimulating IL-17 production, thereby contributing to the persistence of mucosal inflammation [675,676]. Similarly, peripheral MDSCs isolated during intestinal inflammation have been reported to acquire a pro-inflammatory phenotype capable of promoting T-cell expansion [676].

In contrast, studies in patients with active CD and UC have demonstrated increased frequencies of circulating MDSCs exhibiting immunosuppressive properties [677]. These apparently contradictory findings likely reflect the marked heterogeneity and phenotypic plasticity of MDSCs, which are strongly influenced by the local cytokine milieu, tissue microenvironment, and disease activity. Recent analyses have reinforced the view that MDSCs represent a highly diverse population whose abundance, phenotype, and function vary substantially among patients and phases of disease, thereby explaining reports describing both protective and pathogenic roles in IBD [678].

Among the pathways regulating *NOS2* expression in MDSCs, IFNγ/STAT1 signaling appears to be particularly important. STAT1 activation promotes *NOS2* transcription and is required for the NO-dependent suppressive activity of monocytic MDSCs, linking inflammatory cytokine signaling directly to MDSCs-mediated immune regulation [678]. Beyond IFNγ/STAT1 signaling, pathways involving STAT3, NF-κB, and the alarmins S100A8 and S100A9 have been implicated in the expansion and activation of NOS2-expressing MDSCs in other inflammatory settings, although their specific contribution to intestinal inflammation remains less well defined [679,680]. Additional regulation is provided through C/EBPβ-dependent pathways, which are important for MDSCs differentiation and suppressive activity and can influence *NOS2* expression either directly or through associated lncRNA networks. As discussed in Chapter 5, lncRNAs including lnc-CHOP, lnc-C/EBPβ, RNCR3, and Olfr29-ps1 modulate *NOS2* expression and the immunoregulatory functions of MDSCs [394,395,396,397,398,399].

Collectively, MDSCs represent one of the major NOS2-expressing immune-cell populations outside the macrophage lineage. Current evidence suggests that NO is a principal mediator of monocytic MDSC function, yet the consequences of MDSC accumulation in IBD remain context-dependent. Human studies generally support an immunosuppressive role for MDSCs, whereas experimental models have also identified pro-inflammatory activities that may perpetuate intestinal inflammation. This apparent paradox likely reflects the remarkable heterogeneity and plasticity of MDSCs and highlights the need to better define the specific NOS2-expressing MDSC subsets involved in intestinal homeostasis and IBD.

### 8.6. Neutrophils

Neutrophils are among the first immune cells recruited to sites of intestinal injury and infection and constitute an important source of reactive oxygen and nitrogen species. Unlike mast cells [681], both murine [456] and human neutrophils [682] constitutively express NOS2. In murine neutrophils, NOS2 has been detected in the nucleus, cytoplasm, azurophilic granules, mitochondria, and phagocytic cup, whereas in human neutrophils it appears to be localized predominantly to membrane-associated compartments [456,683]. Activated neutrophils additionally express NOS1, while the presence and functional significance of NOS3 remain controversial [479]. Unlike macrophages, which generally require activation for robust *NOS2* induction, neutrophils constitutively express NOS2 and regulate NO production primarily through changes in enzyme abundance, subcellular localization, and activation state [456,682].

Neutrophil-derived NO contributes directly to antimicrobial defense through generation of reactive nitrogen species and cooperation with oxidative killing mechanisms. In addition, NO exerts autocrine and paracrine regulatory effects on neutrophil function, including modulation of migration, activation, and release of neutrophil extracellular traps (NETs) [479]. Given the emerging role of NETs in epithelial injury and amplification of intestinal inflammation, NOS2-dependent regulation of neutrophil effector functions may have broader consequences for mucosal homeostasis. Efficient microbial killing requires Rac2-dependent trafficking of NOS2 to phagosomes, enabling coordinated RONS generation [684].

Baseline NOS2 expression is detectable in neutrophils from healthy individuals and is further increased by microbial products and inflammatory cytokines through NF-κB-dependent signaling [684,685]. Upstream regulation of NF-κB signaling involves TAK1, which is constitutively associated with the IKK complex and contributes to rapid inflammatory activation in neutrophils [686]. Although a direct role of TAK1 in neutrophil *NOS2* regulation has not been established, its central position within NF-κB signaling suggests a potential contribution to *NOS2* induction. Interestingly, excessive oxidative stress generated by NOX2 can suppress *NOS2* transcription by inducing S-glutathionylation of the NF-κB subunits p65 and p50, thereby limiting their binding to the *NOS2* promoter [685]. These observations indicate that NOS2 expression in neutrophils is controlled by both pro-inflammatory activation pathways and redox-dependent negative-feedback mechanisms.

The contribution of neutrophils to IBD appears to differ between disease phenotypes. Recent single-cell and transcriptomic studies have revealed considerable neutrophil heterogeneity in IBD and challenged the traditional view of neutrophils as exclusively tissue-damaging cells. Distinct neutrophil populations may either promote inflammation through production of ROS, proteases, and extracellular traps or facilitate resolution of inflammation and mucosal repair [687]. In UC, neutrophil accumulation is a defining histopathological feature and correlates strongly with disease activity, whereas impaired neutrophil recruitment and defective acute inflammatory responses have been proposed to contribute to defective bacterial clearance and persistent inflammation in CD [688]. Evidence from IL-10R^−^/^−^ mice further suggests that neutrophils may represent a major source of pathogenic NOS2-derived NO, as selective neutrophil depletion markedly reduces intestinal NO production and attenuates colitis severity [341].

Collectively, neutrophils represent an important source of NOS2-derived NO in the intestinal mucosa. Unlike macrophages, they constitutively express NOS2 and regulate NO production through activation-dependent and compartment-specific mechanisms. Current evidence suggests that neutrophil-derived NO contributes both to antimicrobial defense and to inflammatory tissue injury, reflecting the dual role of neutrophils in intestinal homeostasis and disease. Emerging single-cell studies further indicate that distinct neutrophil subsets may differentially contribute to NOS2-dependent functions in IBD, highlighting neutrophil heterogeneity as an important area for future investigation.

### 8.7. Mast Cells

Mast cells are important regulators of epithelial barrier integrity, vascular permeability, mucosal immunity, and neuroimmune communication [481]. Beyond their established role in allergic disorders, mast cells are increasingly recognized as active participants in IBD pathogenesis through interactions with epithelial, immune, and neuronal cells. Recent single-cell transcriptomic studies have revealed considerable heterogeneity among intestinal mast cells, identifying tissue-specific subsets with distinct transcriptional and functional profiles [689]. These findings challenge the traditional view of mast cells as a homogeneous population and suggest that their contribution to intestinal inflammation is highly context dependent.

Upon activation, mast cells express both NOS1 and NOS2 [479,481] and are capable of both producing and responding to NO. Human mast cells express functional NOS2, and its expression can be induced by inflammatory stimuli, although the regulatory mechanisms appear less well characterized than those described for macrophages and other myeloid cells [681]. Early studies demonstrated that NOS-derived NO suppresses mast-cell degranulation and limits histamine-induced increases in epithelial permeability [690]. These observations initially suggested that NO contributes to maintenance of mucosal barrier integrity. However, subsequent investigations indicated that these protective effects are mediated predominantly by constitutively expressed NOS isoenzymes rather than inducible NOS2 [691]. Thus, unlike macrophages or neutrophils, mast-cell biology appears to be influenced more strongly by NO signaling itself than by robust NOS2 induction.

Recent evidence has further implicated mast cells in impaired mucosal healing in IBD. Analyses of human intestinal tissue and experimental colitis models indicate that mast-cell activation persists during active inflammation and may contribute not only to immune activation but also to defective epithelial restitution [481,692]. Beyond their effects on vascular permeability and leukocyte recruitment, mast cells release proteases, cytokines, growth factors, and lipid mediators capable of influencing epithelial turnover, barrier function, and tissue remodeling. These observations suggest that mast cells may contribute to both inflammation and abnormal repair processes within the intestinal mucosa.

Collectively, mast cells are increasingly recognized as important regulators of intestinal inflammation, barrier function, neuroimmune signaling, and mucosal repair. Although mast cells express NOS2 and can both produce and respond to NO, the best-characterized NO-dependent effects in these cells appear not to be mediated by NOS2. Consequently, in contrast to macrophages, neutrophils, or MDSCs, the specific contribution of mast cell-derived NOS2 to intestinal homeostasis and IBD remains poorly defined. Future studies addressing NOS2 regulation within distinct mast-cell subsets may help clarify whether NO contributes to the functional heterogeneity recently revealed by single-cell analyses.

## 9. Conclusions and Future Directions

NOS2 is one of the most extensively studied enzymes associated with intestinal inflammation and a major source of nitric oxide in IBD. Current evidence indicates that NOS2-derived NO exerts both protective and pathogenic effects, participating in antimicrobial defense, epithelial restitution, vascular regulation, immune homeostasis, fibrosis, and tissue injury. Rather than being controlled by a single regulatory pathway, NOS2 expression emerges as the product of a complex, multilayered network operating at transcriptional, post-transcriptional, translational, and post-translational levels.

A central theme arising from recent studies is that regulation of NOS2 extends substantially beyond the classical IFNγ/STAT1 and NF-κB pathways. Epigenetic mechanisms, microRNAs, long non-coding RNAs, mRNA stability, protein turnover, and intracellular trafficking all contribute to shaping the magnitude, duration, and biological consequences of NO production. In parallel, tissue-specific factors including microbial metabolites, hypoxia, iron availability, ER stress responses, neuroimmune signaling, and immunometabolic reprogramming have emerged as important determinants of NOS2 activity within the intestinal mucosa.

Another major advance has been the recognition of marked cellular heterogeneity in NOS2 expression. Single-cell and spatial transcriptomic studies have revealed that NOS2 is distributed among diverse epithelial, stromal, endothelial, and immune-cell populations with distinct transcriptional programs and functional properties. Importantly, NOS2 does not serve equivalent functions across cell types. While macrophages, neutrophils, and monocytic MDSCs represent major effector populations, dendritic cells, selected lymphocyte subsets, and possibly NK cells appear to employ NO in more specialized immunoregulatory contexts. These observations challenge traditional interpretations based on bulk-tissue analyses and emphasize the importance of studying NOS2 within its cellular and spatial context.

Despite considerable progress, important knowledge gaps remain. Much of the current understanding of NOS2 regulation derives from cell-culture systems and experimental models, whereas direct evidence from human IBD tissues is still limited for many regulatory pathways and cell populations. Future studies should therefore integrate mechanistic investigations with single-cell, spatial, and longitudinal human datasets to define how specific NOS2-expressing populations contribute to disease initiation, progression, fibrosis, mucosal healing, and therapeutic response.

Ultimately, successful translation of NOS2 biology into clinical practice will likely require a shift from global manipulation of NO production toward cell-specific and context-dependent therapeutic strategies. A deeper understanding of the molecular, cellular, and tissue-level regulation of NOS2 may facilitate biomarker development, patient stratification, and identification of novel therapeutic targets. Rather than serving solely as an inflammatory effector, NOS2 increasingly emerges as a sensitive indicator of the immune-metabolic state of intestinal tissues and a central integrator of microbial, immune, and environmental signals that shape the course of IBD.

## Figures and Tables

**Table 1:** Transcriptional regulatory network controlling human NOS2 expression in the intestine.

Region/ Regulatory Factor/Pathway ^1^	NOS2 Regulatory Element(s) ^1^	Major Activating Stimulus(es)	Effect on *NOS2* Expression	Functional Significance/Role in IBD
Core promoter (<3.8 kb)	Multiple factors	Basal transcription machinery	Basal transcription	Supports constitutive *NOS2* expression under homeostatic conditions, particularly in IECs Inducible by IL-1β in IECs and ECs
NF-κB (RelA/p50)	κB elements (multiple sites)	TLR ligands, NOD2 agonists, TNFα, IL-1β	Master inducer	Integrates microbial and cytokine signaling Sustains chronic mucosal inflammation Integrates signaling from IBD risk loci Indirect target of multiple IBD therapies
STAT1/GAF	GAS elements (multiple sites)	IFNγ	Essential inducer	Activated in inflamed IBD mucosa Broadly activated across intestinal cell populations Sustains IFNγ-driven *NOS2* expression Enhanced by defective PTPN2/SOCS1 regulation
IRF1 and related IRFs	IRF-E, ISRE (multiple sites)	IFNγ, type I IFNs	IRF1: essential cofactor IRF8: required cofactor	Essential cofactors for IFNγ-induced *NOS2* Cooperate with NF-κB in chronic inflammation Linked to IBD susceptibility loci Contribute to inflammation-associated tumorigenesis
AP-1	AP-1-binding sites	Cytokines, MAPK signaling, oxidative stress	Major activator	Sustains inflammation-driven *NOS2* expression Integrates microbial, cytokine, and stress signals AP-1 blockade ameliorates experimental colitis
HIF1α/HIF2α	HRE (distal promoter)	Hypoxia	Major activators	Upregulated in inflamed IBD tissue Drives hypoxia-dependent *NOS2* expression Cooperates with NF-κB and STAT1 pathways Contributes to chronic inflammation and tissue damage
KLF4	K-BE (−0.095 and −0.21 kb)	IFNγ, LPS, inflammatory signaling	Direct activator	Promotes inducible *NOS2* expression Supports epithelial homeostasis Exhibits context-dependent inflammatory effects Inconsistent changes in IBD
KLF6	K-BE (−0.164 and −0.261 kb)	TLR signaling, TNFα, oxidative stress, hypoxia	Direct activator	Upregulated in inflamed IBD mucosa Drives *NOS2* expression and M1 responses Its deficiency attenuates experimental colitis
FOXO3	F-BS (~−1.53 kb)/indirect mechanisms	Reduced PI3K/Akt signaling	Direct repressor	Endogenous brake on intestinal inflammation Reduced expression/activity in IBD Its deficiency increases susceptibility to colitis/CAC
Retinoic acid signaling (RAR/RXR, PPAR/RXR)	RARE; PPRE; indirect mechanisms	Retinoic acid	Bimodal regulator	Context-dependent effects on *NOS2* expression and inflammation Promotes immune tolerance under homeostatic conditions May enhance inflammatory responses in active IBD Elevated in inflamed IBD mucosa
ELF3 and ETS2	ETS-binding elements	Inflammatory stimuli (LPS/TLR-NF-κB)	Secondary activators	ELF3: NF-κB-cooperative amplifier of inflammatory responses ETS2: Driver of IBD-like inflammatory macrophage programs
ELK3 and ELF4		TGFβ/homeostatic signals	Secondary repressors	ELK3: Mediates TGFβ-dependent anti-inflammatory signaling ELF4: Maintains immune homeostasis ELF4: Deficiency associated with IBD-like disease
HMG proteins	AT-RE (~−3.5 to −3.8 kb)	Inflammatory cytokines/NF-κB	Architectural cofactors	Amplify NF-κB-driven inflammation Facilitate inflammatory transcriptional complexes HMGB1 linked to IBD gene-expression signatures
Oct-1	OREs (~−10.2 kb; ~−0.06 kb)	Inflammatory cytokines/NF-κB	Facilitating cofactor	Supports cytokine-induced inflammatory responses Promotes mucosal repair Linked to IBD-associated TNF regulation
TCF4/β-catenin	TBEs (−3.83 kb; −6.13 kb)	Wnt/β-catenin signaling	Secondary activator	Reduced activity in ileal CD Maintains Paneth-cell antimicrobial defense Supports epithelial barrier/homeostasis
C/EBPβ (NF-IL6)	AABS/NF-IL6 site (multiple)	Inflammatory cytokines/NF-κB	Secondary activator	Associated with IBD inflammatory transcriptional programs Promotes mucosal inflammation Contributes to experimental colitis
XBP1	AABS	ER stress, IRE1 activation	Stress-responsive activator	Links UPR to *NOS2* transcription IBD susceptibility gene Maintains epithelial and Paneth-cell homeostasis Deficiency causes spontaneous enteritis and colitis susceptibility
CNC-bZIP (TCF11/NRF1; NRF2)	NF-E2; distal AREs	TGFβ/SMAD6; redox stress	Stress-responsive repressors	Maintains redox homeostasis Restrains inflammatory signaling Limits excessive NO production
TGFβ-SMAD2/3/4	Indirect regulation	TGFβ	Major indirect repressor	Maintains intestinal immune tolerance Impaired by SMAD7 overexpression in IBD Loss promotes chronic intestinal inflammation Therapeutic restoration explored via SMAD7 inhibition
Distal epithelial enhancer region	−10.7 to −8.7 kb	Multiple inflammatory stimuli	Essential for maximal induction	Critical for robust *NOS2* expression in IECs
Distal NF-κB/STAT1-responsive enhancer	~−6.2 to −5.0 kb	TNFα, IL-1β, IFNγ	Strong activation	Major enhancer driving inducible human *NOS2* transcription

^1^ Locations are approximate and based on the regions discussed in cited studies. AABS, ‘A activator-binding site’; AP-1, activator protein 1; ARE, antioxidant response element; AT-RE, AT-rich enhancer elements; CAC, colitis-associated cancer; CD, Crohn’s disease; C/EBPβ, ‘CCAAT/enhancer-binding protein β’; CNC-bZIP, ‘Cap’n’Collar basic leucine zipper’; ECs, endothelial cells; ELF, ‘E74-like ETS transcription factor’; ELK, ‘ETS-like transcription factor’; ER, endoplasmic reticulum; F-BS, FOXO3A-binding sequence; FOXO3, ‘Forkhead box O3’; GAF, ‘gamma-interferon activation factor’; GAS, gamma-activated sequence; HIF,’ hypoxia-inducible factor’; HRE, ‘hypoxia-response element’; IBD, inflammatory bowel disease; IECs, intestinal epithelial cells; IFNγ, interferon-γ; IL, interleukin; IRF, interferon regulatory factor; IRF-E, ‘interferon regulatory factor element’; ISRE, interferon-stimulated response element; JAK, Janus kinase; kb, kilo bases; K-BE, KLF-binding element; KLF, Krüppel-like factor; LPS, lipopolysaccharide; MAPK, ‘mitogen-activated kinase’; NF-κB, ‘nuclear factor κ-light-chain-enhancer of activated B-cells’; NF-E2, ‘nuclear factor erythroid 2-related factor binding element’; NOS2, inducible nitric oxide synthase; NOD2, ‘nucleotide-binding oligomerization domain-containing protein 2’; NRF1, ‘nuclear factor erythroid 2-related factor 1’ (TCF11/NFE2L1); NRF2, ‘nuclear factor erythroid 2-related factor 2’ (NFE2L2); Oct, ‘octamer-binding transcription factor’; ORE, ‘octamer-response element’; PI3K/Akt, ‘phosphoinositide 3-kinase/protein kinase B’; PPAR, ‘peroxisome proliferator-activated receptor’; PPRE, ‘peroxisome proliferator response element’; PTPN2, ‘protein tyrosine phosphatase non-receptor type 2’; RAR, ‘retinoic acid receptor’; RXR, ‘retinoid X receptor’; SMAD, ‘suppressor of mothers against decapentaplegic homolog’; SOCS1, ‘Suppressor of Cytokine Signaling 1’; STAT1, ‘signal transducer and activator of transcription 1’; TBE, TCF4-binding element; TCF4, T-cell factor 4; TGFβ, transforming growth factor-β; TLR, ‘Toll-like receptor’; TNFα, tumor necrosis factor α; UPR, unfolded protein response; XBP1, ‘X-box-binding protein 1’.

**Table 2:** NF-κB-activating pathways in IBD and their relevance for *NOS2* induction.

NF-κB-Activating Pathway	Main Trigger	Principal Mucosal Cells	IBD Phenotype	Contribution to *NOS2* Induction	Mode of *NOS2* Induction
TLR4 → MyD88 → IRAK → TRAF6 → IKK	LPS, DAMPs	Mφ, DCs, IECs	CD and UC	Very high	Major direct inducer; strong IFNγ synergy
TLR2 → MyD88 → TRAF6 → IKK	Bacterial lipoproteins, PGN, LTA, zymosan	Mφ, DCs, IECs	CD and UC	High	Direct microbial inducer
TLR5 → MyD88 → TRAF6 → IKK	Flagellin	IECs, Mφ, DCs	Mainly CD, also UC	Moderate-high	Direct flagellin-responsive inducer
TLR9 → MyD88 → TRAF6 → IKK	Bacterial and fungal CpG DNA	DCs, Mφ, IECs	CD and UC	Moderate	Conditional microbial inducer
TLR3 → TRIF → RIPK1 → TAK1	dsRNA (viral or DAMPs)	Mφ, DCs, IECs	CD and UC	Moderate	IFN/IRF-dependent inducer
NOD2 → RIPK2 → TAK1 → IKK	MDP	Mφ, DCs, Paneth cells	Mainly CD	High	Direct inducer; potentiates TLR signaling
TNFα → TNFR1/TNFR2 → RIPK1/TRAF2 → IKK	TNFα	Mφ, IECs, fibroblasts, ECs	CD and UC	Moderate-high	Inflammatory amplifier and maintainer
IL-1β → IL-1R → MyD88 → IRAK → TRAF6 → IKK	IL-1β	Mφ, IECs, fibroblasts, ECs	CD and UC	High	Strong direct inflammatory inducer
IL-17A/F → IL-17R → ACT1 → TRAF6	IL-17A, IL-17F	IECs, fibroblasts	Mainly CD, also active in UC	Low-moderate	Cytokine-dependent amplifier
IL-23/Th17 axis	IL-23	Th17 cells, ILC3	Mainly CD	Indirect	Indirect enhancer via IL-17
BCR/CD40/BAFFR	Antigen, CD40L, BAFF	B cells, PCs	Both; relatively more evident in UC	Minimal-direct	Minimal direct contribution
LTβR/CD40/BAFFR → NIK → p52/RelB (non-canonical NF-κB)	BAFF, CD40L, lymphotoxin	B cells, stromal cells, DCs	Chronic CD and UC	Low	Indirect contributor to chronic inflammation

ACT1, ‘activator of NF-κB 1’; BAFF, ‘B-cell activating factor’; BAFFR, BAFF receptor; BCR, B-cell receptor; CD, Crohn’s disease; DCs, dendritic cells; DAMPs, ‘damage-associated molecular patterns’; dsRNA, double-stranded RNA; ECs, endothelial cells; IBD, inflammatory bowel disease; IECs, intestinal epithelial cells; IFN, interferon; IL, interleukin; IKK, IκB kinase; ILC3,’ group 3 innate lymphoid cells’; IRAK, ‘IL-1 receptor-associated kinase’; IRF, interferon regulatory factor; LPS, lipopolysaccharide; LTA, lipoteichoic acid; LTβR, lymphotoxin-β receptor; Mφ, macrophages; MDP, muramyl dipeptide; MyD88, ‘myeloid differentiation primary response protein 88’; NF-κB, ‘nuclear factor κ-light-chain-enhancer of activated B-cells’; NIK, ‘NF-κB-inducing kinase’; NOD2, ‘nucleotide-binding oligomerization domain-containing protein 2’; NOS2, inducible nitric oxide synthase; PGN, peptidoglycan; RIPK, ‘receptor-interacting protein kinase’; STAT1, ‘signal transducer and activator of transcription 1’; TAK1, ‘transforming growth factor-β-activated kinase 1’; Th17, T helper 17 cell; TLR, ‘Toll-like receptor’; TNF, ‘tumor necrosis factor’; TNFR, ‘tumor necrosis factor receptor’; TRAF, ‘TNF receptor-associated factor’; TRIF, ‘TIR-domain-containing adapter-inducing interferon-β’; UC, ulcerative colitis.

**Table 3:** MicroRNAs directly and indirectly implicated in *NOS2* regulation and their relevance to IBD.

miR	Expression in IBD	Direct *NOS2* Target	Major Target(s) /Mechanism	Effect on *NOS2*	Potential Significance in IBD
miR-26a-5p	↑ UC/↑ CD	Yes	Direct binding to *NOS2* mRNA; *HMGA1*	↓	Anti-inflammatory regulator Limits NF-κB-dependent *NOS2* expression
miR-939-5p	↓ UC lesions	Yes	Direct binding to *NOS2* 3′UTR	↓	Reduced expression may: favor *NOS2* upregulation during inflammation predict poor outcome in CRC
miR-146a	↑ UC/↑ CD	In mice	(*NOS2*), *IRAK1*, *TRAF6*, *RIPK2*, *STAT1*	↓	Negative-feedback regulator of NF-κB and IFNγ signaling Fosters immune suppression ↑ Linked to mucosal barrier dysfunction and impaired immunity Role in experimental colitis is inconsistent Antitumor in experimental CRC
miR-155	↑ UC/↑ CD	No	*SOCS1*, *SHIP1*, *SMAD2*, *FOXO3A*; *BCL6*, *MYD88*, *CEBPB*, *TAB2*	Mostly ↑	Promotes M1 and Th1 and differentiation of Treg and Th17 Deficiency limits intestinal inflammation in experimental colitis Can act pro- and anti-inflammatory
miR-29a/b	↑ UC/↑ CD	No	IL-12/23 pathway, *ATF2*, *HMGB1*	↓	May restrain IL-23-driven inflammation and *NOS2* induction
miR-21	↑ UC/↑ CD	No	*TLR4*, *IRAK2/4*, *PDCD4*, *PTEN*	Context-dependent	Regulates inflammatory resolution and macrophage activation
miR-126	↑ UC/↑ CD	No	*IKBA*, *HMGB1*	Context-dependent	Modulates endothelial inflammation and NF-κB signaling
miR-98-5p	↑ UC	No	*TRIB1*	↑	Promotes M1 polarization and *NOS2* expression
miR-9	↑ CD lesions	No	*RUNX1*	↑	May favor inflammatory responses through *RUNX1* suppression
miR-369-3p	↓ IBD	In mice	(*NOS2*), *TNFA*, *CEBPB*, *NFKB*	↓	Anti-inflammatory regulator; suppresses DCs activation
miR-200 family	Variable	No	*KLF6*, *HMGB1*, *HIF1A*, *ROCK2*	↓	May limit inflammatory and EMT-associated pathways
miR-31	↑ UC/↑ CD	No	*RHOA*	↓	Protective in experimental colitis
miR-16	↑ UC/ ↑ CD blood	No	A2AR pathway	↑	May enhance NF-κB-dependent inflammation
miR-124	↓ pediatric UC	No	*STAT3*	↓	Promotes M2 and suppresses inflammatory signaling
miR-4262	↑ pediatric IBD	No	*SIRT1*	↑	Enhances NF-κB/AP-1 activity and favors *NOS2* expression
miR-185-5p	↓ IBD/↓ CRC	No	*CHOP*	↓	May indirectly restrain C/EBPβ-dependent *NOS2* expression
miR-214-3p	↓ UC/↓ CRC	No	*MYD88*, *STAT6*	↓	Limits NF-κB signaling

A2AR, ‘adenosine A2A receptor’; AP-1, activator protein 1; ATF2, ‘activating transcription factor’ 2; BCL6, ‘B-cell lymphoma 6 protein’; CD, Crohn’s disease; CEBPB and C/EBPβ, ‘CCAAT/enhancer-binding protein’ β; CHOP, ‘CCAAT/enhancer-binding protein (C/EBP) homologous protein’; CRC, colorectal cancer; DCs, dendritic cells; EMT, epithelial–mesenchymal transition; FOXO3A, ‘forkhead box O3a’; HIF1A, hypoxia-inducible factor 1α; HMG, ‘high mobility group protein’; IBD, inflammatory bowel disease; IFNγ, interferon-γ; IKBA, inhibitory κBα; IL, interleukin; IRAK, ‘interleukin-1 receptor-associated kinase’; KLF6, Krüppel-like factor 6; M1/M2, classically/alternatively activated macrophages; miR, microRNA; MYD88, ‘myeloid differentiation primary response protein 88’; NF-κB, ‘nuclear factor κ-light-chain-enhancer of activated B-cells’; NOS2, inducible nitric oxide synthase; PDCD4, ‘programmed cell death protein’ 4; PTEN, ‘phosphatase and tensin homolog’; RHOA, ‘Ras homolog family member A’ GTPase; RIPK, ‘receptor-interacting protein kinase’; ROCK, ‘Rho-associated coiled-coil-containing protein kinase’; RUNX1, ‘runt-related transcription factor’ 1; SHIP1, ‘SH2 domain-containing inositol 5-phosphatase’ 1; SIRT1, sirtuin 1; SMAD2, ‘suppressor of mothers against decapentaplegic’ 2; SOCS1, ‘suppressor of cytokine signaling’ 1; STAT1/3/6, ‘signal transducer and activator of transcription’ 1/3/6; TAB2, ‘TGFβ-activated kinase 1-binding protein 2’; TLR, Toll-like receptor; TNFA, tumor necrosis factor α; TRAF6, ‘TNF receptor-associated factor’ 6; TRIB1, tribbles homolog 1; UC, ulcerative colitis; UTR, untranslated region.

**Table 4:** Long non-coding RNAs and pseudogene-derived transcripts implicated in *NOS2* regulation.

lncRNA	Mechanism	Effect on *NOS2*	Biological Significance
HCG18	Sponges miR-146a	↑	Promotes M1 polarization
CHRF	Sponges miR-146a	↑	Promotes M1 polarization
HEIH	Sponges miR-939-5p	↑	Associated with tumor progression
MEG3	Sponges miR-98-5p	↓	Protects against experimental colitis
lnc-CHOP	Releases active C/EBPβ LAP; promotes H3K4 methylation	↑	Enhances MDSCs’ suppressive activity
lnc-C/EBPβ	Stabilizes inhibitory LIP-LAP complex	↓	Suppresses *NOS2* transcription
RNCR3	Sponges miR-185-5p	↑	Promotes CHOP-dependent *NOS2* expression
Olfr29-ps1	Sponges miR-214-3p	↑	Enhances MyD88 signaling and *NOS2* expression
NOS2P3	Sponges miR-939-5p	↑	Increases *NOS2* and *TNFA* expression

C/EBPβ, ‘CCAAT/enhancer-binding protein β’; CHOP, ‘C/EBP homologous protein’ (GADD153); CHRF, ‘cardiac hypertrophy-related factor’; HCG18, ‘HLA complex group 18’; HEIH, ‘high expression in hepatocellular carcinoma’; H3K4, histone H3 lysine 4; LAP, ‘liver-enriched activator protein isoform’ of C/EBPβ; LIP, ‘liver-enriched inhibitory protein isoform’ of C/EBPβ; lncRNA, long non-coding RNA; M1, inflammatory macrophages; MDSCs, myeloid-derived suppressor cells; MEG3, ‘maternally expressed gene’ 3; miRNA (miR), microRNA; MyD88, ‘myeloid differentiation primary response protein 88’; NOS2, inducible nitric oxide synthase; NOS2P3, ‘nitric oxide synthase 2 pseudogene 3’; Olfr29-ps1, ‘Olfactory receptor 29, pseudogene 1’; RNCR3, retinal non-coding RNA 3; TNFA, tumor necrosis factor α.

**Table 5:** NOS2 expression in non-immune intestinal cells: functions, regulation, and relevance to IBD.

Cell Type	NOS2 Expression	Principal Inducers/ Regulators	Proposed Functions of NOS2	Overall Relevance to IBD
IECs	Constitutive, strongly inducible	Microbial products, cytokines	Barrier regulation, antimicrobial defense, carcinogenesis	High
LNDs	High	Inflammation-associated reprogramming	Antimicrobial and immunoregulatory	High
IMFs	Inducible	IFNγ, TNFα, IL-22, hypoxia	Repair, fibrosis, immune modulation	Moderate
SMCs	Inducible	IL-1β, TNFα, LPS, mechanical stress	Dysmotility, smooth-muscle dysfunction	Moderate
ECs	Weakly inducible	Hypoxia, shear stress, TLR/NOD signaling	Leukocyte adhesion, angiogenesis, vascular homeostasis	High
EGCs	Induced in inflammation	Colitis-associated inflammatory signaling	Barrier dysfunction, ion transport abnormalities	Low- Moderate
ENs	Predominantly NOS1	Inflammatory stimuli	Neuroimmune regulation, motility	Low

CD, Crohn’s disease; DUOX2, dual oxidase 2; ECs, endothelial cells; EGCs, enteric glial cells; ENs, enteric neurons; IBD, inflammatory bowel disease; IECs, intestinal epithelial cells; IFNγ, interferon-γ; IL, interleukin; IMFs, intestinal myofibroblasts; LCN2, lipocalin-2; LNDs, LCN2-NOS2-DUOX2-positive epithelial cells; LPS, lipopolysaccharide; NOS2, inducible nitric oxide synthase; NOD, nucleotide-binding oligomerization domain-containing protein; SMCs, smooth muscle cells; TLR, toll-like receptor; TNFα, tumor necrosis factor α; UC, ulcerative colitis.

**Table 6:** NOS2 expression in immune intestinal cells: functions, regulation, and relevance to IBD.

Cell Type	NOS2 Expression	Principal Inducers/ Regulators	Proposed Functions of NOS2	Overall Relevance to IBD
Mφ	Strongly upregulated; enriched in inflammatory macrophage subsets	TLR/NF-κB, IFNγ/JAK/STAT1, HIF1α, MAPK, P2X7R; inhibited by IL-10, TGFβ, PI3K/Akt1, PPARs	Antimicrobial defense, M1 polarization, cytokine production, regulation of adaptive immunity, fibrosis and carcinogenesis	High
DCs	Inducible	TLRs, NOD2, CLRs, IFNγ; inhibited by NRF2-dependent pathways	Regulation of DC differentiation, T-cell activation, antibacterial responses, maintenance of tolerance	Moderate
T cells	Inducible in activated T-cell subsets, particularly γδ IELs	IFNγ/STAT1, NF-κB, TAK1; inhibited by TGFβ	Modulation of T-cell activation and mucosal immunity; contribution to epithelial repair through γδ T-cell responses	Low-Moderate
NK cells	Express NOS2 following activation	IL-12, inflammatory stimuli	Antimicrobial defense, IFNγ production, regulation of epithelial survival through IL-22-producing subsets	Low
MDSCs	Highly expressed in monocytic MDSCs	IFNγ/STAT1, inflammatory mediators	Immunoregulation through NO production, T-cell suppression, modulation of chronic inflammation	Moderate
NEUs	Constitutively expressed and further induced during inflammation	NF-κB, cytokines, microbial products	Microbial killing, RONS generation, regulation of neutrophil activation and NET formation; potential tissue injury	High
MCs	Inducible following activation	Poorly characterized	Regulation of barrier integrity, neuroimmune signaling, vascular permeability; role of NOS2 remains unclear	Low

CD, Crohn’s disease; CLR, C-type lectin receptor; DCs, dendritic cells; HIF1α, hypoxia-inducible factor 1α; IBD, inflammatory bowel disease; IEL, intraepithelial lymphocyte; IFNγ, interferon-γ; IL, interleukin; JAK, Janus kinase; Mφ, macrophages; MAPK, mitogen-activated protein kinase; MCs, mast cells; MDSCs, myeloid-derived suppressor cells; NEUs, neutrophils; NET, neutrophil extracellular trap; NF-κB, nuclear factor κB; NK, natural killer; NO, nitric oxide; NOD, nucleotide-binding oligomerization domain-containing protein; NOS2, nitric oxide synthase 2; NRF2, ‘Nuclear factor erythroid 2-related factor 2’; PI3K/Akt1; posphoinositide 3-kinase/AKT serine/threonine kinase 1; PPAR, peroxisome proliferator-activated receptor; RONS, reactive oxygen and nitrogen species; STAT, signal transducer and activator of transcription; TAK1, TGFβ-activated kinase 1, TGFβ, transforming growth factor β; TLR, toll-like receptor.

## Data Availability

No new data were created or analyzed in this study. Data sharing is not applicable to this article.

## List of Abbreviations

43S PIC	43S preinitiation complex
4ESE	eIF4E sensitivity element
5-ASA	5-aminosalicylic acid
αMSH	α-melanocyte-stimulating hormone
A2AR	Adenosine A2A receptor
AABS	A activator-binding site
ADMA	Asymmetric dimethylarginine
Akt	Protein kinase B; PKB
ALK	Anaplastic lymphoma kinase
ANP	Atrial natriuretic peptide
AP-1	Activator protein 1
APCs	Antigen-presenting cells
Arg	L-arginine
ARG1	Arginase 1
ARL2	ADP-ribosylation factor-like protein 2
ASL	Argininosuccinate lyase
ASS	Argininosuccinate synthetase
ATF2	Activating transcription factor 2
AUF1 (hnRNP D)	AU-binding factor 1
BAFF-R	B-cell activating factor receptor
BCR	B-cell receptor
BH4	Tetrahydrobiopterin
BMDMs	Bone marrow-derived macrophages
C/EBPβ	CCAAT/enhancer-binding protein-β
CAC	Colitis-associated cancer
CaMK	Calcium/calmodulin-dependent protein kinase
CaSR	Ca^2+^-sensing receptor
CAT	Cationic amino acid transporter
CBP	CREB-binding protein
CD	Crohn’s disease
C/EBPβ	CCAAT/enhancer-binding protein element
cGAS-STING	‘Cyclic GMP-AMP synthase-stimulator of interferon genes’
CHIP	‘C-terminus of Hsc70-interacting protein’
CHOP	C/EBP homologous protein (Gad153)
cIAPs	Cellular inhibitors of apoptosis proteins
CLA	Conjugated linoleic acid
CLRs	C-type lectin receptors
CNC-bZIP	Cap’n’collar subfamily of the basic leucine zipper
COX2	Cyclooxygenase 2
CpG	Cytosine-phosphate-guanine
CPS	Carbamoyl phosphate synthetase
CRC	Colorectal cancer
CREB	cAMP-responsive element-binding protein
CRM1	Exportin 1
DAMPs	Damage-associated molecular patterns
DCs	Dendritic cells
DHPS	Deoxyhypusine synthase
DSS	Dextran sodium sulfate
DUOX2	Dual oxidase 2
EBP50	‘Ezrin-radixin-moesin-binding phosphoprotein 50’ protein
ECM	Extracellular matrix
ECs	Endothelial cells
ECS	‘Elongin–Cullin–SOCS-box’
EGCs	Enteric glial cells
EGF	Epithelial growth factor
EGFR	Epithelial growth factor receptor
Egr2	‘Early growth response factor 2’
eIF	Eukaryotic translation initiation factor
ELF	E74-like ETS transcription factor
ELK	ETS-like transcription factor
ENS	Enteric nervous system
ER	Endoplasmic reticulum
ERK	Extracellular-signal-regulated kinase
ETS	E26 transformation-specific
EZH2	‘Enhancer of zeste homolog 2’ histone methyltransferase
FBXO45	F-box/SPRY domain-containing protein 1
FOXO3	Forkhead box O3
FOXP3	Forkhead box P3
GAS	GAF activation sites
GSK3β	Glycogen synthase kinase 3 beta
HA	Hyaluronan
HB-EGF	Heparin-binding EGF-like growth factor
HDAC	Histone deacetylase
HIF	Hypoxia-inducible factor
HIMECs	Human intestinal microvascular endothelial cells
HMG	High mobility group
HSP	Heat shock protein
HRE	Hypoxia response elements
HuR	Human antigen R
HUVECs	Human umbilical vein endothelial cells
IBD	Inflammatory bowel disease
IECs	Intestinal epithelial cells
IELs	γδ intraepithelial lymphocytes
IFN-γ,	Interferon γ
IKK	IκB kinase
IL	Interleukin
ILCs	Innate lymphoid cells
IMFs	Intestinal myofibroblasts
IRAK	Interleukin-1 receptor-associated kinase
IRF	Interferon regulatory factor
IRF-E	IFN regulatory factor element
ISRE	Interferon-stimulated response element
IκB	Inhibitory κB
JAK	Janus kinases
JNK	c-Jun N-terminal kinase
KGF	Keratinocyte growth factor
KLF	Kruppel-like factor
KSRP	KH-type splicing regulatory protein
LAP	‘Liver-enriched activator protein’
LCN2	Lipocalin 2
LD	Lipid droplet
LIP	‘Liver-enriched inhibitory protein’
L-NAME	N-nitro-L-arginine methyl ester
LPS	Lipopolysaccharide
LRPPRC	Leucine-rich pentatricopeptide repeat protein
LTβR	Lymphotoxin β receptor
Maf	v-maf musculoaponeurotic fibrosarcoma oncogene family proteins
MAPK	Mitogen-activated protein kinase
MARs	Nuclear matrix attachment regions
MD-2	Myeloid differentiation factor 2
MDSCs	Myeloid-derived suppressor cells
MEG3	‘Maternally expressed gene 3’
MEK	Mitogen-activated protein kinase kinase
Mincle	‘Macrophage-inducible C-type lectin’ receptor
MSCs	Mesenchymal stem cells
MST2	‘Mammalian sterile 20-like kinase 2’
MTAP	Methylthioadenosine phosphorylase
MTP	5′-deoxy-5′-methylthioadenosine
MYCBP2	‘MYC Binding Protein 2’
Myd88	‘Myeloid differentiation primary response 88’
NAIP5	‘NLR family apoptosis inhibitory protein 5’
NAP110	NOS-associated protein 110 kDa
NEMO	NF-κB essential modulator
NETs	Neutrophil extracellular traps
NFAT	‘Nuclear factor of activated T cells’
NF-E2	Nuclear factor (erythroid 2)-like
NF-κB	Nuclear factor kappa-light-chain-enhancer of activated B cells
NIK	NF-κB–inducing kinase
NK	Natural killer cells
NLR	Nucleotide-binding domain and leucine-rich repeat-containing proteins
NLRC4	NLR family CARD domain-containing protein 4
NLRP3	NOD-, LRR- and pyrin domain-containing protein 3
NMD	Nonsense-mediated mRNA decay
NMMA	N^G^-monomethyl-L-arginine
NO	Nitric oxide
NOD	Nucleotide oligomerization domains
NOS	Nitric oxide synthase
NOS2P3	Nitric Oxide Synthase 2 Pseudogene 3
NOX2	NADPH oxidase 2
NRE	NF-E2 recognition element
NRE	Negative regulatory element for NF-κB
NRF	NF-κB-repressing factor
Oct	Octamer binding transcription factor
ODC	Ornithine decarboxylase
ORE	Oct-response elements
ORF	Open reading frame
PABP	Polypyrimidine-tract binding protein
PACAP	Pituitary adenylate cyclase–activating peptide
PARP1	Poly(ADP-ribose) polymerase-1
PBMCs	Peripheral blood mononuclear cells
PCs	Plasma cells
PCDP	Programmed cell death protein
PD-L	Programmed death-ligand
PERK	‘Pancreatic EIF-2alpha kinase’
PG	Prostaglandin
PI3K	Phosphoinositide 3-kinase
PIAS	Protein inhibitors of activated STAT
PIDs	Pyrimidine imidazole derivatives
PKA	Protein kinase A
PKC	Protein kinase C
PMA	Phorbol myristate acetate
PPAR	Peroxisome proliferator-activated receptor
PPREs	Peroxisome proliferator response elements
PRC1	Polycomb Repressive Complex 1
PRMT	Protein arginine methyltransferase
PRRs	Pattern recognition receptors
PTB	Polypyrimidine tract-binding protein
PTEN	Phosphatase and tensin homolog
PTPN2	Protein tyrosine phosphatase non-receptor type 2
RA	Retinoic acid
RAGE	Receptor for advanced glycation end products
RANKL	Receptor activator of NF-κB ligand
RAR, RXR	Retinoic acid receptors
RAREs	Retinoic acid response elements
RhoA	Ras homolog family member A
RIPK	Receptor-interacting protein kinase
RNCR3	‘Retinal non-coding RNA 3’
RONS	Reactive oxygen and nitrogen species
RUNX	Runt-related transcription factor
SCF	‘Skp–Cullin–F-box’
SCFAs	Short-chain fatty acids
SDMA	Symmetric dimethylarginine
SIRT	Sirtuin
SMAD	‘Suppressor of mothers against decapentaplegic homolog’
SMCs	Smooth muscle cells
SOCS	‘Suppressor of cytokine signaling’ protein
SPSB	‘SPRY domain-containing SOCS box’ protein
STAT	Signal transducer and activator of transcription
TAB	TAK1-binding protein
TAK1	TGF-β–activated kinase 1
TBE	T-cell factor (TCF) 4-binding element
TBK1	‘TANK-binding kinase 1’
TCF	T-cell transcription factor
TCR	T-cell receptor
TET	‘Ten-eleven translocation’ demethylase
TF	Transcription factor
TGFβ	Transforming growth factor β
TIAR	T cell intracellular antigen-1–related protein
TIGAR	TP53-induced glycolysis regulatory protein
TLRs	Toll-like receptors
TNBS	2,4,6-trinitrobenzene sulfonic acid
TNFα	Tumor necrosis factor α
TNFR	Tumor necrosis factor receptor
TRAFs	TNFR-associated factors
TRE	TNF response element
TRIB	Tribbles homolog
TTP	Tristetraprolin
TXNIP	Thioredoxin-interacting protein
uORF	Upstream open reading frame
UPR	Unfolded protein response
UC	Ulcerative colitis
UTR	Untranslated region
VEGFA	Vascular endothelial growth factor A
VIP	Vasointestinal peptide
VPAC	Vasoactive intestinal peptide receptor
WNK1	WNK lysine deficient protein kinase 1
Wnt	‘Wingless-related integration site’
XBP1	X-box binding protein 1
XPO	Exportin
